# Palladium-Catalyzed Oxidative Regio- and Diastereoselective Diarylating Carbocyclization of Dienynes

**DOI:** 10.1002/chem.201204555

**Published:** 2013-04-09

**Authors:** Min Jiang, Jan-E Bäckvall

**Affiliations:** [a] Department of Organic Chemistry, Arrhenius Laboratory, Stockholm University10691 Stockholm (Sweden), Fax: (+46) 8-154-908 E-mail: jeb@organ.su.se

**Keywords:** carbocyclization, dienynes, oxidation, palladium, synthetic methods

Development of effective cyclization reactions for the synthesis of carbocycles and heterocycles has been the subject of extensive study because of the relevance of cyclic structures to medicine and various functional materials.^1^ Transition-metal-promoted carbocyclizations of unsaturated functionalities have been demonstrated to provide convenient methods for the facile formation of cyclic structures.^2^ In 1990, Livinghouse and co-workers reported the highly successful rhodium-catalyzed intramolecular [4+2] cycloisomerization of dienynes,^3^ and since then a number of groups have been studying the cycloaddition of dienynes by the use of the metal catalysts of Rh and Au.^4^

Our research group has been involved in the development of various palladium-catalyzed carbocyclizations under oxidative conditions.^5^–^7^ In the 1990 s, our group reported a number of intramolecular palladium(II)-catalyzed 1,4-oxidations of conjugated dienes.^8^ In these reactions, combinations of halide, oxygen, and nitrogen nucleophiles are added across the diene. Attempts to extend these reactions to involve a carbon nucleophile as one of the nucleophiles were made, and when dienyne was employed in the presence of LiCl, a carbon and a chloride nucleophile were added across the diene (Figure 1 a).^6^ In the latter oxidative carbocyclization of dienynes, a vinylpalladium species was formed by chloropalladation of the alkyne. The vinylpalladium intermediates can trigger the cyclization reactions leading to intermediate **M**. The chloropalladation of the triple bond is nonstereoselective, whereas the overall 1,4-carbochlorination of the diene is stereoselective.

**Figure 1:**
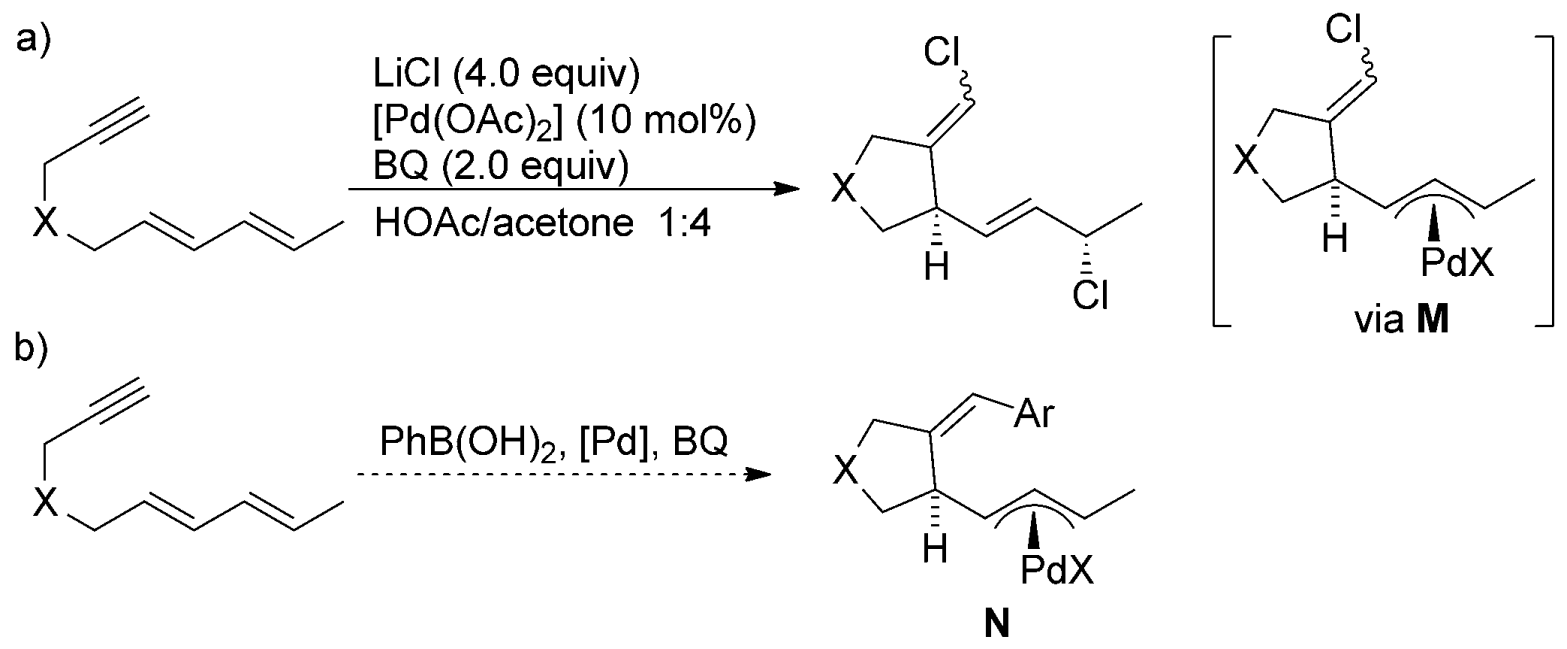
a) Oxidative Pd-catalyzed carbocyclization of dienynes with lithium chloride; and b) proposed intermediate for the carbocyclization of dienyne.

In recent work, we have developed the palladium-catalyzed oxidative carbocyclization/borylation and -arylation of enallenes,7a, b allenynes,7c and enynes,7d in which a diboron compound or an arylboronic acid was used to readily generate a boron–palladium or an arylpalladium intermediate from a Pd^II^ species. As an extension of our oxidative palladium chemistry, we envisioned that allylpalladium intermediate **N** could be formed through the carbocyclization of dienyne with Pd^II^, whereas arylpalladation occurs with *cis*-addition to the alkyne in the presence of arylboronic acid (Figure 1 b). Herein, we present a mild and efficient stereoselective diarylating carbocyclization of dienynes catalyzed by Pd^II^ under oxidative conditions to give the corresponding diarylated carbocycles with 1,2-oxidation and 1,4-oxidation of the conjugated diene.

In our preliminary experiments, *N*-tethered dienyne **1 a** was treated with 3 mol % of [Pd(OCOCF_3_)_2_], 3.0 equiv of phenylboronic acid (**2 a**) and 1.0 equiv of *p*-benzoquinone (BQ) in THF at room temperature. However, only [4+2] cycloaddition was observed, and full conversion of **1 a** was achieved in 16 h to give **3 a** in an isolated yield of 76 %. The ester-tethered dienyne **1 b** produced compound **3 ba**. When *O*-tethered dienyne **1 c** was employed as the substrate, the reaction gave the two cyclic diarylated regioisomers **3 ca** and **4 ca** in a ratio of 5:1 in 55 % yield (Scheme 1). The stereochemistry of **3 ca** and **4 ca** was established by comparing the coupling constant with analogous carbocyclization products from enynes.7d These experiments suggest that the oxygen tether is crucial for the diarylating carbocyclization.

**Scheme 1:**
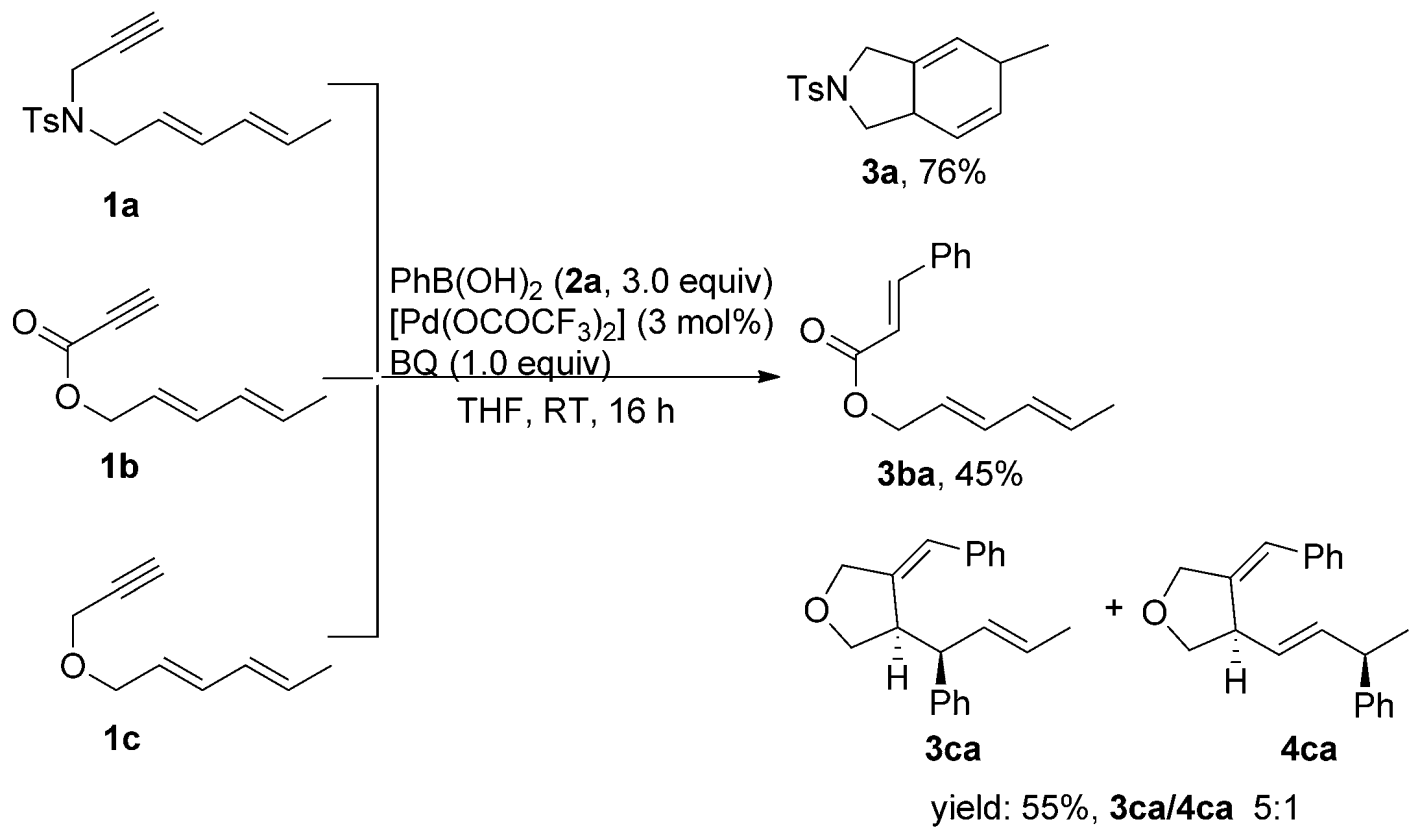
Reactions of dienynes with phenylboronic acid catalyzed by palladium(II).

Encouraged by these results, we synthesized dienyne **1 d** with a phenyl substituent on the diene and studied it in the diarylating carbocyclization. The two corresponding cyclic products **3 da** and **4 da** were obtained in 43 % yields with unreacted starting material and importantly the ratio of regioisomers **3 da**/**4 da** had now increased to 20:1. Furthermore, we screened a range of reaction parameters to find a suitable protocol for selective formation of cyclic diarylated products (Table S1 in the Supporting Information). It was observed that commercially available arylboronic acids contain its boronic anhydrides. However, only the free arylboronic acids can initiate this transformation. We found that DMSO and high temperature can promote the decomposition of boronic anhydrides to arylboronic acids. At the same time, DMSO could stabilize the palladium catalyst.^9^ Therefore, we added 3.0 equiv DMSO and ran the reaction at 50 °C. Full conversion of **1 d** was achieved in 4 h, and the cyclic diarylated products **3 da** and **4 da** were produced in a ratio of 15:1 in 66 % yield (Table 1, entry 1). The catalytic activity of various palladium(II) species differed and PdCl_2_, [PdCl_2_(PPh_3_)_2_], and [Pd(acac)_2_] (acac=acetylacetonate) failed to promote any arylation resulting in full recovery of the starting material. The use of [Pd(OAc)_2_] afforded the two cyclic diarylated compounds **3 da** and **4 da** in a ratio of 13:1 in 36 % yield. Further examination of solvent effects revealed that acetone, diethyl ether, 1,2-dichloroethane (DCE), toluene, and DMF gave lower yields, and no reaction was observed when acetonitrile was used as solvent. When DMSO was used as solvent, a nonselective reaction was obtained. Therefore, the optimal conditions were set to 3 mol % of [Pd(OCOCF_3_)_2_], 3.0 equiv of phenylboronic acid (**2 a**), 3.0 equiv of DMSO, and 1.0 equiv of BQ in THF at 50 °C.

**Table 1:** Scope of functionalized arylboronic acids.
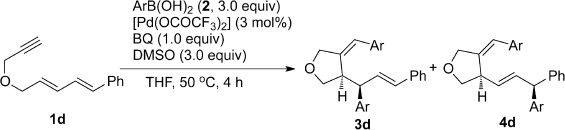

Entry^[a]^	ArB(OH)_2_, Ar	Yield [%]^[b]^ (3 d+4 d)/ ratio (3 d/4 d)
1	**2 a**, C_6_H_5_	66 (**3 da**/**4 da** 15:1)
2	**2 b**, 4-MeC_6_H_4_	72 (**3 db**/**4 db** 17:1)
3	**2 c**, 3-MeC_6_H_4_	73 (**3 dc**/**4 dc** 18:1)
4	**2 d**, 2-MeC_6_H_4_	73 (**3 dd**/**4 dd** 18:1)
5	**2 e**, 4-*t*BuC_6_H_4_	77 (**3 de**/**4 de** 22:1)
6	**2 f**, 4-TMSC_6_H_5_	68 (**3 df**/**4 df** 12:1)
7	**2 g**, 4-MeOC_6_H_4_	75 (**3 dg**/**4 dg** 11:1)
8	**2 h**, 4-vinylC_6_H_4_	68 (**3 dh**/**4 dh** 20:1)
9	**2 i**, 2-naphthyl	65 (**3 di**/**4 di** 20:1)
10	**2 j**, 3-furyl	67 (**3 dj**/**4 dj** 10:1)
11	**2 k**, 4-BrC_6_H_4_	58 (**3 dk**/**4 dk** 8.8:1)
12	**2 l**, 2-BrC_6_H_4_	61 (**3 dl**/**4 dl** 8.5:1)
13	**2 m**, 4-ClC_6_H_4_	60 (**3 dm**/**4 dm** 7.3:1)

[a] Reaction conditions: **1 d** (0.2 mmol), [Pd(OCOCF_3_)_2_] (0.006 mmol), BQ (0.2 mmol), DMSO (0.6 mmol), and arylboronic acid **2** (0.6 mmol) in THF (2.0 mL) at 50 °C for 4 h. [b] Isolated yields.

By using the optimized reaction conditions, the scope of arylboronic acids **2** was further examined. A variety of both electron-deficient and electron-rich arylboronic acids were evaluated, and the results are summarized in Table 1. The diarylating carbocyclization procedure tolerated a broad range of functional groups, and the electronic nature of the arylboronic acids **2** had some influence on the yield and regioselectivity of the reaction. Electron-rich arylboronic acids with an alkyl- (Table 1, entries 2–5), silyl- (entry 6), or alkoxy substituent (entry 7) proceeded well under the optimal reaction conditions. Additional olefin functionality was tolerated, and no cross-insertion was observed (Table 1, entry 8). Naphthylboronic acid (Table 1, entry 9) and heteroarylboronic acid (entry 10) also worked well and gave good yields of the diarylated products. With the above-mentioned arylboronic acids used (Table 1, entries 1–10) a highly regioselective carbo-arylation of the conjugated diene **1 d** took place affording compounds **3** and **4** in a good-to-high ratio (10:1–22:1). Halide-substituted arylboronic acids reacted smoothly with dienyne **1 d** to give the corresponding diarylated products **3** and **4** in moderate yields with slightly lower regioselectivity (Table 1, entries 11–13). A bromoaryl functionality, which is a labile moiety in Pd^0^-catalyzed cross-coupling reactions, showed good compatibility with the oxidative palladium conditions (Table 1, entries 11 and 12). The use of a bromo substitution allowed modification of the diarylated carbocycles. Unfortunately, attempts to obtain the diarylative carbocyclization with the (*E*)-styrylboronic acid were unsuccessful.

The reaction of different dienynes with phenylboronic acid was also investigated (Table 2). The reaction of dienyne **1 c** with monomethyl substitution afforded products **3 ca** and **4 ca** in a ratio of 3:1 in 68 % yield at 50 °C (Table 2, entry 1). The diarylating carbocyclization process of nonsubstituted dienyne **1 e** also gave the desired products at 50 °C. However, the ratio of **3 ea** and **4 ea** was 1.2:1. Attempts to improve the regioselectivity by changing the amount of phenylboronic acid and the concentration of the substrate were unsuccessful. Reaction at 30 °C increased the ratio of **3 ea** and **4 ea** to 1.5:1 (Table 2, entry 2). A further decrease of temperature led to very low conversion. With dimethyl-substituted dienyne **1 f** as substrate, the diarylated products **3 fa** and **4 fa** were obtained in a ratio of 4:1 in 63 % yield at 50 °C. The ratio of the regioisomers increased to 6:1 at room temperature (Table 2, entry 3). 1,6-Dienyne **1 d** afforded diarylated products **3 da** and **4 da** with high regioselectivity in a total yield of 66 % (Table 2, entry 4). Tolyl-substituted dienyne **1 g** gave the corresponding products **3 ga** and **4 ga** in a ratio of 10:1 and in 71 % yield (Table 2, entry 5). When a terminal substituent of the alkyne was introduced, the reaction was slower. Under the optimized reaction conditions, the desired diarylated products **3 ha** and **4 ha** were isolated in 73 % yield in a ratio of 12:1 (Table 2, entry 6). 1,7-Dienyne **1 i** showed lower activity under the general reaction condition, which may be attributed to the less favored coordination. By prolonging the reaction time to 6 h, the reaction of dienyne **1 i** with phenylboronic acid afforded the corresponding six-membered ring products **3 ia** and **4 ia** in a ratio of 8:1 in 68 % yield (Table 2, entry 7). Additional dimethyl substitution at the propargyl position had only a minor influence on the reaction outcome, and the diarylated products **3 ja** and **4 ja** were obtained in a ratio of 15:1 and in 65 % yield (Table 2, entry 8). The diphenyl substituted dienyne **1 k** underwent the carbocyclization/arylation sequence affording the products with high regioselectivity and gave the only regioisomer **3 ka** at 50 °C (Table 2, entry 9). Reaction of cyclic dieneyne **1 l** was unsuccessful and no carbocyclization product was formed.

**Table 2:** Reaction of dienynes 1 with phenylboronic acid.^[a]^

Entry	1	3	4	Yield3+4[%]^[b]^ (ratio3:4)
1	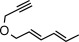	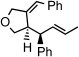	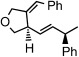	68 (3:1) 55 (5:1)^[c]^
	**1 c**	**3 ca**	**4 ca**	
2	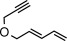	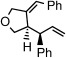	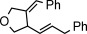	63 (1.2:1)^[d]^ 60 (1.5:1)^[e]^
	**1 e**	**3 ea**	**4 ea**	
3	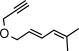	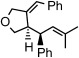	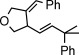	63 (4:1)^[d]^ 52 (6:1)^[c]^
	**1 f**	**3 fa**	**4 fa**	
4	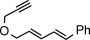	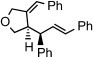	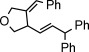	66 (15:1)
	**1 d**	**3 da**	**4 da**	
5	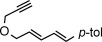	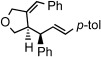	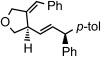	71 (10:1)
	**1 g**	**3 ga**	**4 ga**	
6	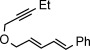	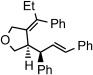	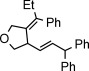	73 (10:1)
	**1 h**	**3 ha**	**4 ha**	
7	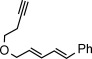	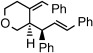	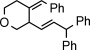	68 (8:1)^[f]^
	**1 i**	**3 ia**	**4 ia**	
8	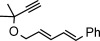	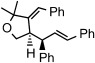	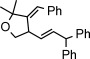	65 (15:1)
	**1 j**	**3 ja**	**4 ja**	
9	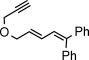	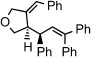	–	72 (>99:1)
	**1 k**	**3 ka**		
10	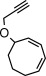	–	–	–
	**1 l**			

[a] Reaction conditions: **1** (0.2 mmol), [Pd(OCOCF_3_)_2_] (0.006 mmol), BQ (0.2 mmol), DMSO (0.6 mmol), and arylboronic acid **2** (0.6 mmol) in THF (2.0 mL) at 50 °C for 4 h. [b] Isolated yields. [c] The reaction time was 20 h at RT. [d] The reaction time was 5 h. [e] The temperature was 30 °C. [f] The reaction time was 6 h.

We next examined Pd^II^-catalyzed oxidative diarylating carbocyclization of dienynes by a biomimetic approach.5c, 10 The reaction of dienyne **1 d** with [Pd(OCOCF_3_)] (3 mol %), BQ (20 mol %), iron phthalocyanine [Fe(Pc)] (2 mol %), DMSO (3.0 equiv), and PhB(OH)_2_ (3.0 equiv) in THF under 1 atm oxygen at 50 °C for 16 h gave the diarylated products **3 da** and **4 da** in a ratio of 16:1 in 71 % yield (Scheme 2).

**Scheme 2:**
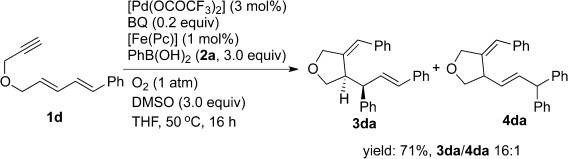
Reaction of dieneyne **1 d** by a biomimetic approach.

Mechanistically, we postulate a reaction pathway based on our previous results (Scheme 3).8d A fast transmetalation between the arylboronic acid and the Pd^II^ catalyst generates an ArPdX species,^11^ which adds to the alkyne in a *syn*-arylpalladation. The vinylpalladium intermediate **A** formed^**[**12]^ subsequently undergoes a carbocyclization, in which the diene inserts into the vinyl–Pd bond to give π-allylpalladium intermediate **N**.^13^ Transmetallation of cyclic intermediate **N** with a second arylboronic acid occurs to give intermediate **B**. However, intermediate **B** is stable towards reductive elimination, but is in an equilibrium with intermediates **C** and **D**.^14^ The formation of diarylated compounds **3** and **4** with retention of configuration at carbon occurs via the reductive elimination of intermediates **C** and **D**, respectively, by coordination of BQ.^15^ The released Pd^0^ is reoxidized to Pd^II^ by the coordinated BQ. Intermediate **C** is favored over intermediate **D** due to coordination of the olefin to Pd in the former, and when R^2^ is an aryl group, intermediate **C** is much more stable then intermediate **D**. The preference for **C** over **D** would explain the high regioselectivity obtained. If R^2^ is a proton or alkyl group, it would be easier to form intermediate **D**, which leads to lower regioselectivity.

**Scheme 3:**
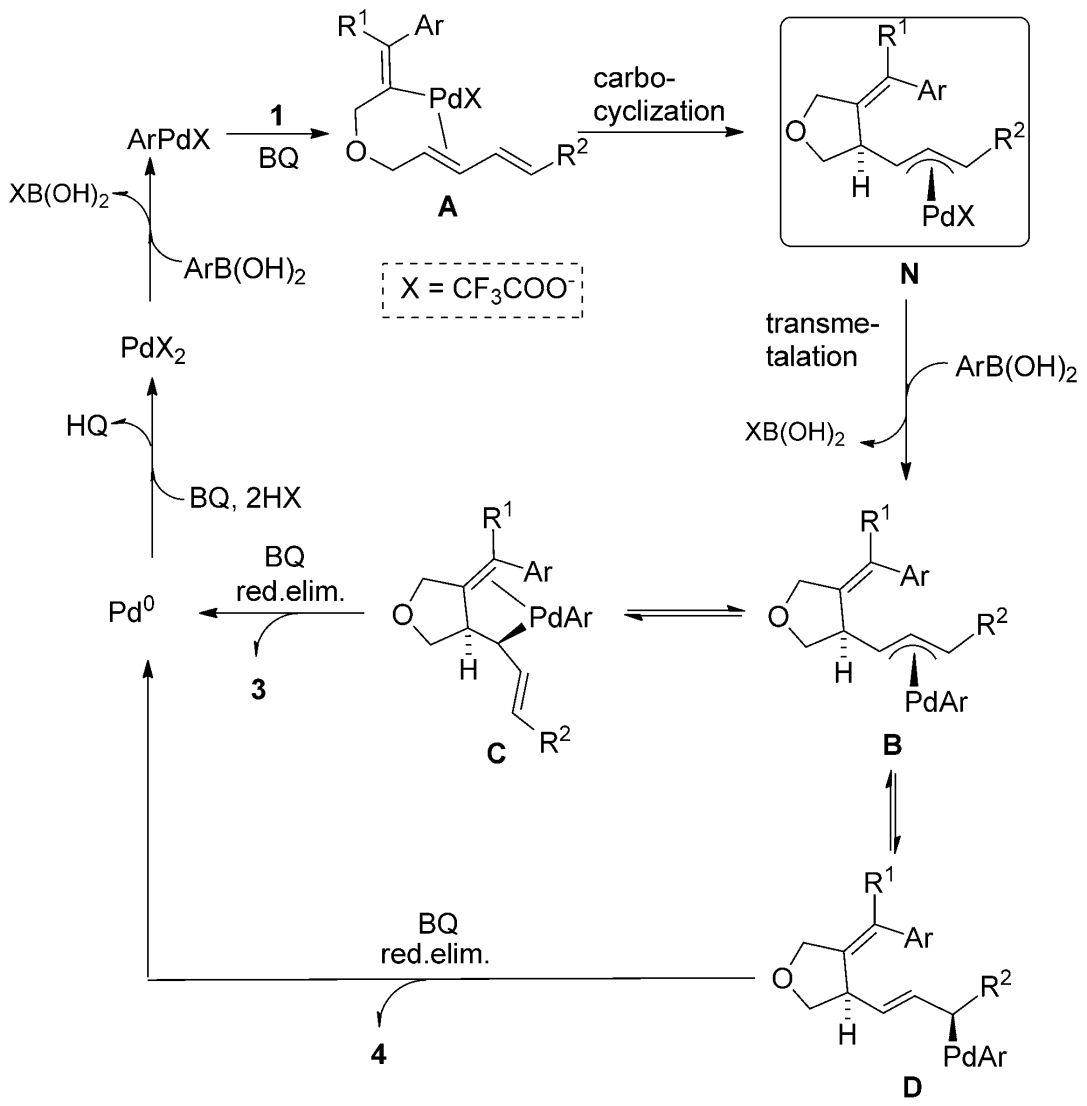
Proposed mechanism for Pd-catalyzed oxidative regioselective diarylating carbocyclization of dienynes.

In conclusion, a mild and efficient Pd^II^-catalyzed oxidative regioselective diarylating carbocyclization of dienynes was developed by using arylboronic acids with stereoselective formation of tetrahydrofurans and tetrahydropyrans. The high stereo- and regioselectivity of the addition across the conjugated diene involving the carbocyclization has been investigated. Further studies regarding the scope, mechanism, and synthetic application of this reaction are currently underway in our laboratory.

## Experimental Section

**General procedure for the oxidative diarylating carbocyclization of dienyne**: To a solution of dienyne **1** (0.2 mmol) in THF (2 mL) was added [Pd(OOCCF_3_)_2_] (2 mg, 0.006 mmol, 3 mol %), DMSO (52 mg, 0.6 mmol), BQ (23.6 mg, 0.2 mmol), and ArB(OH)_2_ (0.6 mmol). The mixture was stirred at 50 °C for 4 h. The reaction mixture was then cooled to RT, the solvent was evaporated, and the residue was purified by flash-column chromatography (pentane/ethyl acetate 100:1) to give the diarylated regioisomers **3** and **4**.

## Supplementary material

As a service to our authors and readers, this journal provides supporting information supplied by the authors. Such materials are peer reviewed and may be re-organized for online delivery, but are not copy-edited or typeset. Technical support issues arising from supporting information (other than missing files) should be addressed to the authors.
