# Supplementary material for: Palladium-Catalyzed Oxidative Regio- and Diastereoselective Diarylating Carbocyclization of Dienynes
Source: Chemistry. 2013 Apr 9;19(21):6571–5. doi: 10.1002/chem.201204555 (PMC3743347; doi:10.1002/chem.201204555)
Supplement: Supplementary file 1 [file chem0019-6571-SD1.pdf]

CHEMISTRY
A EUROPEAN JOURNAL

Supporting Information

© Copyright Wiley-VCH Verlag GmbH & Co. KGaA, 69451 Weinheim, 2013

Palladium-Catalyzed Oxidative Regio- and Diastereoselective Diarylating Carbocyclization of Dienynes

Min Jiang and Jan-E. Bäckvall*[a]

chem_201204555_sm_miscellaneous_information.pdf

Supporting Information

Palladium-Catalyzed Oxidative Regio- and Diastereoselective Diarylating Carbocyclization of Dienynes

Min Jiang† and Jan-E. Bäckvall†*

†*Department of Organic Chemistry, Arrhenius Laboratory, Stockholm University SE-106 91 Stockholm, Sweden*

jeb@organ.su.se

Contents

General information ……………………………………………………………S2

Preparation of starting materials ……………………………………………………….S3

Selected optimization results …………………………………………………………..S6

General procedures and spectral data ……………………...……………………..S8

Copies of spectra …………………………………………………………………...S15

Experimental section

General information

Unless otherwise noted, all reagents were used as received from commercial suppliers. $Pd(OOCCF_3)_2$ was obtained from Pressure Chemicals and used without further purification. All the arylboronic acids were purchased from Sigma-Aldrich Co. LLC. Palladium-Catalyzed Oxidative Regio- and Diastereoselective Diarylating Carbocyclization of dienynes was performed without any efforts to exclude moisture, i.e. reagent grade THF can be used without any drying/purification. The H_2O content of this THF was typically 50-150 ppm, measured using a Metrohm 831F (KF-titrator). Dry solvents for other necessary reactions (THF) were obtained from a VAC Solvent Purifier. Reactions were monitored using thin-layer chromatography (SiO_2). TLC plates were visualized with UV light (254 nm), iodine treatment or using Hanessians stain. Flash chromatography was carried out with 60Å (particle size 35-70 µm) normal flash silica gel. NMR spectra were recorded at 400 MHz (H) and at 100 MHz (C), respectively. Chemical shifts (δ) are reported in ppm, using the residual solvent peak in $CDCl_3$ (H: δ = 7.26 and C: δ = 77.0 ppm) as internal standard, and coupling constants (*J*) are given in Hz. HRMS were recorded using ESI-TOF techniques.

Preparation of starting materials

Dienyne substrates **1a-1l** were prepared as reported in the literature.[1] Characterizations of dienyne **1** are given below:

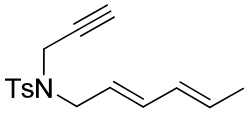

1a

A white solid; ^{1}**H NMR** ($CDCl_3$, 400 MHz): δ 7.75 (d, J = 8.4 Hz, 2H), 7.31 (d, J = 8.4 Hz, 2H), 6.20 (dd, J = 15.2, 10.4 Hz, 1H), 6.01-6.08 (m, 1H), 5.68-5.77 (m, 1H), 5.39-5.46 (m, 1H), 4.09 (d, J = 2.8 Hz, 2H), 3.84 (d, J = 7.2 Hz, 1H), 2.45 (s, 1H), 2.02 (t, J = 2.4 Hz, 1H), 1.77 (d, J = 2.8 Hz, 3H).

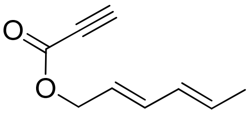

1b

A colorless oil; ^{1}**H NMR** ($CDCl_3$, 400 MHz): δ 6.28 (dd, J = 15.2, 10.4 Hz, 1H), 6.02-6.09 (m, 1H), 5.76-5.81 (m, 1H), 5.58-5.66 (m, 1H), 4.68 (d, J = 6.8 Hz, 2H), 2.87 (s, 1H), 1.77 (dt, J = 6.8, 0.8 Hz, 3H).

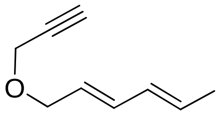

1c

A colorless oil; ^{1}**H NMR** ($CDCl_3$, 400 MHz): δ 6.23 (dd, J = 15.2, 10.4 Hz, 1H), 6.03-6.10 (m, 1H), 5.69-5.75 (m, 1H), 5.56-5.64 (m, 1H), 4.13 (d, J = 2.4 Hz, 2H), 4.08 (d, J = 6.4 Hz, 2H), 2.41 (t, J = 2.4 Hz, 1H), 1.75 (dd, J = 2.4, 0.8 Hz, 3H).

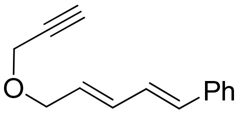

1d

A yellow solid; ^{1}**H NMR** ($CDCl_3$, 400 MHz): δ 7.41-7.44 (m, 2H), 7.32-7.36 (m, 2H), 7.25-7.28 (m, 1H), 6.81 (dd, J = 15.6, 10.4 Hz, 1H), 6.59 (d, J = 16.0 Hz, 1H), 6.48 (dd, J = 15.2, 11.2 Hz, 1H), 5.89 (dt, J = 15.2, 6.4 Hz, 1H), 4.20 (d, J = 2.4 Hz, 2H), 4.19 (d, J = 6.4 Hz, 2H), 2.47 (t, J = 2.4 Hz, 1H).

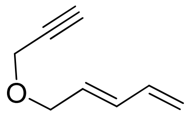

1e

(1) a) Ni, Y.; Montgomery, J. *J. Am. Chem. Soc.* **2004**, *126*, 11162-11163; b) Paik. S.; Son, S. U.; Chung, Y. K. *Org. Lett.* **1999**, *1*, 2045-2047.

A colorless oil; ^{1}**H NMR** ($CDCl_3$, 400 MHz): δ 6.27-6.42 (m, 1H), 5.76-5.82 (m, 1H), 5.23-5.28 (m, 1H), 5.12-5.15 (m, 1H), 4.17 (d, J = 2.4 Hz, 2H), 4.12 (d, J = 0.8 Hz, 2H), 2.45 (t, J = 2.4 Hz, 1H).

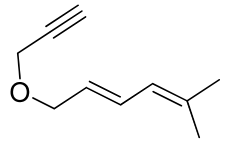

1f

A colorless oil; ^{1}**H NMR** ($CDCl_3$, 400 MHz): δ 6.50 (dd, J = 15.2, 11.2 Hz, 1H), 5.86 (dd, J = 11.2, 0.8 Hz, 1H), 5.59-5.66 (m, 1H), 4.16 (d, J = 2.4 Hz, 2H), 4.13 (d, J = 6.8 Hz, 2H), 2.45 (t, J = 2.0 Hz, 1H), 1.81 (s, 3H), 1.79 (s, 3H).

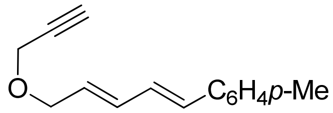

1g

A yellow solid; ^{1}**H NMR** ($CDCl_3$, 400 MHz): δ 7.29 (d, J = 6.8 Hz, 2H), 7.12 (d, J = 6.8 Hz, 2H), 6.74 (dd, J = 12.4, 8.4 Hz, 1H), 6.54 (d, J = 12.4 Hz, 1H), 6.41-6.46 (m, 1H), 5.84 (dt, J = 12.4, 5.2 Hz, 1H), 4.16-4.18 (m, 4H), 2.44 (t, J = 2.0 Hz, 1H). 2.34 (s, 3H).

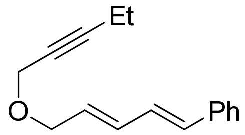

1h

A yellow solid; ^{1}**H NMR** ($CDCl_3$, 400 MHz): δ 7.41-7.44 (m, 2H), 7.32-7.36 (m, 2H), 7.23-7.28 (m, 1H), 6.81 (dd, J = 15.6, 10.4 Hz, 1H), 6.58 (d, J = 15.6 Hz, 1H), 6.46 (dd, J = 15.6, 10.4 Hz, 1H), 5.90 (dt, J = 15.6, 6.4 Hz, 1H), 4.16-4.18 (m, 4H), 2.24-2.31 (m, 2H), 1.19 (t, J = 7.6 Hz, 3H).

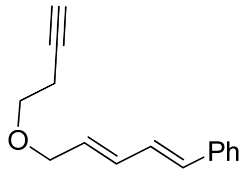

1i

A yellow solid; ^{1}**H NMR** ($CDCl_3$, 400 MHz): δ 7.41-7.44 (m, 2H), 7.32-7.36 (m, 2H), 7.25-7.28 (m, 1H), 6.81 (dd, J = 16.0, 10.4 Hz, 1H), 6.58 (d, J = 16.0 Hz, 1H), 6.42-6.48 (m, 1H), 5.88-5.94 (m, 1H), 4.14 (dd, J = 2.4, 1.2 Hz, 2H), 3.62 (t, J = 6.8 Hz, 2H), 2.53 (td, J = 6.8, 2.8 Hz, 2H), 2.04 (t, J = 2.4 Hz, 1H).

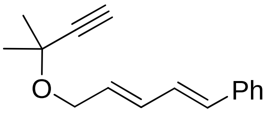

1j

A yellow solid; **^{1}H NMR** ($CDCl_3$, 400 MHz): δ 7.41-7.44 (m, 2H), 7.32-7.36 (m, 2H), 7.25-7.28 (m, 1H), 6.81 (dd, J = 15.6, 10.4 Hz, 1H), 6.56 (d, J = 15.6 Hz, 1H), 6.46 (dd, J = 15.2, 10.4 Hz, 1H), 5.94 (tdd, J = 15.6, 6.8, 0.8 Hz, 1H), 4.22 (d, J = 1.2 Hz, 2H), 2.47 (s, 1H), 1.56 (s, 6H).

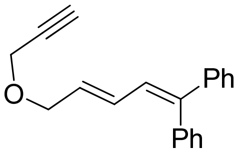

1k

A yellow solid; **^{1}H NMR** ($CDCl_3$, 400 MHz): δ 7.37-7.44 (m, 3H), 7.27-7.32 (m, 5H), 7.23-7.25 (m, 2H), 6.73 (d, J = 11.2 Hz, 1H), 6.36-6.43 (m, 1H), 5.93-6.00 (m, 1H), 4.11-4.15 (m, 2H), 4.09 (d, J = 1.2 Hz, 2H), 2.43 (t, J = 2.4 Hz, 1H).

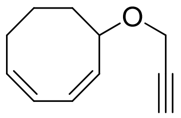

1l

A colorless oil; **^{1}H NMR** ($CDCl_3$, 400 MHz): δ 6.00-6.06 (m, 1H), 5.85-5.89 (m, 1H), 5.68-5.72 (m, 2H), 5.50-5.55 (m, 1H), 4.22 (dd, J = 15.6, 2.4 Hz, 1H), 4.09 (dd, J = 15.6, 2.4 Hz, 1H), 2.39 (t, J = 2.4 Hz, 1H), 2.26-2.31 (m, 1H), 1.88-1.91 (m, 1H), 2.00-2.08 (m, 2H), 1.41-1.45 (m, 2H).

Optimization of reaction conditions for diarylative carbocyclization of dienyne 1d:

Table SI-1. Optimization of the reaction conditions for the palladium-catalyzed oxidative diarylating carbocyclization of dienyne 1d

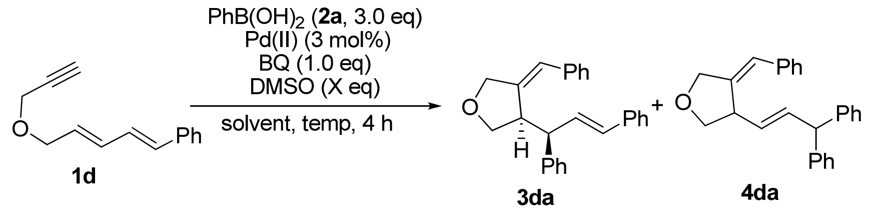

Entry[a]	Pd(II)	Temp (°C)	solvent	X (eq)	yield (%)[d]/ (3da+4da)	Ratio[e]/ 3da:4da
1[b]	$Pd(OCOCF_3)_2$	rt	THF	0	43	20:1
2[b, c]	$PdCl_2$	rt	DMF	0	0	-
3[b, c]	$PdCl_2(PPh_3)_2$	rt	DMF	0	0	-
4[b, c]	$Pd(acac)_2$	rt	THF	0	0	-
5	$Pd(OAc)_2$	rt	THF	0	36	13:1
6	$Pd(OCOCF_3)_2$	50	THF	3	66	15:1
7[b, c]	$PdCl_2$	50	THF	3	0	-
8[b, c]	$PdCl_2(PPh_3)_2$	50	THF	3	0	-
9[b, c]	$Pd(acac)_2$	50	THF	3	0	-
10[c]	$Pd(OCOCF_3)_2$	50	MeCN	3	0	-
11	$Pd(OCOCF_3)_2$	50	toluene	3	51	11:1
12[b]	$Pd(OCOCF_3)_2$	30	acetone	3	42	20:1
13	$Pd(OCOCF_3)_2$	50	DMF	3	trace	-
14[b]	$Pd(OCOCF_3)_2$	30	Et_2O	3	36	12:1
15	$Pd(OCOCF_3)_2$	50	DCE	3	54	8:1
16	$Pd(OCOCF_3)_2$	50	DME	3	61	18:1
17	$Pd(OCOCF_3)_2$	50	DMSO	3	0	-

[a] Reaction conditions: **1d** (0.2 mmol), [Pd] (0.006 mmol), oxidant (0.2 mmol), and phenylboronic acid (0.6 mmol), DMSO (X mmol), solvent (2.0 mL) at above temperature. [b] The reaction time was 16 hours. [c] No reaction. [d] Isolated yields. [e] Based on crude ^{1}H NMR.

We screened a range of reaction parameters to find a suitable protocol for selective formation of cyclic diarylated products (Table SI-1). The two corresponding cyclic products **3da** and **4da** were obtained in 43% yields with unreacted starting material and importantly the ratio of regioisomers **3da**:**4da** was 20:1. The catalytic activity of various palladium(II) species differed and $PdCl_2$, $PdCl_2(PPh_3)_2$ and $Pd(acac)_2$ failed to promote any arylation resulting in full recovery of the starting material. $Pd(OAc)_2$ afforded the two cyclic diarylated compounds **3da** and **4da** in a ratio of 13:1 in 36% yield. As we have observed, commercially available

arylboronic acids contain its boronic anhydrides. However, only the free arylboronic acids can initiate this transformation. We found that DMSO and high temperatures can promote the decomposition of boronic anhydrides to arylboronic acids. At the same time DMSO could stabilize the palldium catalyst. We therefore added 3.0 equiv DMSO and ran the reaction at 50 ^{o}C. Full conversion of **1d** was achieved in 4 hours and the cyclic diarylated products **3da** and **4da** were produced in a ratio of 15:1 in 66% yield (Table SI-1, entry 6). Further examination of solvent effects revealed that acetone, diethyl ether, 1,2-dichloroethane (DCE), toluene and DMF gave lower yields and no reaction was observed with acetonitrile as solvent. When DMSO was used as solvent, a non-selective reaction was obtained. Therefore, the optimal conditions were set to 3 mol% of $Pd(OCOCF_3)_2$, 3.0 equiv of phenylboronic acid (**2a**), 3.0 equiv of DMSO and 1.0 equiv of BQ in THF at 50 ^{o}C .

General procedure for the oxidative diarylating carbocyclization of dienyne 1

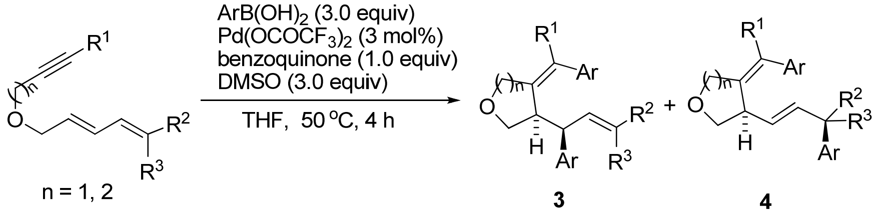

To a solution of dienyne **1** (0.2 mmol) in THF (2 mL) was added Pd($OOCCF_3$)$_2$ (2 mg, 0.006 mmol, 3 mol%), BQ (23.6 mg, 0.2 mmol), DMSO (52 mg), and ArB(OH)$_2$ (0.6 mmol). The mixture was stirred at 50 °C for 4 hours. The solvent was evaporated and the residue was purified by flash column chromatography (pentane/ethyl acetate v/v 100:1) giving a mixture of diarylated product **3** and **4**.

Spectral data of compounds 3, 4 (NMR and HRMS)

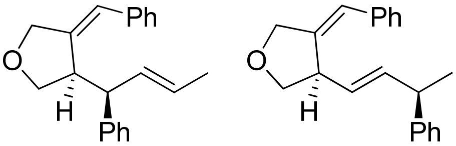

3ca **4ca**

Compound **3ca**: **^{1}H NMR** ($CDCl_3$, 400 MHz): δ 7.32-7.36 (m, 2H), 7.21-7.28 (m, 6H), 7.10-7.17 (m, 2H), 6.40 (d, J = 2.0 Hz, 1H), 5.76 (ddq, J = 14.8, 9.2, 1.6 Hz, 1H), 5.34 (dqd, J = 14.8, 6.4, 0.4 Hz, 1H), 4.49 (dt, J = 12.8, 2.0 Hz, 1H), 4.40 (dd, J = 12.8, 2.0 Hz, 1H), 4.19 (dd, J = 9.0, 2.4 Hz, 1H), 3.89 (dd, J = 9.0, 6.4 Hz, 1H), 3.67 (dd, J = 9.2, 4.8 Hz, 1H), 3.56-3.60 (m, 1H), 1.68 (dd, J = 6.4, 1.6 Hz, 3H). **^{13}C NMR** ($CDCl_3$, 100 MHz): δ 143.8, 143.5, 137.3, 129.3, 128.5, 128.4, 128.1, 127.9, 127.6, 126.6, 126.2, 120.7, 73.3, 71.4, 48.6, 47.0, 18.0.

Compound **4ca**: **^{1}H NMR** ($CDCl_3$, 400 MHz): δ 7.32-7.36 (m, 2H), 7.21-7.28 (m, 6H), 7.10-7.17 (m, 2H), 6.42 (d, J = 1.6 Hz, 1H), 5.71 (ddd, J = 15.6, 6.8, 1.2 Hz, 1H), 5.50 (ddd, J = 15.6, 7.2, 1.2 Hz, 1H), 4.58 (dt, J = 13.2, 2.0 Hz, 1H), 4.45 (dd, J = 13.2, 2.0 Hz, 1H), 4.15 (m, 1H), 4.02 (dd, J = 8.4, 6.4 Hz, 1H), 3.85-3.88 (m, 1H), 3.70-3.72 (m, 1H), 1.14 (d, J = 7.2 Hz, 3H).

Mixture of **3ca** and **4ca**: **HRMS** (ESI) m/z for $C_{21}H_{23}O$ $[M+H]^+$ calcd 291.1749, found 291.1751.

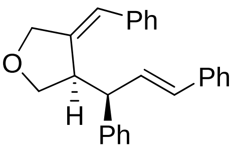

3da

A white solid; **^{1}H NMR** ($CDCl_3$, 400 MHz): δ 7.20-7.31 (m, 15H), 6.56 (dd, J = 15.6, 9.1 Hz, 1H), 6.43 (d, J = 1.6 Hz, 1H), 6.26 (d, J = 15.6 Hz, 1H), 4.52 (dt, J = 12.8, 1.6 Hz, 1H), 4.42

(dd, J = 12.8, 1.6 Hz, 1H), 4.28 (dd, J = 9.3, 2.0 Hz, 1H), 3.95 (dd, J = 9.3, 6.8 Hz, 1H), 3.81-3.84 (m, 2H). **^{13}C NMR** ($CDCl_3$, 100 MHz): δ 143.6, 142.9, 137.5, 137.3, 132.2, 128.5, 128.45, 128.43, 128.0, 127.6, 127.2, 126.8, 126.4, 126.3, 120.9, 73.3, 71.3, 48.7, 47.1. **HRMS** (ESI) m/z for $C_{25}H_{25}O$ $[M+H]^+$ calcd 353.1900, found 353.1905.

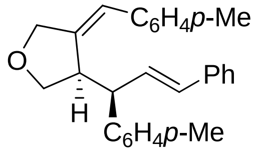

3db

A colorless oil; **^{1}H NMR** ($CDCl_3$, 400 MHz): δ 7.19-7.37 (m, 9H), 7.09-7.14 (m, 4H), 6.53 (dd, J = 15.6, 9.0 Hz, 1H), 6.38 (d, J = 1.2 Hz, 1H), 6.25 (d, J = 15.6 Hz, 1H), 4.48 (dd, J = 13.2, 1.2 Hz, 1H), 4.38 (dd, J = 13.2, 2.0 Hz, 1H), 4.27 (dd, J = 9.2, 2.0 Hz, 1H), 3.93 (dd, J = 9.2, 6.4 Hz, 1H), 3.82-3.85 (m, 2H), 2.41 (s, 3H), 2.34 (s, 3H). **^{13}C NMR** ($CDCl_3$, 100 MHz): δ 142.7, 140.0, 137.6, 136.5, 136.0, 134.3, 132.0, 129.2, 129.1, 128.7, 128.4, 127.9, 127.5, 127.1, 126.3, 120.6, 73.4, 71.3, 48.2, 47.1, 21.2, 21.0. **HRMS** (ESI) m/z for $C_{28}H_{29}O$ $[M+H]^+$ calcd 381.2213, found 381.2215.

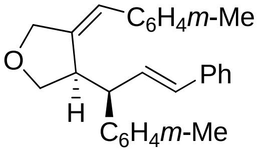

3dc

A colorless oil; **^{1}H NMR** ($CDCl_3$, 400 MHz): δ 7.18-7.42 (m, 9H), 7.11 (d, J = 7.6 Hz, 1H), 7.03-7.06 (m, 3H), 6.68 (dd, J = 15.6, 9.2 Hz, 1H), 6.40 (d, J = 1.2 Hz, 1H), 6.28 (d, J = 15.6 Hz, 1H), 4.53 (d, J = 13.2 Hz, 1H), 4.42 (dd, J = 13.2, 1.6 Hz, 1H), 4.31 (dd, J = 9.2, 1.6 Hz, 1H), 3.95 (dd, J = 9.2, 6.8 Hz, 1H), 3.81-3.84 (m, 2H), 2.43 (s, 3H), 2.35 (s, 3H). **^{13}C NMR** ($CDCl_3$, 100 MHz): δ 143.4, 143.0, 138.0, 137.9, 137.6, 137.2, 132.0, 128.7, 128.6, 128.4, 128.39, 128.37, 128.33, 127.5, 127.12, 127.11, 126.3, 125.3, 124.6, 120.8, 73.3, 71.3, 48.8, 47.4, 21.5, 21.4. **HRMS** (ESI) m/z for $C_{28}H_{29}O$ $[M+H]^+$ calcd 381.2213, found 381.2211.

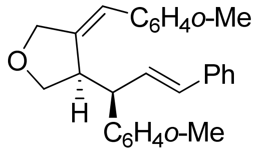

3dd

A colorless oil; **^{1}H NMR** ($CDCl_3$, 400 MHz): δ 7.18-7.35 (m, 9H), 7.01-7.04 (m, 3H), 6.93-6.97 (m, 1H), 6.56 (dd, J = 16.0, 9.2 Hz, 1H), 6.44 (dd, J = 1.6 Hz, 1H), 6.28 (d, J = 16.0 Hz, 1H), 4.62 (dt, J = 12.8, 2.0 Hz, 1H), 4.47 (dd, J = 12.8, 2.0 Hz, 1H), 4.33 (dd, J = 9.0, 2.4 Hz, 1H), 3.93 (dd, J = 9.0, 6.2 Hz, 1H), 3.79 (dd, J = 9.2, 6.0 Hz, 1H), 3.56-3.58 (m, 1H), 2.11 (s, 3H), 1.97 (s, 3H). **^{13}C NMR** ($CDCl_3$, 100 MHz): δ 143.5, 140.8, 137.4, 136.9, 136.3, 135.5, 131.6, 130.4, 129.9, 129.7, 128.4, 128.3, 127.3, 127.1, 127.0, 126.2, 125.9, 125.8, 125.7, 120.5, 72.5, 71.3, 45.6, 44.7, 19.8, 19.1. **HRMS** (ESI) m/z for $C_{28}H_{29}O$ $[M+H]^+$ calcd 381.2213, found 381.2213.

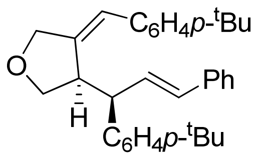

3de

A colorless oil; **^{1}H NMR** ($CDCl_3$, 400 MHz): δ 7.34-7.36 (m, 4H), 7.32-7.34 (m, 6H), 7.20-7.28 (m, 3H), 6.60 (dd, J = 15.6, 9.6 Hz, 1H), 6.40 (d, J = 1.6 Hz, 1H), 6.32 (d, J = 15.6 Hz, 1H), 4.53 (dt, J = 13.2, 1.6 Hz, 1H), 4.42 (dd, J = 13.2, 1.6 Hz, 1H), 4.32 (dd, J = 9.0, 1.6 Hz, 1H), 3.96 (dd, J = 9.0, 6.0 Hz, 1H), 3.85-3.91 (m, 2H), 1.42 (m, 9H), 1.36 (s, 9H). **^{13}C NMR** ($CDCl_3$, 100 MHz): δ 158.3, 158.1, 141.5, 137.6, 135.0, 131.9, 129.9, 129.2, 129.0, 128.6, 128.4, 127.1, 126.3, 120.3, 113.9, 113.8, 73.3, 71.4, 55.3, 55.2, 47.9, 47.0. **HRMS** (ESI) *m/z* for $C_{34}H_{44}ON$ $[M+NH_4]^+$ calcd 482.3417, found 482.3339.

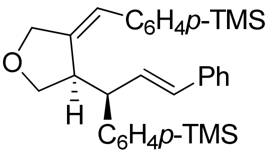

3df

A white solid; **^{1}H NMR** ($CDCl_3$, 400 MHz): δ 7.43 (d, J = 7.6 Hz, 2H), 7.36 (d, J = 7.6 Hz, 2H), 7.26-7.32 (m, 6H), 7.20-7.03 (m, 3H), 6.55 (dd, J = 15.6, 9.2 Hz, 1H), 6.39 (s, 1H), 6.26 (d, J = 15.6, 1H), 4.50 (d, J = 13.2 Hz, 1H), 4.39 (dd, J = 13.2, 1.6 Hz, 1H), 4.28 (d, J = 9.6 Hz, 1H), 3.94 (dd, J = 9.6, 6.3 Hz, 1H), 3.82-3.86 (m, 2H), 0.32 (s, 9H), 0.27 (s, 9H). **^{13}C NMR** ($CDCl_3$, 100 MHz): δ 143.8, 143.5, 139.0, 138.2, 137.5, 133.5, 132.2, 128.5, 128.4, 127.4, 127.2, 127.1, 126.3, 120.9, 73.4, 71.4, 48.8, 47.1, -1.08, -1.10. **HRMS** (ESI) *m/z* for $C_{32}H_{41}OSi_2$ $[M+H]^+$ calcd 497.2690, found 497.2691.

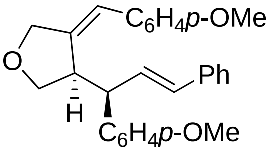

3dg

A colorless oil; **^{1}H NMR** ($CDCl_3$, 400 MHz): δ 7.23-7.38 (m, 8H), 7.14 (d, J = 8.4 Hz, 2H), 6.92 (d, J = 8.4 Hz, 2H), 6.83 (d, J = 8.4 Hz, 2H), 6.52 (dd, J = 15.6, 9.2 Hz, 1H), 6.35 (d, J = 1.6 Hz, 1H), 6.24 (d, J = 15.6 Hz, 1H), 4.49 (d, J = 12.8 Hz, 1H), 4.39 (dd, J = 12.8, 1.6 Hz, 1H), 4.26 (dd, J = 8.8, 1.6 Hz, 1H), 3.95 (dd, J = 8.8, 6.0 Hz, 1H), 3.87 (s, 3H), 3.77-3.84 (m, 2H), 3.81 (s, 3H). **^{13}C NMR** ($CDCl_3$, 100 MHz): δ 149.7, 149.1, 142.8, 139.9, 137.6, 134.3, 131.9, 128.8, 128.4, 127.8, 127.3, 127.1, 126.3, 125.4, 125.3, 120.6, 73.3, 71.4, 48.4, 47.1, 34.5, 34.3, 31.3. **HRMS** (ESI) *m/z* for $C_{28}H_{29}O_3$ $[M+H]^+$ calcd 413.2111, found 413.2111.

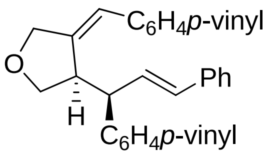

3dh

A colorless oil; **^{1}H NMR** ($CDCl_3$, 400 MHz): δ 7.42 (d, J = 8.4 Hz, 2H), 7.28-7.36 (m, 9H), 7.16 (d, J = 8.4 Hz, 2H), 6.76 (dd, J = 17.6, 7.2 Hz, 1H), 6.70 (dd, J = 17.6, 7.2 Hz, 1H), 6.53

(dd, J = 16.0, 9.2 Hz, 1H), 6.39 (s, 1H), 6.27 (d, J = 16.0 Hz, 1H), 5.81 (dd, J = 17.6, 0.8 Hz, 1H), 5.72 (dd, J = 17.6, 0.8 Hz, 1H), 5.30 (dd, J = 11.2, 0.8 Hz, 1H), 5.24 (dd, J = 11.2, 0.8 Hz, 1H), 4.53 (d, J = 13.2 Hz, 1H), 4.41 (dd, J = 13.2, 2.4 Hz, 1H), 4.28 (dd, J = 9.2, 1.2 Hz, 1H), 3.95 (dd, J = 9.2, 6.6 Hz, 1H), 3.81-3.83 (m, 2H). **^{13}C NMR** ($CDCl_3$, 100 MHz): δ 143.5, 142.4, 137.4, 136.7, 136.43, 136.41, 136.0, 135.8, 132.3, 128.5, 128.4, 128.2, 127.8, 127.2, 126.3, 126.2, 120.6, 113.8, 113.5, 73.3, 71.4, 48.7, 47.0. **HRMS** (ESI) *m*/*z* for $C_{30}H_{28}OK$ $[M+K]^+$ calcd 443.1772, found 443.1664.

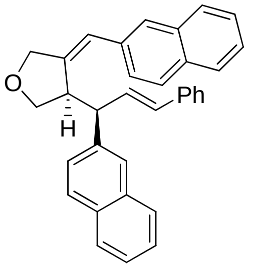

3di

A white solid; **^{1}H NMR** ($CDCl_3$, 400 MHz): δ 7.61-7.86 (m, 8H), 7.26-7.53 (m, 11H), 6.67 (dd, J = 15.6 Hz, 9.2 Hz, 1H), 6.59 (s, 1H), 6.33 (d, J = 15.6 Hz, 1H), 4.65 (d, J = 13.2 Hz, 1H), 4.51 (dd, J = 13.2 Hz, 2.0 Hz, 1H), 4.40 (d, J = 7.6 Hz, 1H), 3.99-4.07 (m, 3H). **^{13}C NMR** ($CDCl_3$, 100 MHz): δ 143.9, 140.3, 137.4, 134.7, 133.4, 133.3, 132.4, 132.2, 132.1, 128.6, 128.5, 128.0, 127.9, 127.8, 127.62, 127.60, 127.4, 127.3, 126.7, 126.3, 126.26, 126.22, 126.15, 126.10, 125.9, 125.8, 125.5, 121.0, 73.3, 71.5, 49.3, 47.2. **HRMS** (ESI) *m*/*z* for $C_{34}H_{32}NO$ $[M+NH_4]^+$ calcd 470.2478, found 470.2475.

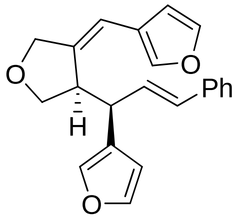

3dj

A white solid; **^{1}H NMR** ($CDCl_3$, 400 MHz): δ 7.41-7.46 (m, 3H), 7.28-7.36 (m, 5H), 7.21-7.24 (m, 1H), 6.51 (d, J = 1.2 Hz, 1H), 6.34-6.36 (m, 3H), 6.21 (d, J = 2.0 Hz, 1H), 4.39 (dt, J = 13.2, 1.6 Hz, 1H), 4.32 (dd, J = 13.2, 2.0 Hz, 1H), 4.21 (dd, J = 9.2, 2.0 Hz, 1H), 3.93 (dd, J = 9.2, 6.4 Hz, 1H), 3.80 (dd, J = 8.0, 4.0 Hz, 1H), 3.50-3.52 (m, 1H). **^{13}C NMR** ($CDCl_3$, 100 MHz): δ 143.5, 143.3, 142.2, 140.7, 139.1, 137.4, 132.0, 128.5, 128.0, 127.3, 126.9, 126.3, 122.4, 110.5, 110.0, 109.6, 72.7, 71.0, 46.8, 41.0. **HRMS** (ESI) *m*/*z* for $C_{22}H_{21}O$ $[M+H]^+$ calcd 333.1485, found 333.1491.

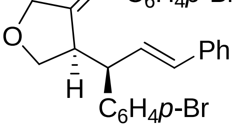

3dk

A white solid; **^{1}H NMR** ($CDCl_3$, 400 MHz): δ 7.45 (d, J = 8.4 Hz, 2H), 7.24-7.34 (m, 7H), 7.04 (d, J = 8.4 Hz, 2H), 6.96 (d, J = 8.4 Hz, 2H), 6.42 (dd, J = 15.6, 9.2 Hz, 1H), 6.29 (d, J = 15.6 Hz, 1H), 6.29 (d, J = 1.6 Hz, 1H), 4.53 (dt, J = 13.2, 2.0 Hz, 1H), 4.38 (dd, J = 13.2, 1.6

Hz, 1H), 4.23 (dd, J = 8.8, 1.6 Hz, 1H), 3.93 (dd, J = 8.8, 5.6 Hz, 1H), 3.68-3.71 (m, 1H), 3.61 (dd, J = 9.2, 6.4 Hz, 1H). **^{13}C NMR** ($CDCl_3$, 100 MHz): δ 143.8, 141.3, 137.0, 136.1, 132.6, 131.5, 131.3, 129.4, 129.3, 128.5, 128.3, 127.5, 126.3, 120.5, 120.3, 72.7, 71.5, 49.0, 46.6. **HRMS** (ESI) *m/z* for $C_{26}H_{23}Br_2O$ $[M+H]^+$ calcd 509.0110, found 508.9932.

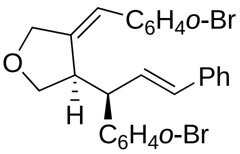

3dl

A colorless oil; **^{1}H NMR** ($CDCl_3$, 400 MHz): δ 7.45-7.49 (m, 2H), 7.36-7.39 (m, 2H), 7.23-7.33 (m, 5H), 7.04-7.10 (m, 2H), 6.91-6.94 (m, 2H), 6.42-6.46 (m, 2H), 6.39 (d, J = 1.6 Hz, 1H), 4.73 (dt, J = 13.2, 2.0 Hz, 1H), 4.44 (dd, J = 13.2, 2.0 Hz, 1H), 4.24 (dd, J = 9.2, 2.0 Hz, 1H), 4.07-4.10 (m, 1H), 3.88 (dd, J = 9.2, 5.6 Hz, 1H), 3.66-3.68 (m, 1H). **^{13}C NMR** ($CDCl_3$, 100 MHz): δ 144.1, 141.1, 137.8, 137.2, 133.1, 132.8, 132.6, 130.2, 129.0, 128.5, 128.4, 128.1, 127.6, 127.4, 127.2, 127.0, 126.3, 124.3, 124.0, 121.4, 72.4, 71.4, 48.8, 44.6. **HRMS** (ESI) *m/z* for $C_{26}H_{23}Br_2O$ $[M+H]^+$ calcd 509.0110, found 508.9931.

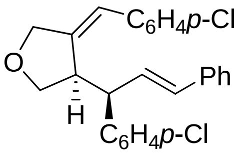

3dm

A white solid; **^{1}H NMR** ($CDCl_3$, 400 MHz): δ 7.25-7.34 (m, 7H), 7.11-7.17 (m, 4H), 7.03 (d, J = 8.4 Hz, 2H), 6.43 (dd, J = 16.0, 9.0 Hz, 1H), 6.32 (d, J = 1.6 Hz, 1H), 6.29 (d, J = 16.0 Hz, 1H), 4.54 (dt, J = 13.2, 1.6 Hz, 1H), 4.40 (dd, J = 13.2, 2.0 Hz, 1H), 4.24 (dd, J = 9.2, 2.0 Hz, 1H), 3.93 (dd, J = 9.2, 6.4 Hz, 1H), 3.70-3.73 (m, 1H), 3.64 (dd, J = 9.0, 6.0 Hz, 1H). **^{13}C NMR** ($CDCl_3$, 100 MHz): δ 143.7, 140.8, 137.0, 135.7, 132.6, 132.4, 129.1, 128.9, 128.52, 128.51, 128.4, 128.3, 127.5, 126.3, 120.2, 72.8, 71.5, 48.8, 46.7. **HRMS** (ESI) *m/z* for $C_{26}H_{22}Cl_2NaO_3$ $[M+Na]^+$ calcd 443.0940, found 443.0941.

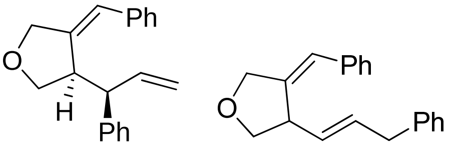

3ea **4ea**

Compound **3ea**: **^{1}H NMR** ($CDCl_3$, 400 MHz): δ 7.08-7.38 (m, 10H), 6.44 (d, J = 2.0 Hz, 1H), 6.17 (ddd, J = 17.6, 10.0, 9.2 Hz, 1H), 5.14 (dd, J = 10.0, 2.4 Hz, 1H), 4.93 (ddd, J = 17.6, 2.4, 0.8 Hz, 1H), 4.56 (dt, J = 12.8, 2.0 Hz, 1H), 4.42 (dd, J = 12.8, 1.6 Hz, 1H), 4.23 (dd, J = 9.2, 2.4 Hz, 1H), 3.90-3.94 (m, 2H), 3.64 (dd, J = 9.2, 4.8 Hz, 1H). Compound **4ea**: **^{1}H NMR** ($CDCl_3$, 400 MHz): δ 7.08-7.38 (m, 10H), 6.47 (d, J = 2.0 Hz, 1H), 5.73 (dtd, J = 15.2, 7.2, 0.8 Hz, 1H), 5.61 (ddt, J = 15.2, 6.4, 1.2 Hz, 1H), 4.62 (dt, J = 13.2, 1.6 Hz, 1H), 4.46 (dd, J = 13.2, 1.6 Hz, 1H), 4.02 (dd, J = 8.4, 6.0 Hz, 1H), 3.72-3.75 (m, 2H), 3.36 (d, J = 7.2 Hz, 2H). Mixture of **3ea** and **4ea**: **^{13}C NMR** ($CDCl_3$, 100 MHz): δ 143.5, 142.8, 142.5, 137.2, 136.7, 136.6, 130.8, 130.4, 128.44, 128.42, 128.40, 128.36, 128.30, 128.2, 128.0, 127.6, 126.7, 126.6, 126.3, 125.9, 121.5, 120.8, 117.2, 75.4, 73.3, 73.0, 71.2, 49.6, 46.8, 44.8, 38.71. **HRMS** (ESI) *m/z* for $C_{20}H_{21}O$ $[M+H]^+$ calcd 277.1592, found 277.1591.

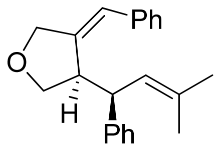

3fa

A colorless oil; **^{1}H NMR** (CDCl$_3$, 400 MHz): δ 7.20-7.35 (m, 8H), 7.12-7.15 (m, 2H), 6.36 (d, J = 2.0 Hz, 1H), 5.55 (dqd, J = 10.0, 1.2, 1.2 Hz, 1H), 4.54 (dt, J = 12.8, 2.0 Hz, 1H), 4.40 (dd, J = 12.8, 1.6 Hz, 1H), 4.15 (dd, J = 8.8, 2.0 Hz, 1H), 3.90 (dd, J = 10.0, 5.2 Hz, 2H), 3.86 (dd, J = 8.8, 6.4 Hz, 1H), 3.67-3.71 (m, 3H), 1.75 (d, J = 1.2 Hz, 3H), 1.34 (d, J = 1.2 Hz, 3H). **^{13}C NMR** (CDCl$_3$, 100 MHz): δ 143.9, 143.8, 137.3, 134.2, 128.3, 128.2, 128.0, 127.6, 126.6, 126.0, 123.0, 120.7, 73.3, 71.5, 47.1, 43.4, 26.1, 18.0. **HRMS** (ESI) *m/z* for $C_{22}H_{25}O$ $[M+H]^+$ calcd 305.1900, found 305.1903.

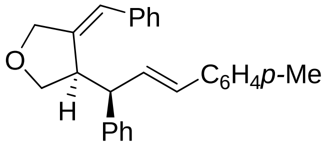

3ga

A colorless oil; **^{1}H NMR** (CDCl$_3$, 400 MHz): δ 7.35-7.39 (m, 4H), 7.25-7.28 (m, 5H), 7.18-7.21 (m, 3H), 7.12 (d, J = 6.4 Hz, 2H), 6.49 (dd, J = 12.4, 7.2 Hz, 1H), 6.41 (d, J = 0.8 Hz, 1H), 6.22 (d, J = 12.4 Hz, 1H), 4.50 (dt, J = 13.2, 2.0 Hz, 1H), 4.40 (dd, J = 13.2, 1.2 Hz, 1H), 4.26 (dd, J = 9.0, 1.6 Hz, 1H), 3.93 (dd, J = 9.0, 6.4 Hz, 1H), 3.78-3.81 (m, 2H), 2.35 (s, 3H). **^{13}C NMR** (CDCl$_3$, 100 MHz): δ 143.6, 143.0, 137.3, 137.0, 134.7, 132.1, 129.2, 128.5, 128.4, 128.0, 127.6, 127.3, 126.7, 126.4, 126.2, 120.8, 73.3, 71.3, 48.7, 47.1, 21.1. **HRMS** (ESI) *m/z* for $C_{27}H_{27}O$ $[M+H]^+$ calcd 367.2056, found 367.2060.

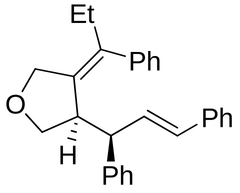

3ha

A white solid; **^{1}H NMR** (CDCl$_3$, 400 MHz): δ 7.40-7.44 (m, 4H), 7.32-7.36 (m, 3H), 7.12-7.28 (m, 6H), 6.83-6.85 (m, 2H), 6.55 (dd, J = 15.6, 9.6 Hz, 1H), 6.37 (d, J = 15.6 Hz, 1H), 4.46 (d, J = 1.6 Hz, 2H), 4.11 (dd, J = 9.2, 1.6 Hz, 1H), 3.79 (dd, J = 9.2, 6.0 Hz, 1H), 3.37 (dd, J = 9.6, 4.8 Hz, 1H), 3.29-3.32 (m, 1H), 2.22-2.27 (m, 2H), 0.93 (t, J = 7.6 Hz, 3H). **^{13}C NMR** (CDCl$_3$, 100 MHz): δ 143.4, 142.1, 137.7, 137.0, 135.3, 131.8, 128.5, 128.4, 128.3, 128.2, 127.9, 127.5, 127.1, 126.7, 126.3, 126.0, 70.5, 70.1, 49.8, 48.4, 28.8, 12.4. **HRMS** (ESI) *m/z* for $C_{28}H_{29}O$ $[M+H]^+$ calcd 381.2213, found 381.2206.

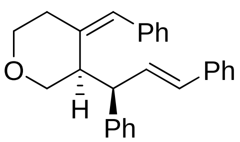

3ia

A white solid; **^{1}H NMR** (CDCl$_3$, 400 MHz): δ 7.19-7.34 (m, 9H), 7.11-7.14 (m, 2H), 6.85-6.91 (m, 4H), 6.54 (d, J = 15.6 Hz, 1H), 6.19 (d, J = 1.6 Hz, 1H), 6.18 (dd, J = 15.6, 8.0

Hz, 1H), 4.33 (d, J = 11.2 Hz, 1H), 4.26 (dd, J = 11.2, 5.6 Hz, 1H), 4.02-4.07 (m, 1H), 3.55-3.66 (m, 2H), 3.23-3.26 (m, 1H), 2.76-2.78 (m, 1H), 1.96-1.99 (m, 1H). **^{13}C NMR** ($CDCl_3$, 100 MHz): δ 142.2, 138.7, 138.5, 137.5, 137.2, 132.3, 131.4, 129.1, 128.4, 128.2, 127.9, 127.1, 126.3, 126.2, 126.1, 125.8, 70.6, 70.5, 49.3, 42.6, 34.4. **HRMS** (ESI) *m*/*z* for $C_{27}H_{30}NO$ $[M+NH_4]^+$ calcd 384.2322, found 384.2325.

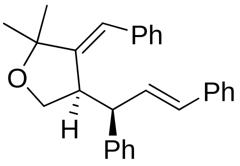

3ja

A colorless oil; **^{1}H NMR** ($CDCl_3$, 400 MHz): δ 7.24-7.42 (m, 13H), 7.15-7.19 (m, 2H), 6.52 (dd, J = 16.0, 10.0 Hz, 1H), 6.31 (d, J = 1.6 Hz, 1H), 6.14 (d, J = 16.0 Hz, 1H), 4.16-4.20 (m, 1H), 3.93-3.98 (m, 2H), 3.74 (dd, J = 9.6, 3.6 Hz, 1H), 1.41 (s, 3H), 1.35 (s, 3H). **^{13}C NMR** ($CDCl_3$, 100 MHz): δ 151.5, 143.5, 137.6, 132.2, 128.8, 128.5, 128.4, 128.3, 128.2, 127.6, 127.1, 126.7, 126.3, 126.2, 121.0, 83.6, 66.4, 48.1, 48.0, 28.2, 27.1. **HRMS** (ESI) *m*/*z* for $C_{28}H_{29}O$ $[M+H]^+$ calcd 381.2213, found 381.2222.

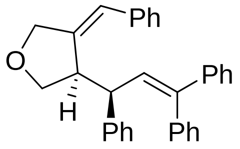

3ka

A white solid; **^{1}H NMR** ($CDCl_3$, 400 MHz): δ 7.26-7.30 (m, 7H), 7.08-7.20 (m, 9H), 7.00-7.02 (m, 2H), 6.85-6.87 (m, 2H), 6.60 (d, J = 10.8 Hz, 1H), 6.31 (d, J = 1.6 Hz, 1H), 4.66 (dt, J = 13.2, 1.6 Hz, 1H), 4.45 (dd, J = 13.2, 1.6 Hz, 1H), 4.35 (dd, J = 9.2, 1.2 Hz, 1H), 3.97 (dd, J = 10.8, 4.0 Hz, 1H), 3.87 (dd, J = 9.2, 6.0 Hz, 1H), 3.62-3.64 (m, 1H),). **^{13}C NMR** ($CDCl_3$, 100 MHz): δ 143.3, 143.3, 142.7, 139.4, 136.9, 129.6, 128.5, 128.2, 128.1, 127.9, 127.8, 127.6, 127.5, 127.2, 127.1, 126.7, 126.4, 126.2, 121.1, 73.2, 71.2, 47.6, 44.8. **HRMS** (ESI) *m*/*z* for $C_{32}H_{29}O$ $[M+H]^+$ calcd 429.2213, found 429.2219.

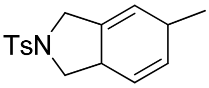

3a

This is a known compound; A white solid; **^{1}H NMR** ($CDCl_3$, 400 MHz): δ 7.70 (d, J = 8.4 Hz, 2H), 7.31 (d, J = 8.4 Hz, 2H), 5.60 (d, J = 0.8 Hz, 2H), 5.37 (s, 1H), 3.98-4.02 (m, 1H), 3.83 (t, J = 8.4 Hz, 1H), 3.72 (dd, J = 13.2, 2.0 Hz, 1H), 2.88-2.96 (m, 1H), 2.73-2.76 (m, 1H), 2.65 (dd, J = 10.2, 8.8 Hz, 1H), 2.42 (s, 3H), 1.03 (d, J = 7.6 Hz, 3H).

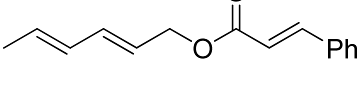

3ba

This is a known compound; A colorless oil; **^{1}H NMR** ($CDCl_3$, 400 MHz): δ 7.71 (d, J = 16.0 Hz, 1H), 7.54-7.56 (m, 2H), 7.40-7.42 (m, 3H), 6.47 (d, J = 16.0 Hz, 1H), 6.33 (dd, J = 15.6, 10.8 Hz, 1H), 6.07-6.14 (m, 1H), 5.71-5.83 (m, 2H), 4.74 (d, J = 6.8 Hz, 2H), 1.80 (d, J = 6.8 Hz, 3H).

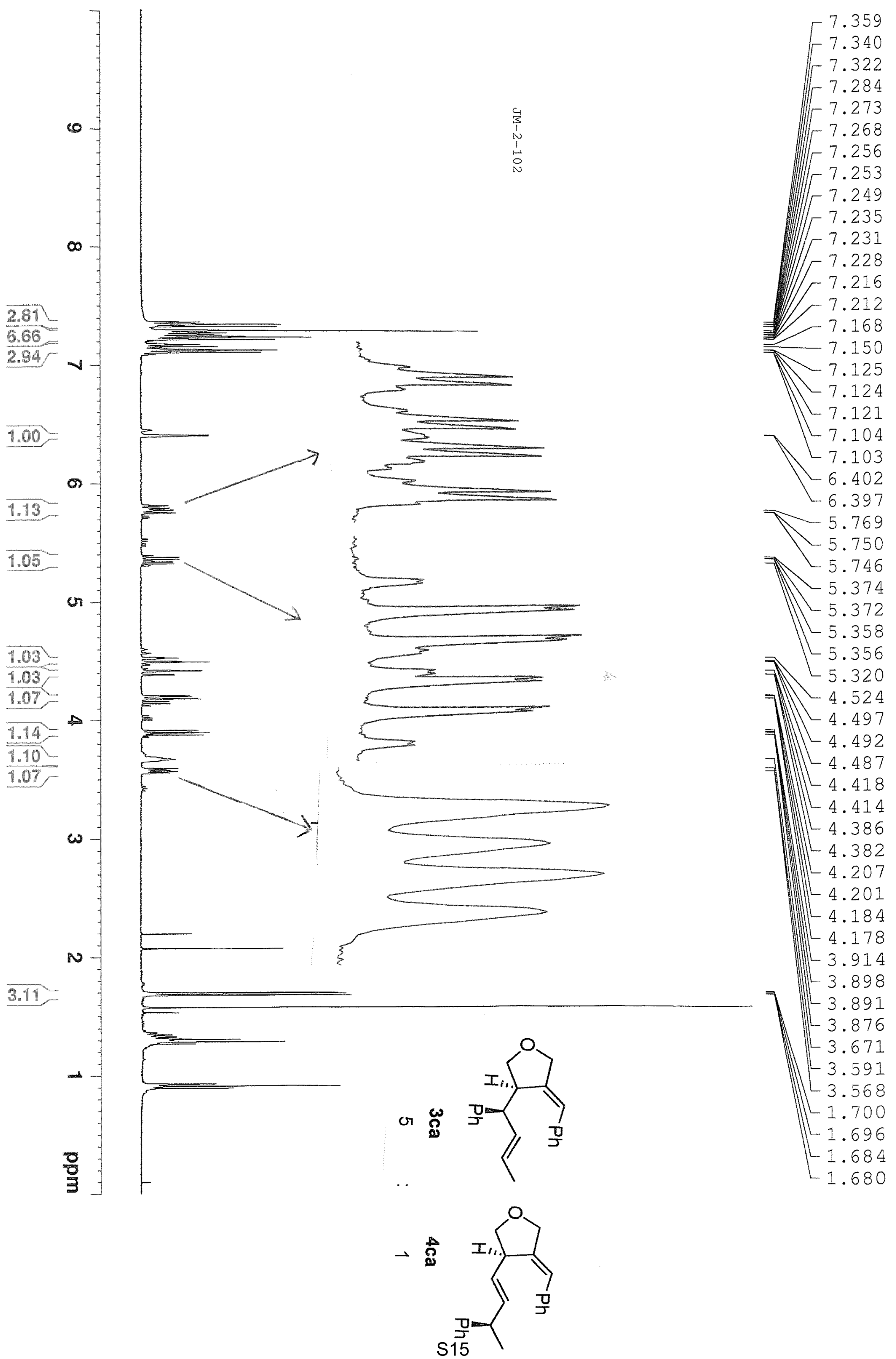

JM-2-102
7.359
7.340
7.322
7.284
7.273
7.268
7.256
7.253
7.249
7.235
7.231
7.228
7.216
7.212
7.168
7.150
7.125
7.124
7.121
7.104
7.103
6.402
6.397
5.769
5.750
5.746
5.374
5.372
5.358
5.356
5.320
4.524
4.497
4.492
4.487
4.418
4.414
4.386
4.382
4.207
4.201
4.184
4.178
3.914
3.898
3.891
3.876
3.671
3.591
3.568
1.700
1.696
1.684
1.680
2.81
6.66
2.94
1.00
1.13
1.05
1.03
1.03
1.07
1.14
1.10
1.07
3.11
9
8
7
6
5
4
3
2
1
ppm
3ca
5
4ca
1
Ph
H

S15

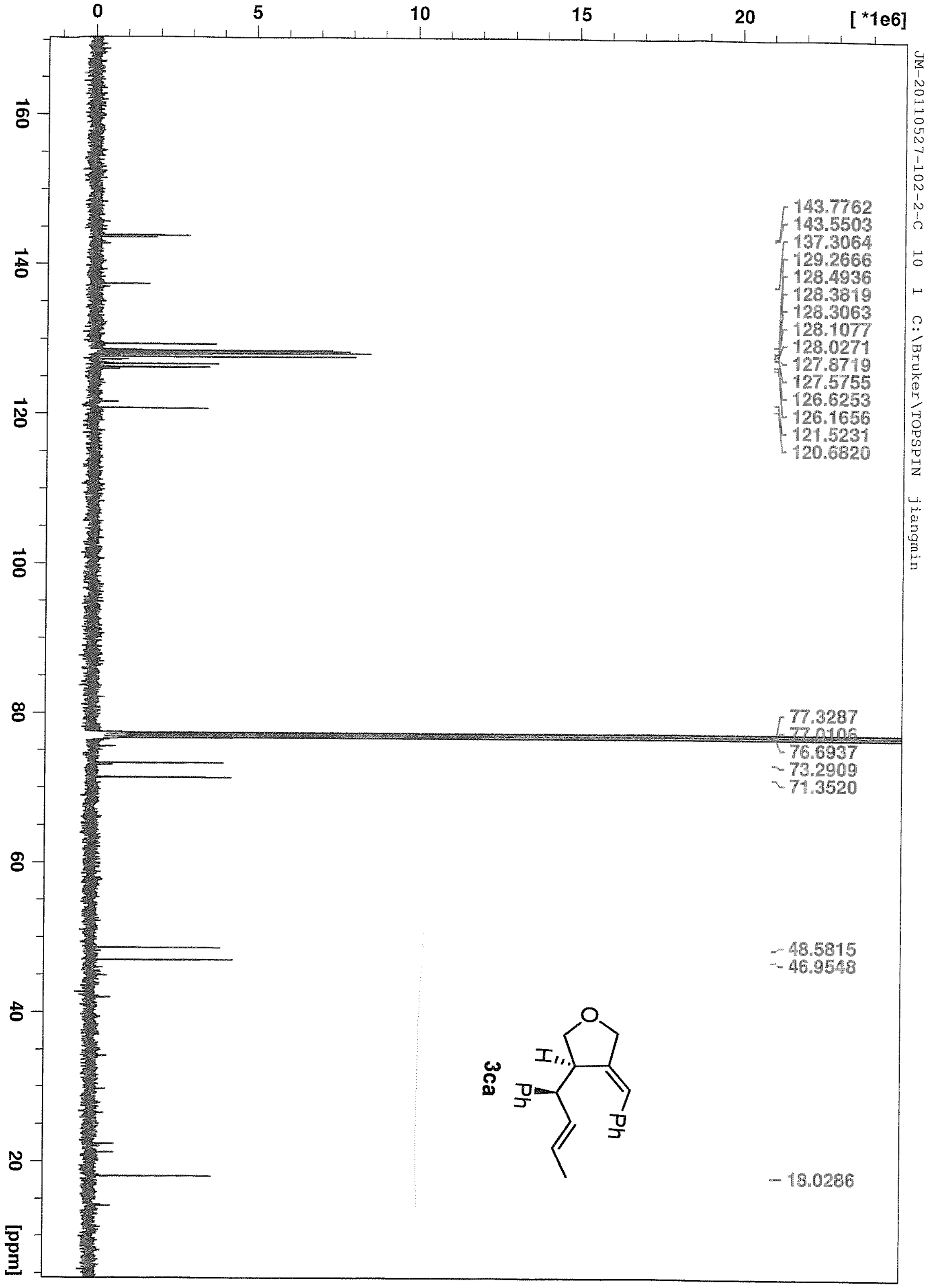
JM-20110527-102-2-C 10 1 C:\Bruker\TOPSPIN jiangmin
143.7762
143.5503
137.3064
129.2666
128.4936
128.3819
128.3063
128.1077
128.0271
127.8719
127.5755
126.6253
126.1656
121.5231
120.6820
77.3287
77.0106
76.6937
73.2909
71.3520
48.5815
46.9548
18.0286
3ca
[ppm]
[*1e6]

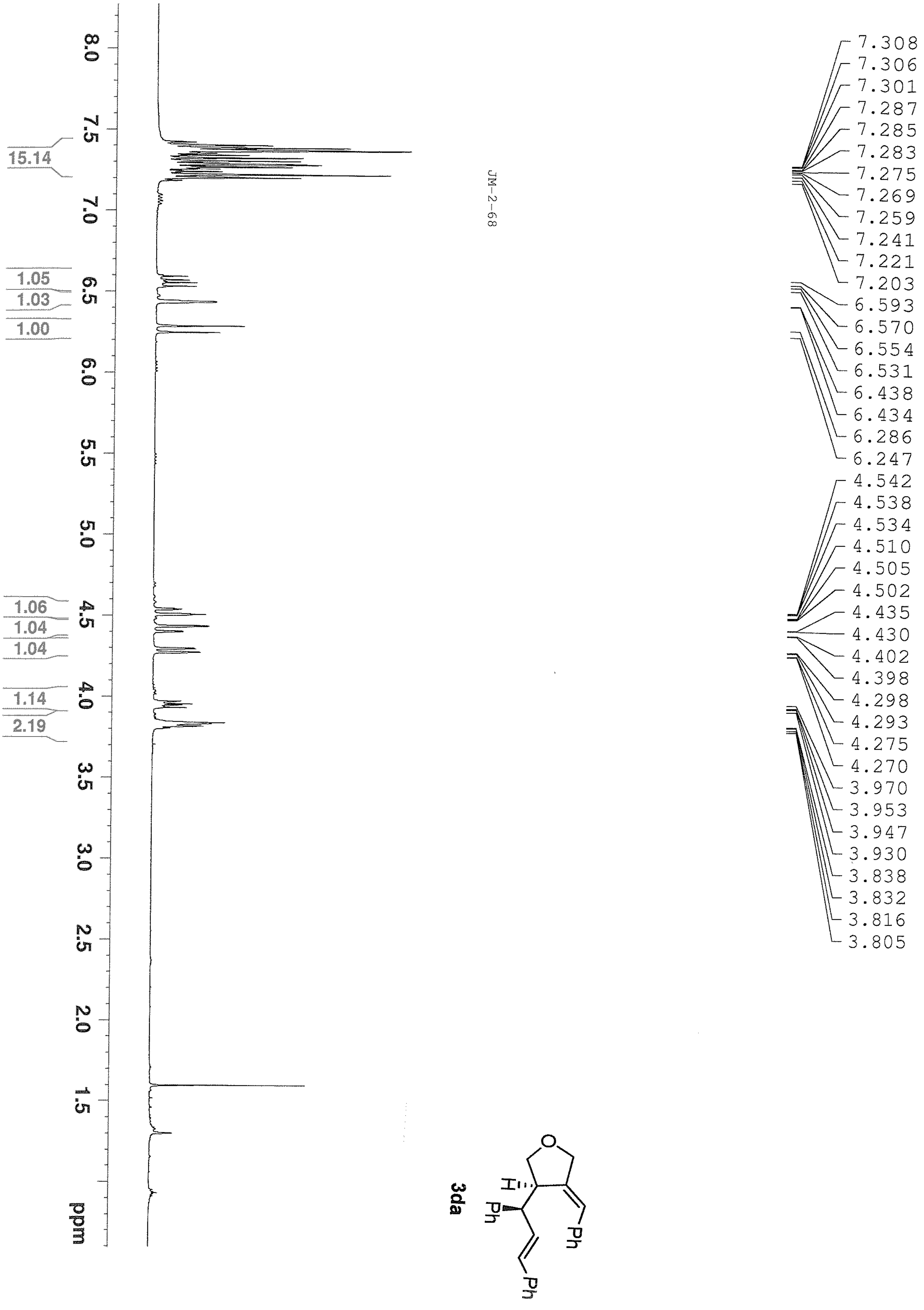
JM-2-68
7.308
7.306
7.301
7.287
7.285
7.283
7.275
7.269
7.259
7.241
7.221
7.203
6.593
6.570
6.554
6.531
6.438
6.434
6.286
6.247
4.542
4.538
4.534
4.510
4.505
4.502
4.435
4.430
4.402
4.398
4.298
4.293
4.275
4.270
3.970
3.953
3.947
3.930
3.838
3.832
3.816
3.805
15.14
1.05
1.03
1.00
1.06
1.04
1.04
1.14
2.19
8.0
7.5
7.0
6.5
6.0
5.5
5.0
4.5
4.0
3.5
3.0
2.5
2.0
1.5
ppm
3da
Ph
Ph
Ph

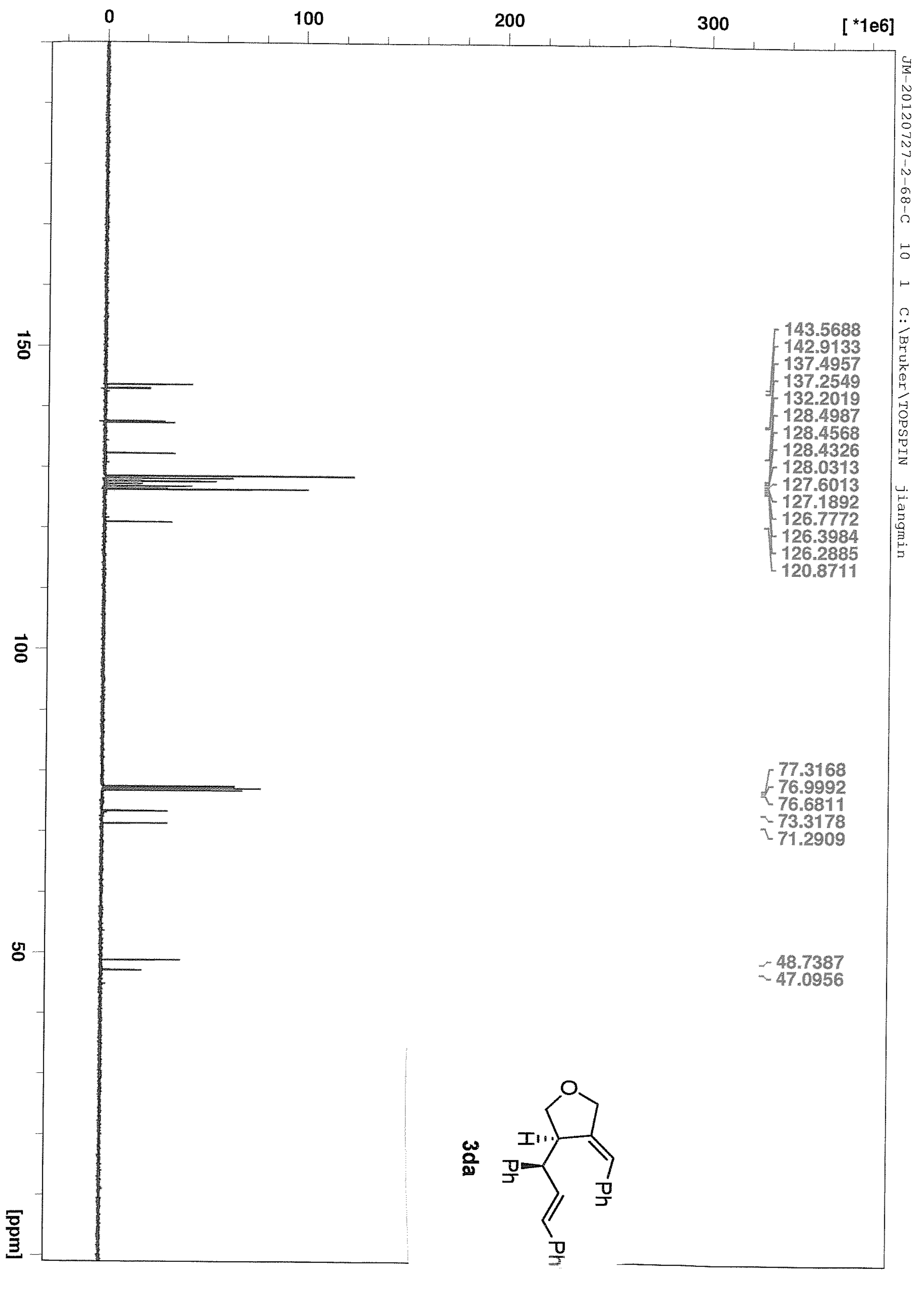
JM-20120727-2-68-C 10 1 C:\Bruker\TOPSPIN jiangmin
143.5688
142.9133
137.4957
137.2549
132.2019
128.4987
128.4568
128.4326
128.0313
127.6013
127.1892
126.7772
126.3984
126.2885
120.8711
77.3168
76.9992
76.6811
73.3178
71.2909
48.7387
47.0956
[ppm]
[*1e6]
3da
Ph
Ph
Ph
H
O

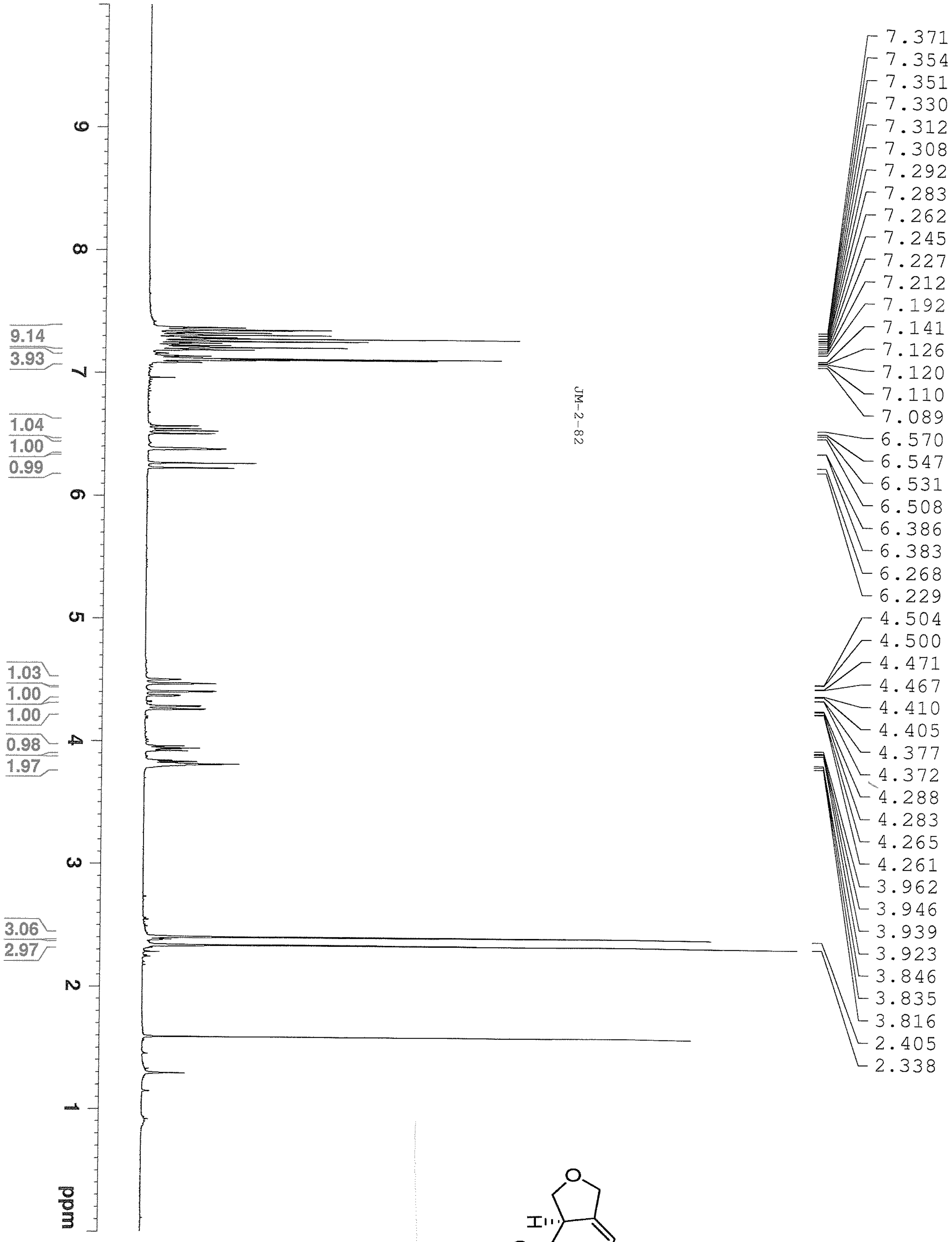
JM-2-82
9
8
7
6
5
4
3
2
1
ppm
9.14
3.93
1.04
1.00
0.99
1.03
1.00
1.00
0.98
1.97
3.06
2.97
7.371
7.354
7.351
7.330
7.312
7.308
7.292
7.283
7.262
7.245
7.227
7.212
7.192
7.141
7.126
7.120
7.110
7.089
6.570
6.547
6.531
6.508
6.386
6.383
6.268
6.229
4.504
4.500
4.471
4.467
4.410
4.405
4.377
4.372
4.288
4.283
4.265
4.261
3.962
3.946
3.939
3.923
3.846
3.835
3.816
2.405
2.338

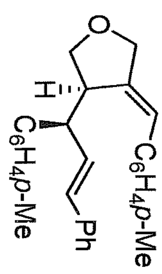
O
H
C_6H_4p-Me
Ph
C_6H_4p-Me

3db

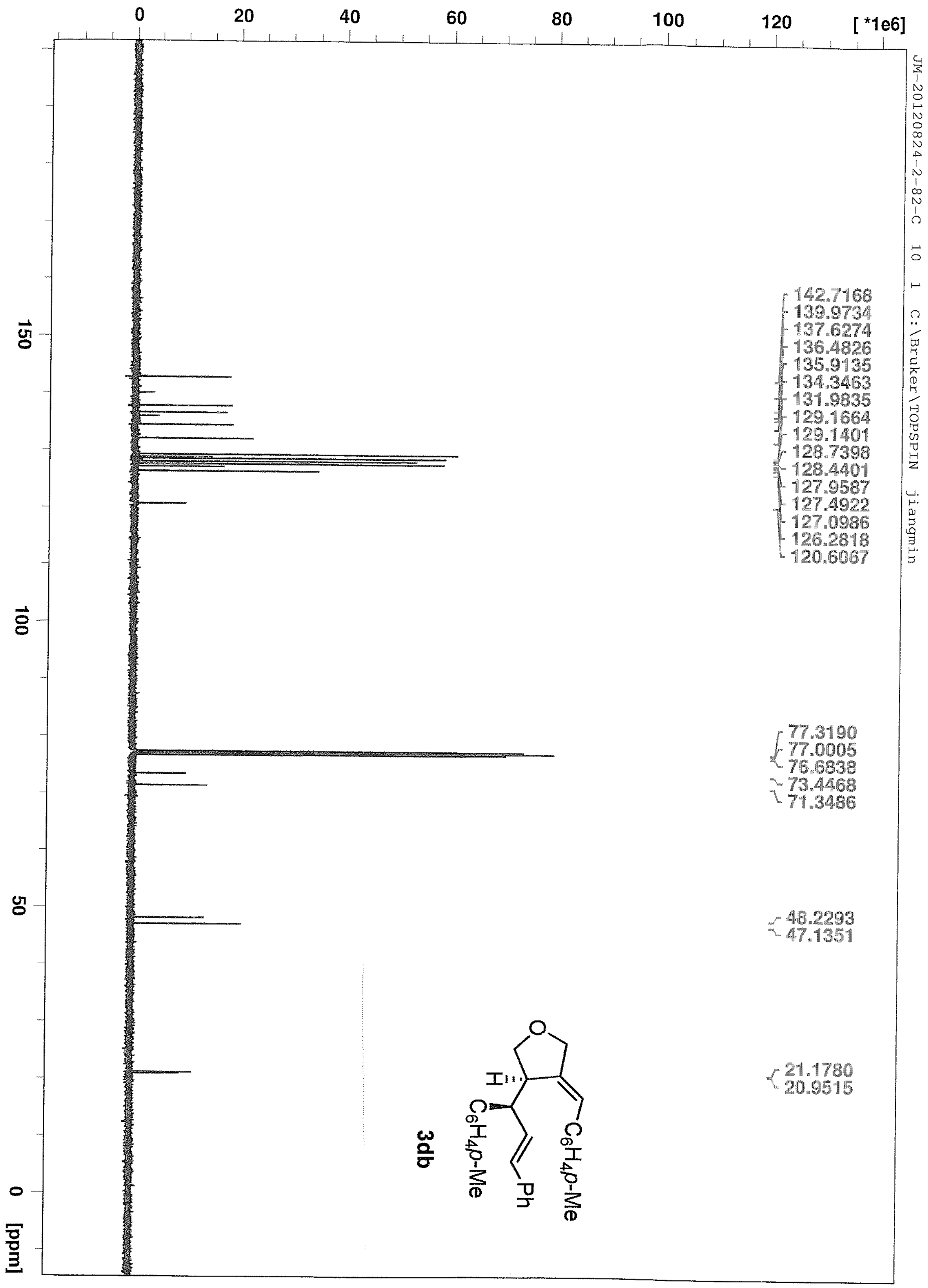
JM-20120824-2-82-C 10 1 C:\Bruker\TOPSPIN jiangmin
142.7168
139.9734
137.6274
136.4826
135.9135
134.3463
131.9835
129.1664
129.1401
128.7398
128.4401
127.9587
127.4922
127.0986
126.2818
120.6067
77.3190
77.0005
76.6838
73.4468
71.3486
48.2293
47.1351
21.1780
20.9515
[ppm]
[*1e6]
3db
C6H4p-Me
Ph

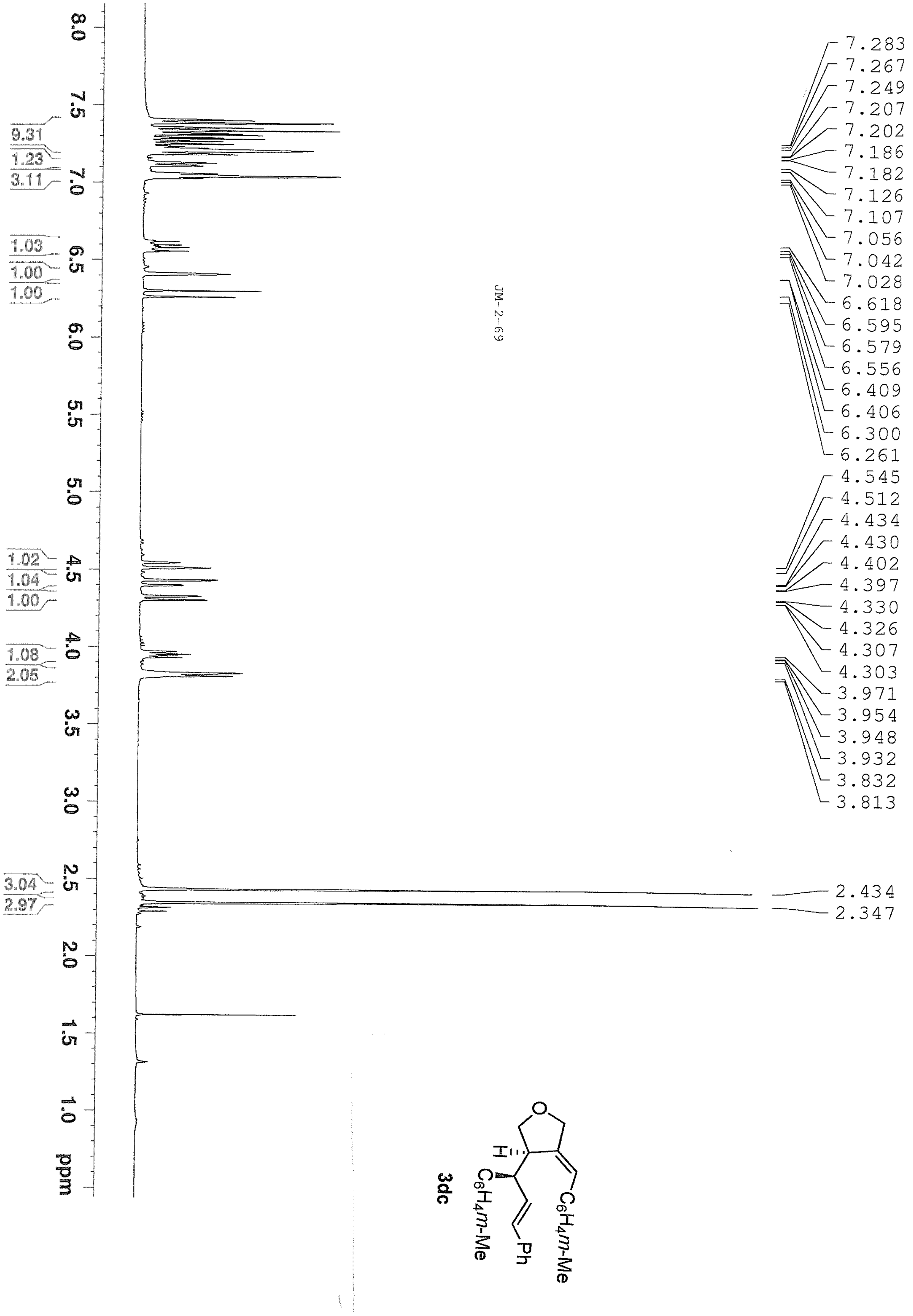
7.283
7.267
7.249
7.207
7.202
7.186
7.182
7.126
7.107
7.056
7.042
7.028
6.618
6.595
6.579
6.556
6.409
6.406
6.300
6.261
4.545
4.512
4.434
4.430
4.402
4.397
4.330
4.326
4.307
4.303
3.971
3.954
3.948
3.932
3.832
3.813
2.434
2.347
9.31
1.23
3.11
1.03
1.00
1.00
1.02
1.04
1.00
1.08
2.05
3.04
2.97
8.0
7.5
7.0
6.5
6.0
5.5
5.0
4.5
4.0
3.5
3.0
2.5
2.0
1.5
1.0
ppm
JM-2-69
O
H
C_6H_4m-Me
Ph
C_6H_4m-Me
3dc

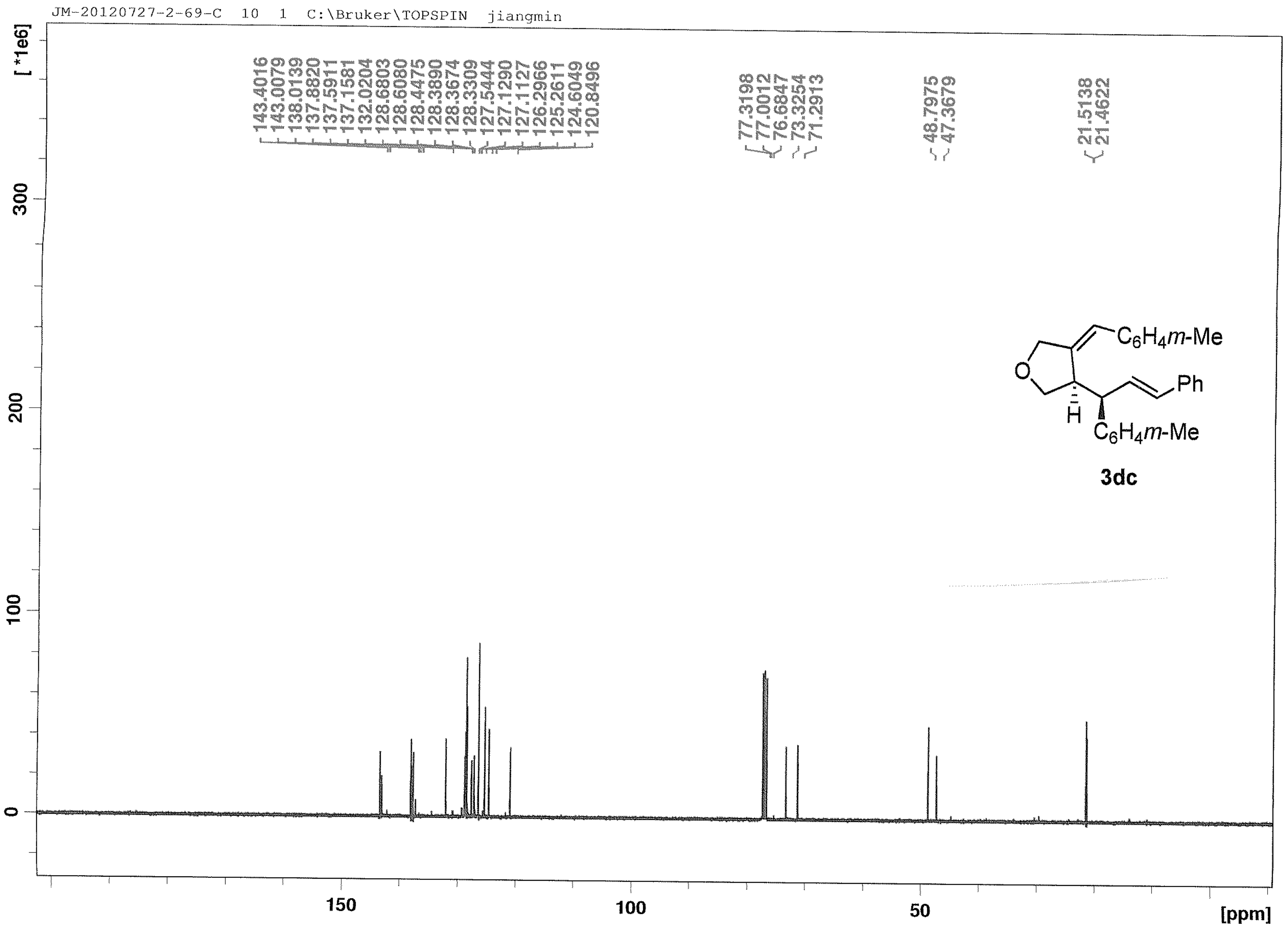
JM-20120727-2-69-C 10 1 C:\Bruker\TOPSPIN jiangmin
143.4016
143.0079
138.0139
137.8820
137.5911
137.1581
132.0204
128.6803
128.6080
128.4475
128.3890
128.3674
128.3309
127.5444
127.1290
127.1127
126.2966
125.2611
124.6049
120.8496
77.3198
77.0012
76.6847
73.3254
71.2913
48.7975
47.3679
21.5138
21.4622
C6H4m-Me
Ph
H
C6H4m-Me
3dc
[*1e6]
300
200
100
0
150
100
50
[ppm]

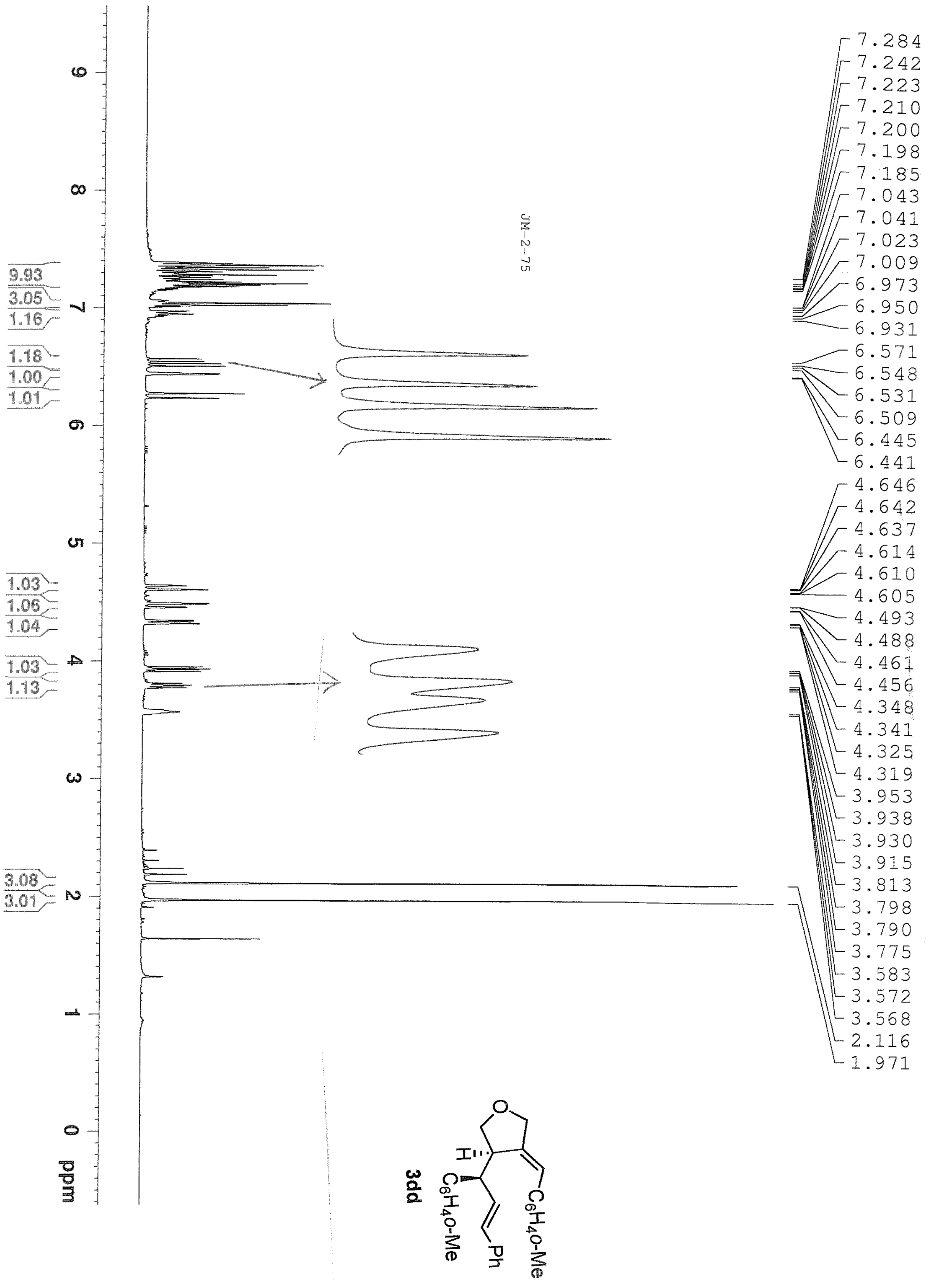
7.284
7.242
7.223
7.210
7.200
7.198
7.185
7.043
7.041
7.023
7.009
6.973
6.950
6.931
6.571
6.548
6.531
6.509
6.445
6.441
4.646
4.642
4.637
4.614
4.610
4.605
4.493
4.488
4.461
4.456
4.348
4.341
4.325
4.319
3.953
3.938
3.930
3.915
3.813
3.798
3.790
3.775
3.583
3.572
3.568
2.116
1.971
JM-2-75
9.93
3.05
1.16
1.18
1.00
1.01
1.03
1.06
1.04
1.03
1.13
3.08
3.01
9
8
7
6
5
4
3
2
1
0
ppm
C6H4o-Me
Ph
C6H4o-Me
3dd

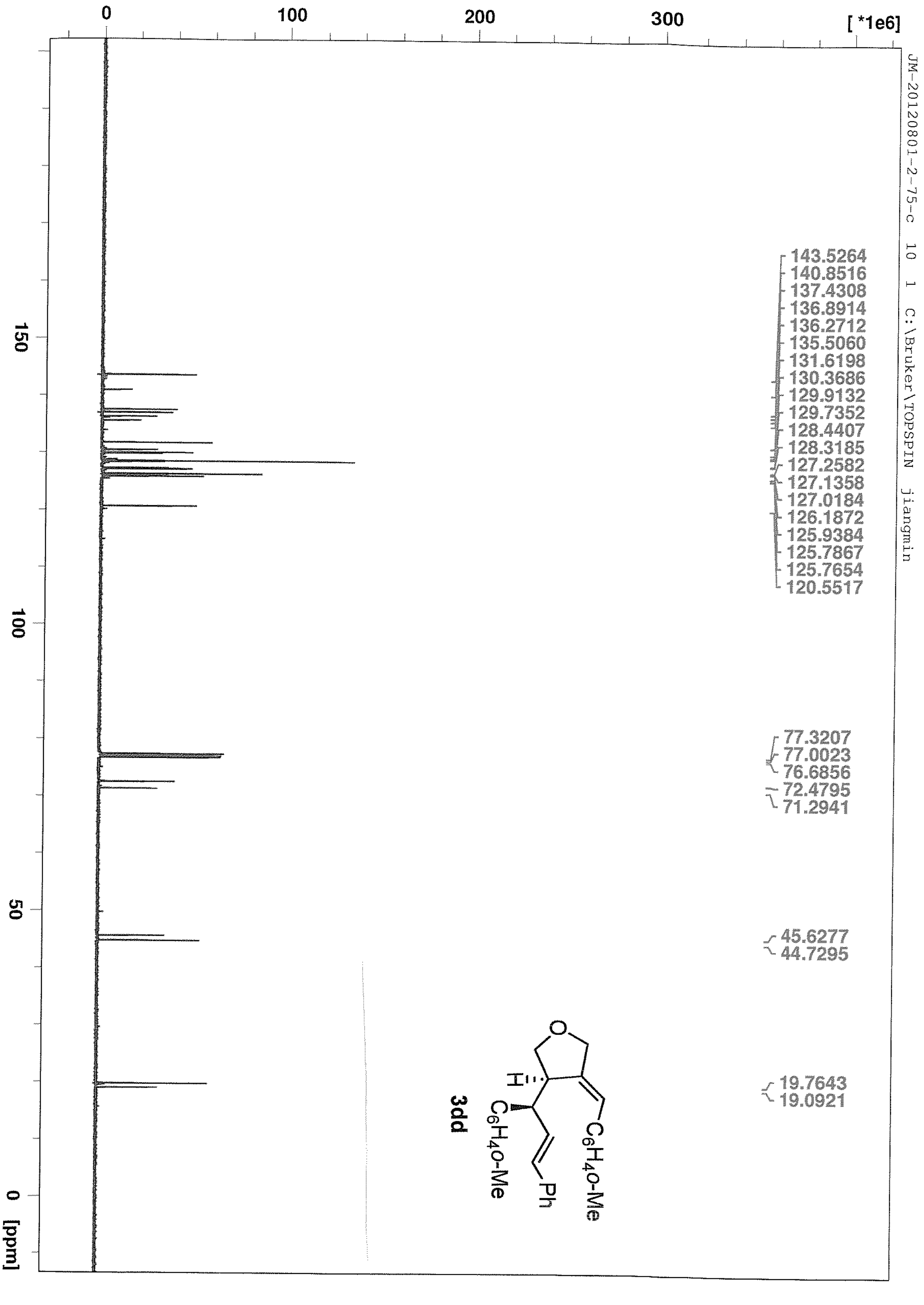
JM-20120801-2-75-c 10 1 C:\Bruker\TOPSPIN jiangmin
143.5264
140.8516
137.4308
136.8914
136.2712
135.5060
131.6198
130.3686
129.9132
129.7352
128.4407
128.3185
127.2582
127.1358
127.0184
126.1872
125.9384
125.7867
125.7654
120.5517
77.3207
77.0023
76.6856
72.4795
71.2941
45.6277
44.7295
19.7643
19.0921
3dd
[*1e6]
[ppm]

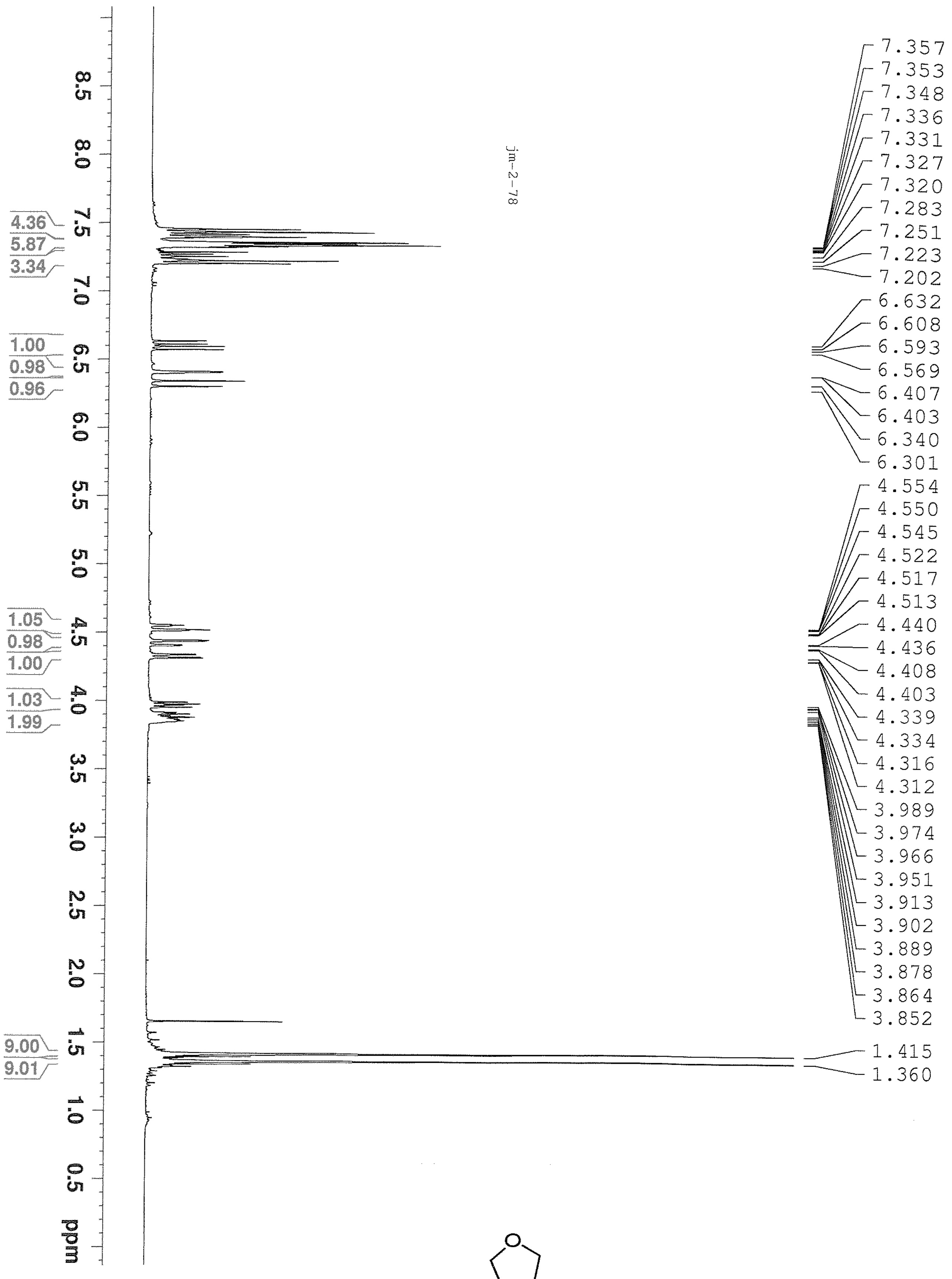
jm-2-78
8.5
8.0
7.5
7.0
6.5
6.0
5.5
5.0
4.5
4.0
3.5
3.0
2.5
2.0
1.5
1.0
0.5
ppm
4.36
5.87
3.34
1.00
0.98
0.96
1.05
0.98
1.00
1.03
1.99
9.00
9.01
7.357
7.353
7.348
7.336
7.331
7.327
7.320
7.283
7.251
7.223
7.202
6.632
6.608
6.593
6.569
6.407
6.403
6.340
6.301
4.554
4.550
4.545
4.522
4.517
4.513
4.440
4.436
4.408
4.403
4.339
4.334
4.316
4.312
3.989
3.974
3.966
3.951
3.913
3.902
3.889
3.878
3.864
3.852
1.415
1.360

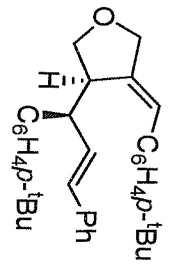
O
H
C_6H_4p-^tBu
Ph
C_6H_4p-^tBu

3de

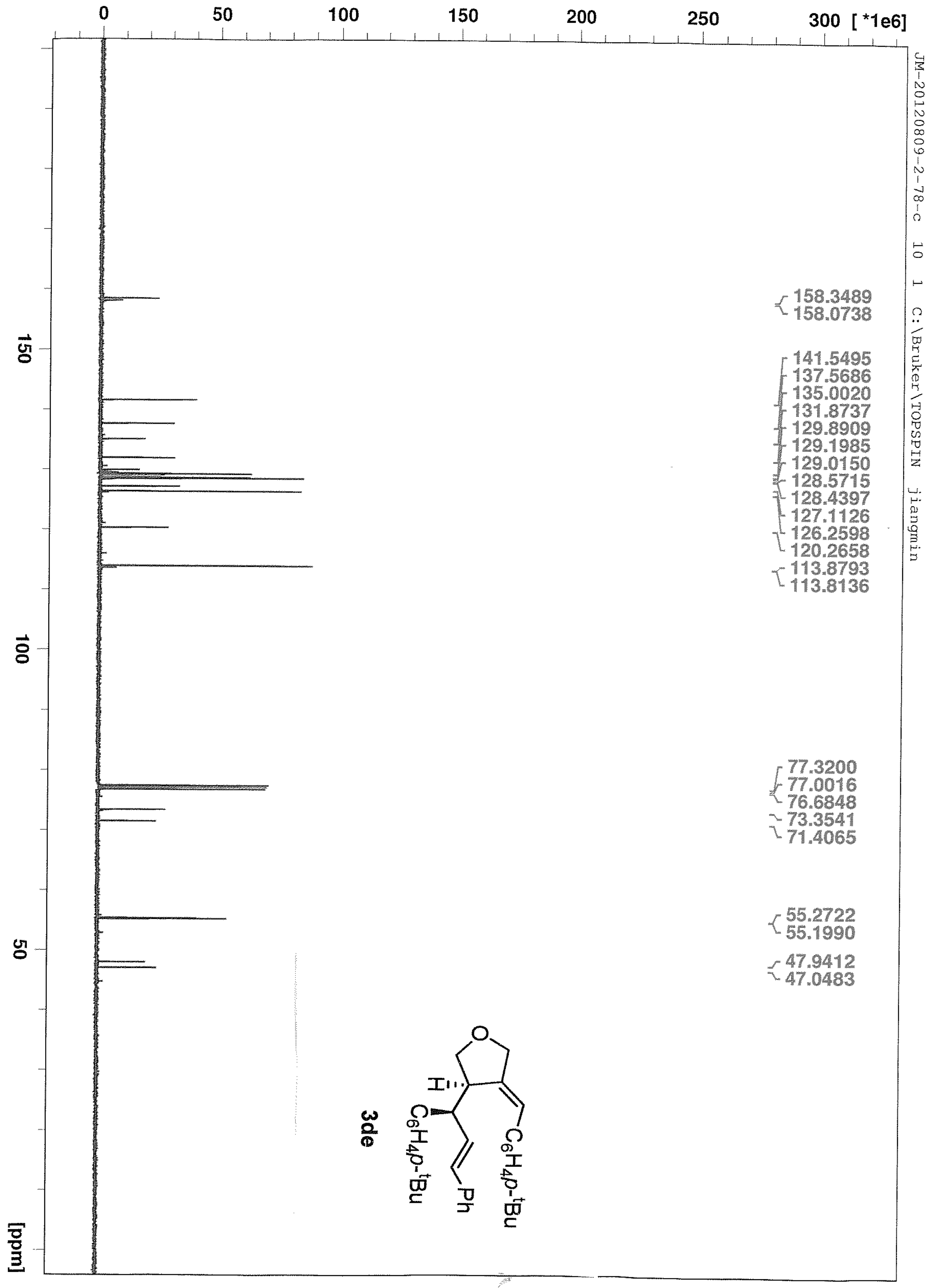
JM-20120809-2-78-c 10 1 C:\Bruker\TOPSPIN jiangmin
[*1e6]
[ppm]
158.3489
158.0738
141.5495
137.5686
135.0020
131.8737
129.8909
129.1985
129.0150
128.5715
128.4397
127.1126
126.2598
120.2658
113.8793
113.8136
77.3200
77.0016
76.6848
73.3541
71.4065
55.2722
55.1990
47.9412
47.0483
3de

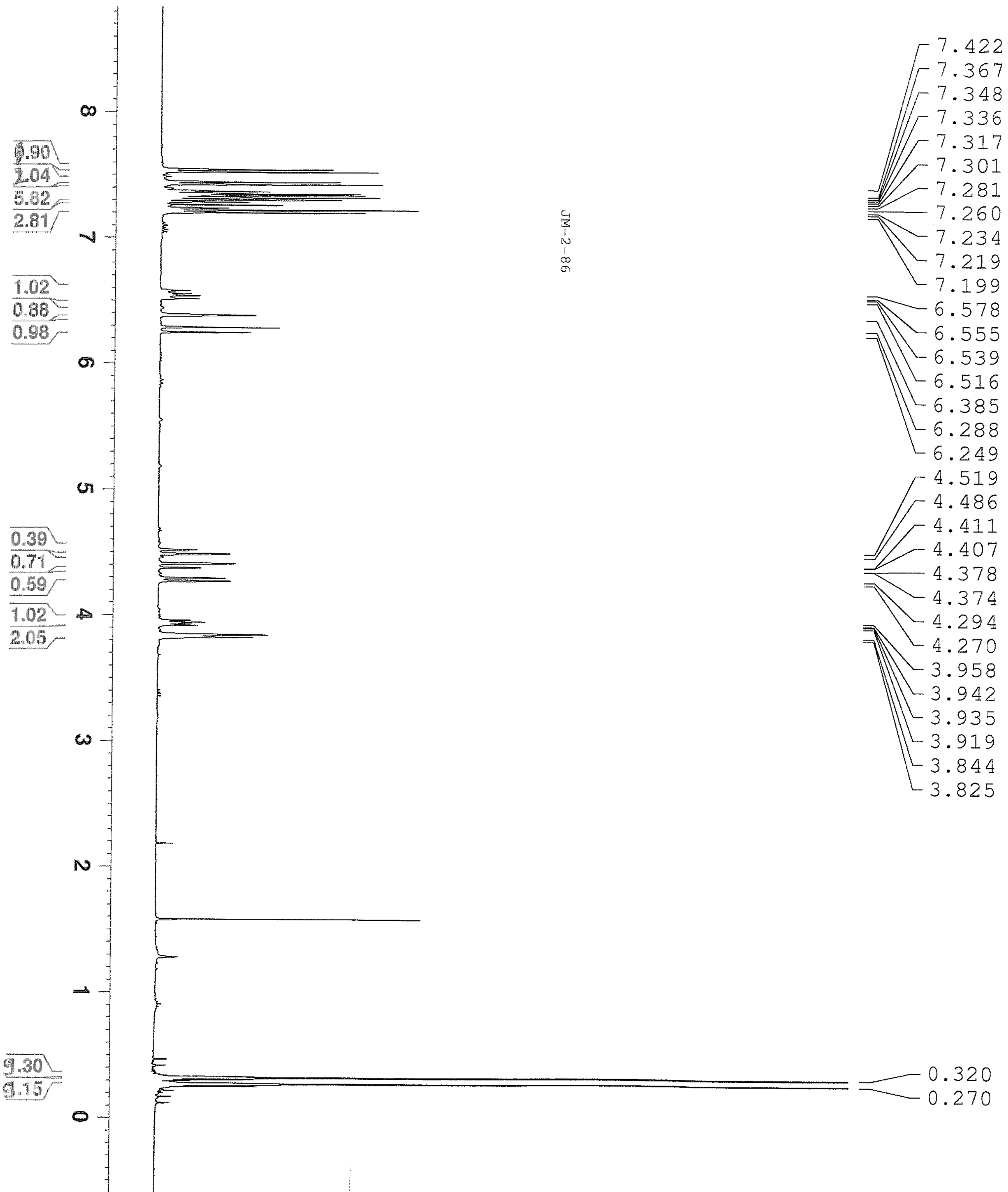
7.422
7.367
7.348
7.336
7.317
7.301
7.281
7.260
7.234
7.219
7.199
6.578
6.555
6.539
6.516
6.385
6.288
6.249
4.519
4.486
4.411
4.407
4.378
4.374
4.294
4.270
3.958
3.942
3.935
3.919
3.844
3.825
0.320
0.270
JM-2-86
5.82
2.81
1.02
0.88
0.98
0.39
0.71
0.59
1.02
2.05
8
7
6
5
4
3
2
1
0
-1
ppm

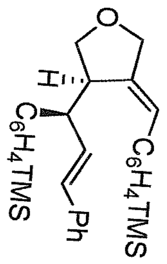
O
H
C_6H_4TMS
Ph
C_6H_4TMS

3df

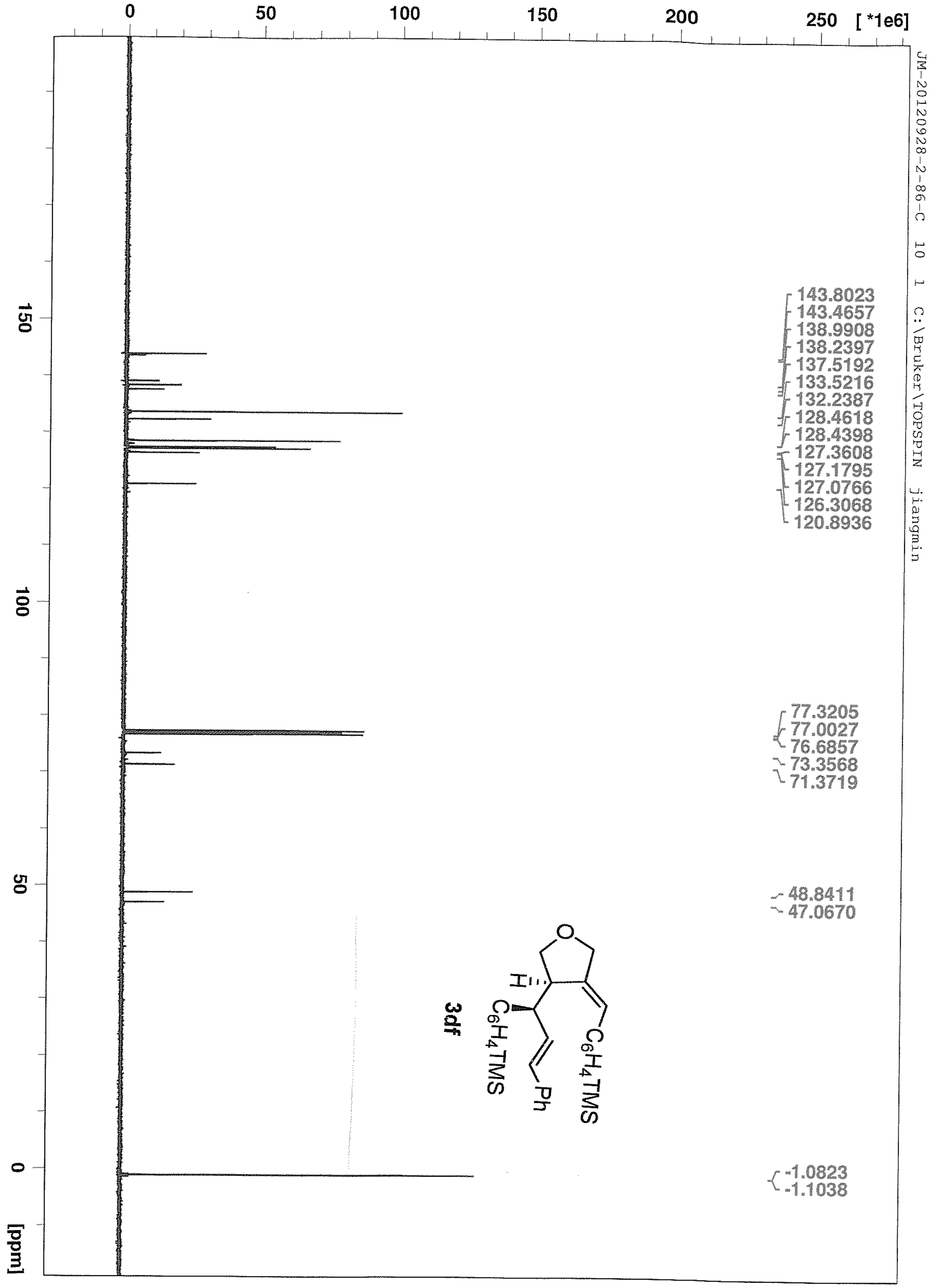
JM-20120928-2-86-C 10 1 C:\Bruker\TOPSPIN jiangmin
143.8023
143.4657
138.9908
138.2397
137.5192
133.5216
132.2387
128.4618
128.4398
127.3608
127.1795
127.0766
126.3068
120.8936
77.3205
77.0027
76.6857
73.3568
71.3719
48.8411
47.0670
-1.0823
-1.1038
C6H4TMS
Ph
C6H4TMS
3df
[ppm]
[*1e6]

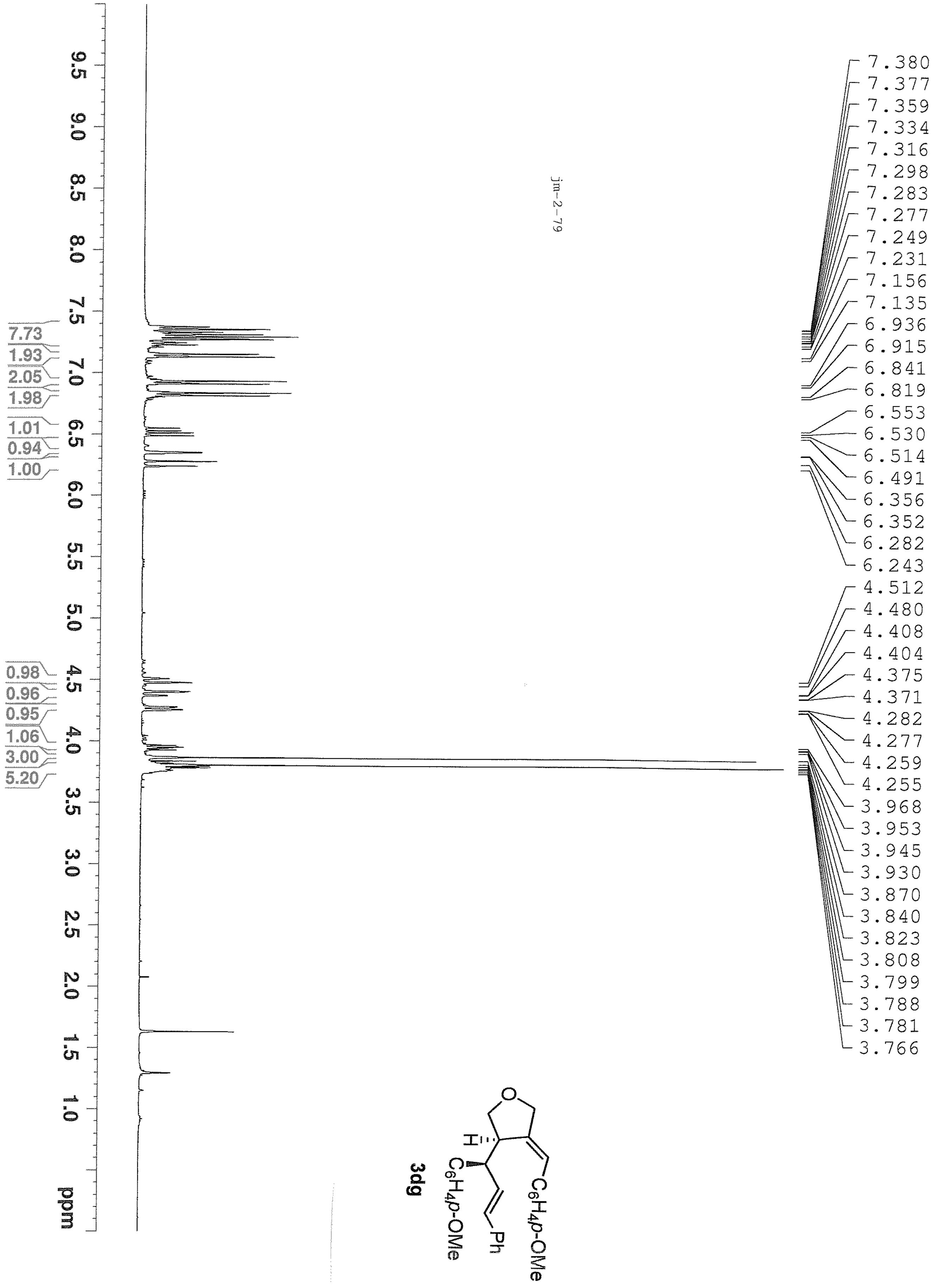
jm-2-79
7.380
7.377
7.359
7.334
7.316
7.298
7.283
7.277
7.249
7.231
7.156
7.135
6.936
6.915
6.841
6.819
6.553
6.530
6.514
6.491
6.356
6.352
6.282
6.243
4.512
4.480
4.408
4.404
4.375
4.371
4.282
4.277
4.259
4.255
3.968
3.953
3.945
3.930
3.870
3.840
3.823
3.808
3.799
3.788
3.781
3.766
7.73
1.93
2.05
1.98
1.01
0.94
1.00
0.98
0.96
0.95
1.06
3.00
5.20
9.5
9.0
8.5
8.0
7.5
7.0
6.5
6.0
5.5
5.0
4.5
4.0
3.5
3.0
2.5
2.0
1.5
1.0
ppm
O
H
C6H4p-OMe
Ph
C6H4p-OMe
3dg

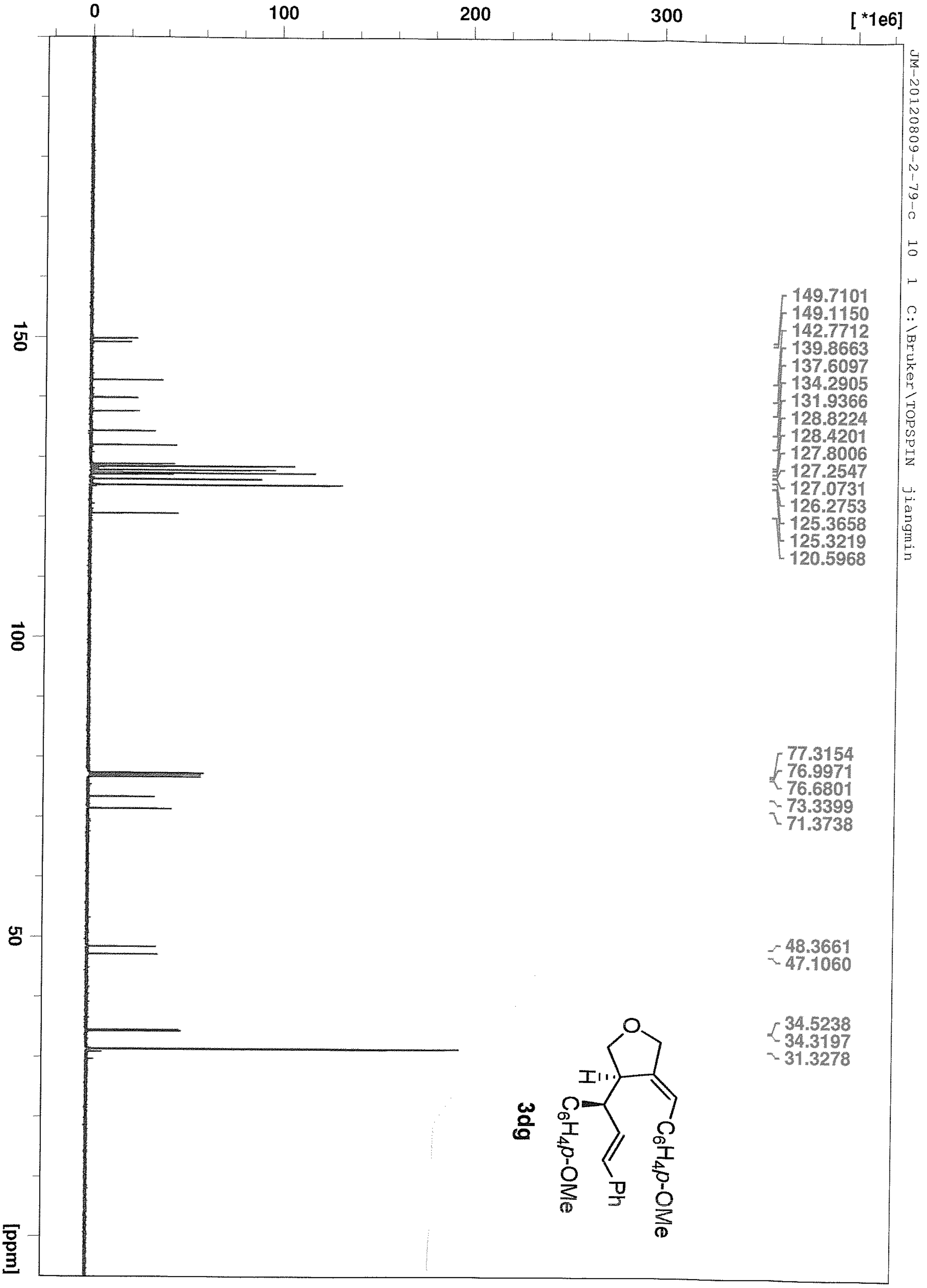
JM-20120809-2-79-c 10 1 C:\Bruker\TOPSPIN jiangmin
[*1e6]
[ppm]
149.7101
149.1150
142.7712
139.8663
137.6097
134.2905
131.9366
128.8224
128.4201
127.8006
127.2547
127.0731
126.2753
125.3658
125.3219
120.5968
77.3154
76.9971
76.6801
73.3399
71.3738
48.3661
47.1060
34.5238
34.3197
31.3278
C6H4p-OMe
Ph
C6H4p-OMe
3dg

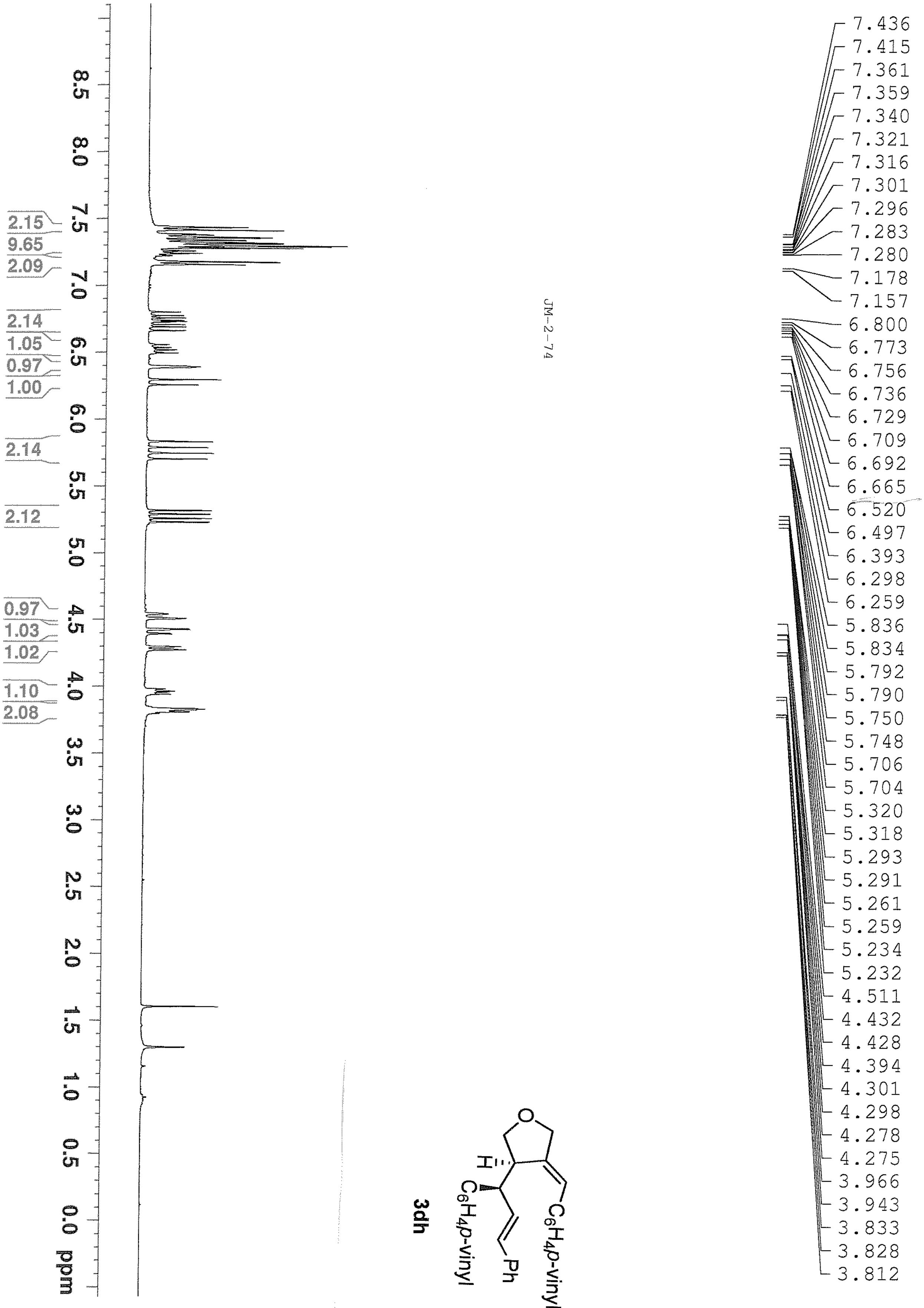
JM-2-74
7.436
7.415
7.361
7.359
7.340
7.321
7.316
7.301
7.296
7.283
7.280
7.178
7.157
6.800
6.773
6.756
6.736
6.729
6.709
6.692
6.665
6.520
6.497
6.393
6.298
6.259
5.836
5.834
5.792
5.790
5.750
5.748
5.706
5.704
5.320
5.318
5.293
5.291
5.261
5.259
5.234
5.232
4.511
4.432
4.428
4.394
4.301
4.298
4.278
4.275
3.966
3.943
3.833
3.828
3.812
2.15
9.65
2.09
2.14
1.05
0.97
1.00
2.14
2.12
0.97
1.03
1.02
1.10
2.08
8.5
8.0
7.5
7.0
6.5
6.0
5.5
5.0
4.5
4.0
3.5
3.0
2.5
2.0
1.5
1.0
0.5
0.0
ppm
C6H4p-vinyl
Ph
H
C6H4p-vinyl
3dh

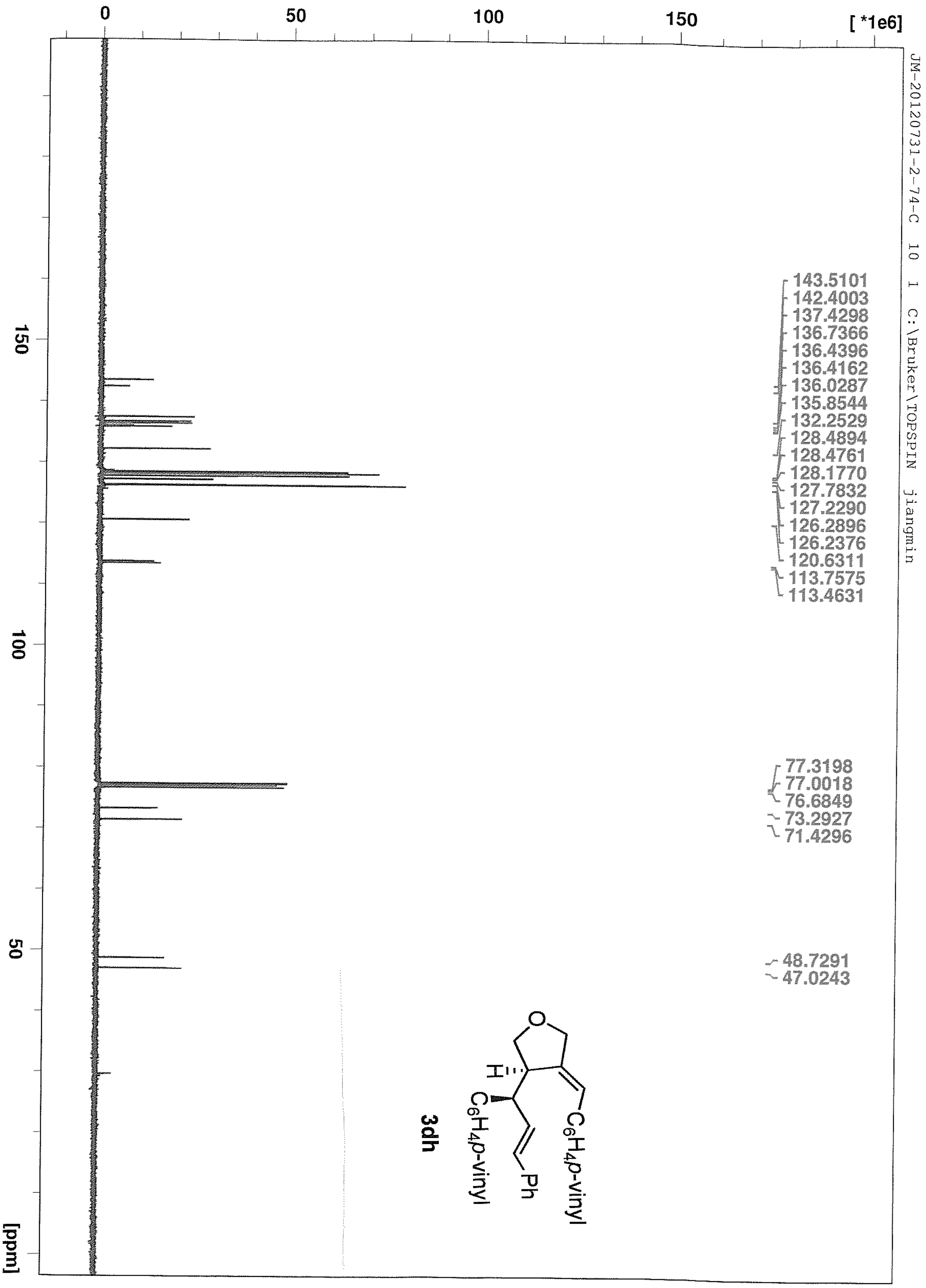
JM-20120731-2-74-C 10 1 C:\Bruker\TOPSPIN jiangmin
[*1e6]
0
50
100
150
[ppm]
143.5101
142.4003
137.4298
136.7366
136.4396
136.4162
136.0287
135.8544
132.2529
128.4894
128.4761
128.1770
127.7832
127.2290
126.2896
126.2376
120.6311
113.7575
113.4631
77.3198
77.0018
76.6849
73.2927
71.4296
48.7291
47.0243
C6H4p-vinyl
Ph
C6H4p-vinyl
3dh

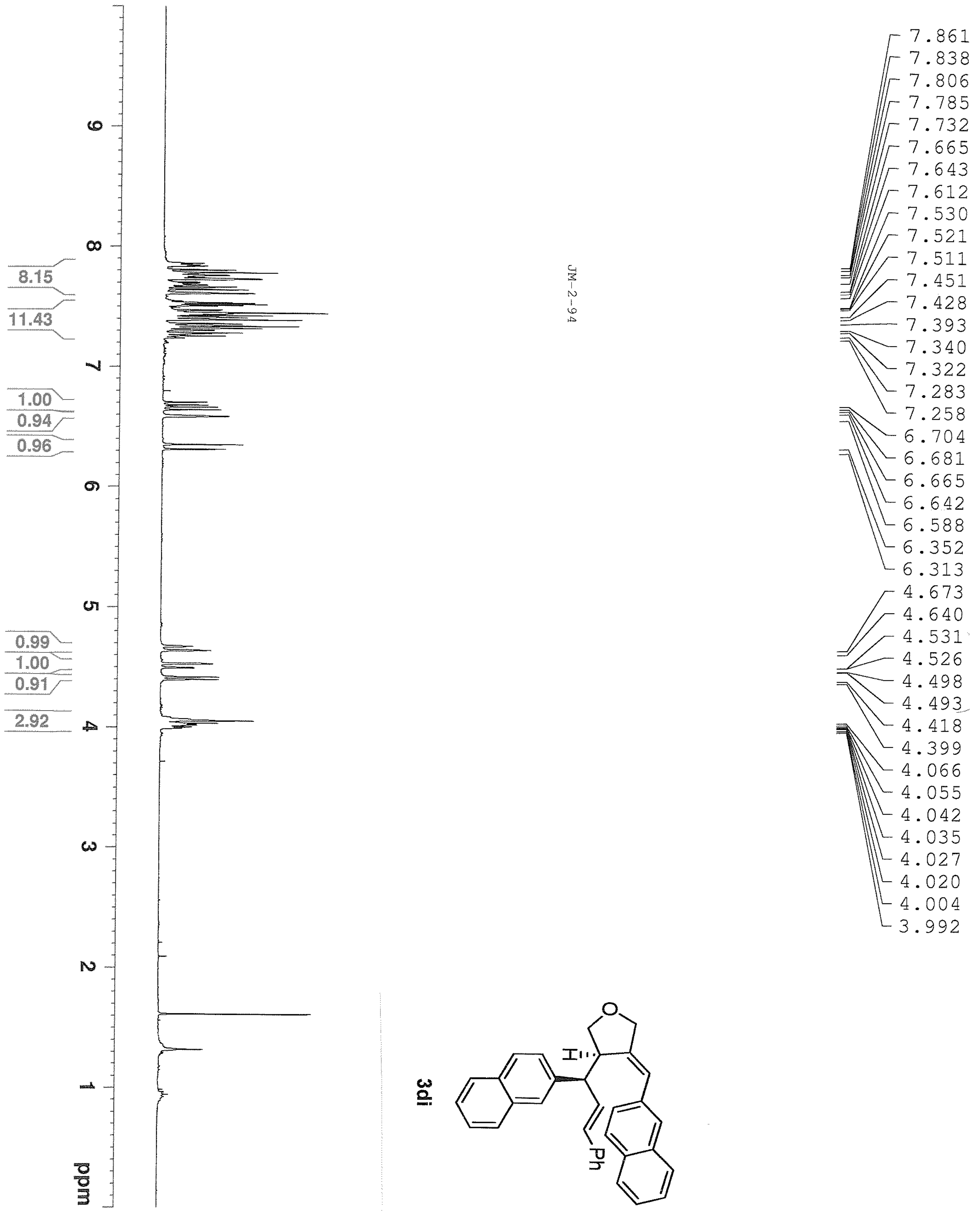
JM-2-94
7.861
7.838
7.806
7.785
7.732
7.665
7.643
7.612
7.530
7.521
7.511
7.451
7.428
7.393
7.340
7.322
7.283
7.258
6.704
6.681
6.665
6.642
6.588
6.352
6.313
4.673
4.640
4.531
4.526
4.498
4.493
4.418
4.399
4.066
4.055
4.042
4.035
4.027
4.020
4.004
3.992
8.15
11.43
1.00
0.94
0.96
0.99
1.00
0.91
2.92
9
8
7
6
5
4
3
2
1
ppm
3di
Ph
O
H

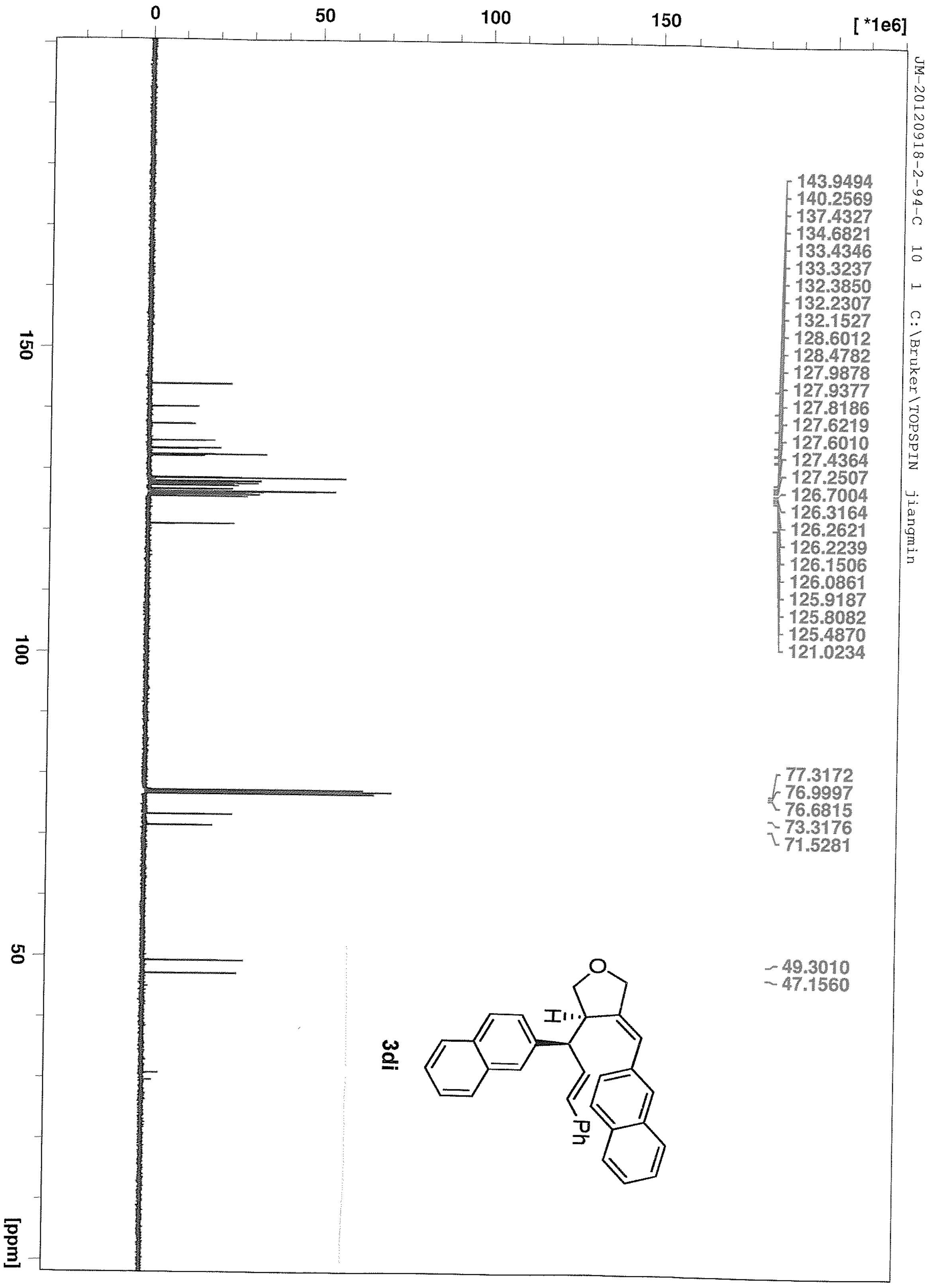
JM-20120918-2-94-C 10 1 C:\Bruker\TOPSPIN jiangmin
143.9494
140.2569
137.4327
134.6821
133.4346
133.3237
132.3850
132.2307
132.1527
128.6012
128.4782
127.9878
127.9377
127.8186
127.6219
127.6010
127.4364
127.2507
126.7004
126.3164
126.2621
126.2239
126.1506
126.0861
125.9187
125.8082
125.4870
121.0234
77.3172
76.9997
76.6815
73.3176
71.5281
49.3010
47.1560
3di
Ph
[ppm]
[*1e6]

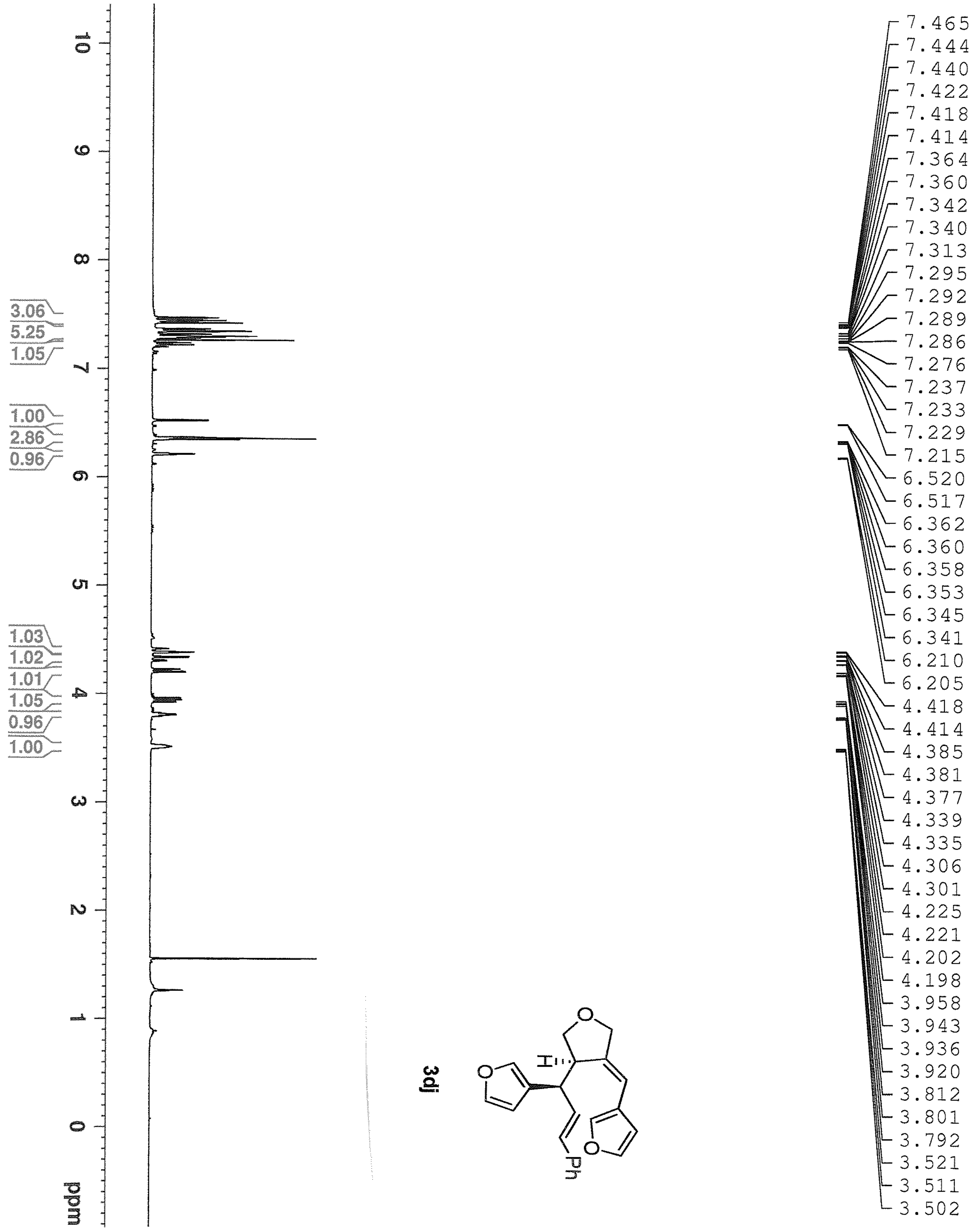
3dj
Ph
ppm
3.06
5.25
1.05
1.00
2.86
0.96
1.03
1.02
1.01
1.05
0.96
1.00
7.465
7.444
7.440
7.422
7.418
7.414
7.364
7.360
7.342
7.340
7.313
7.295
7.292
7.289
7.286
7.276
7.237
7.233
7.229
7.215
6.520
6.517
6.362
6.360
6.358
6.353
6.345
6.341
6.210
6.205
4.418
4.414
4.385
4.381
4.377
4.339
4.335
4.306
4.301
4.225
4.221
4.202
4.198
3.958
3.943
3.936
3.920
3.812
3.801
3.792
3.521
3.511
3.502

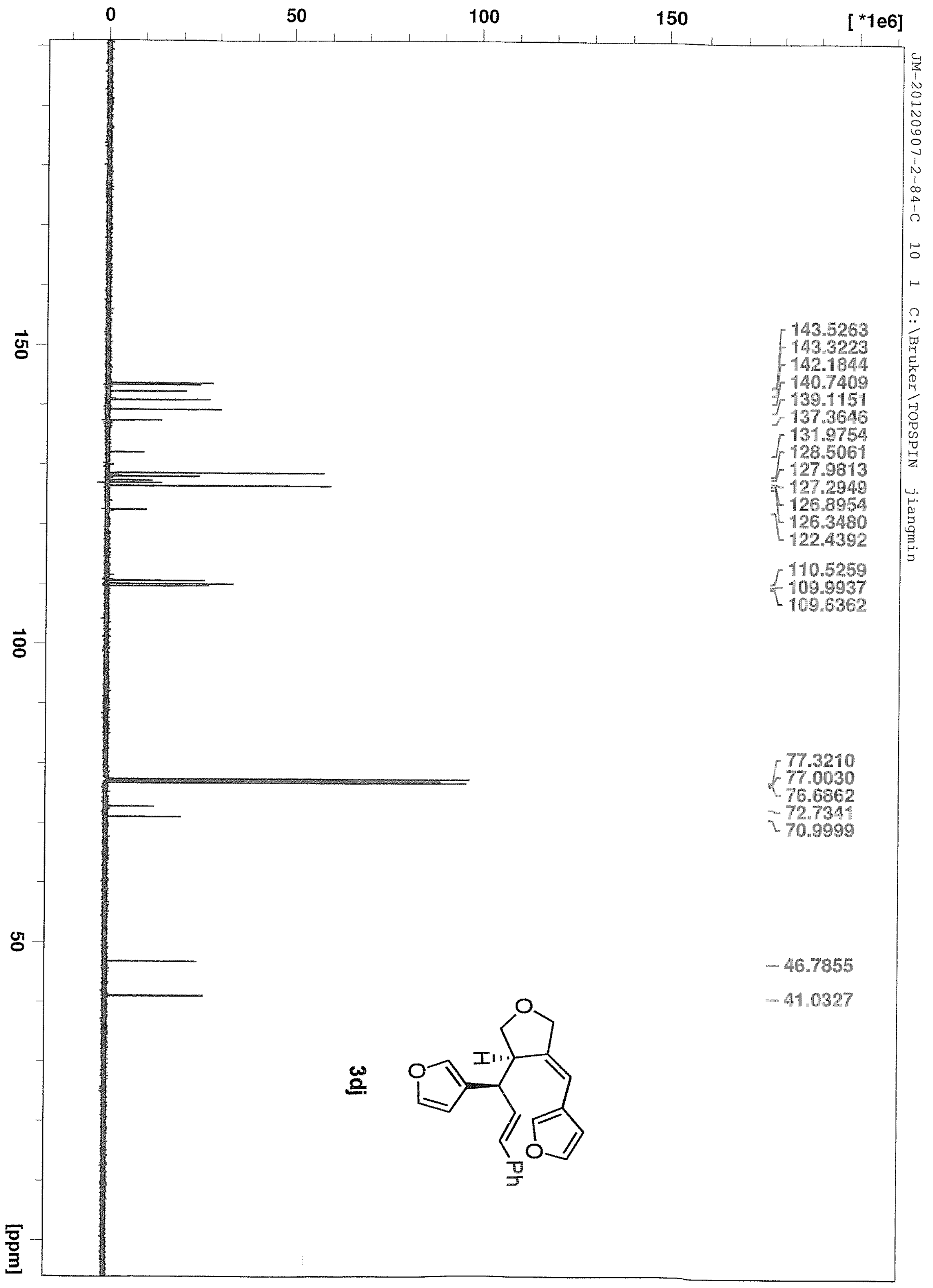
JM-20120907-2-84-C 10 1 C:\Bruker\TOPSPIN jiangmin
143.5263
143.3223
142.1844
140.7409
139.1151
137.3646
131.9754
128.5061
127.9813
127.2949
126.8954
126.3480
122.4392
110.5259
109.9937
109.6362
77.3210
77.0030
76.6862
72.7341
70.9999
46.7855
41.0327
3dj
Ph
[ppm]
[*1e6]

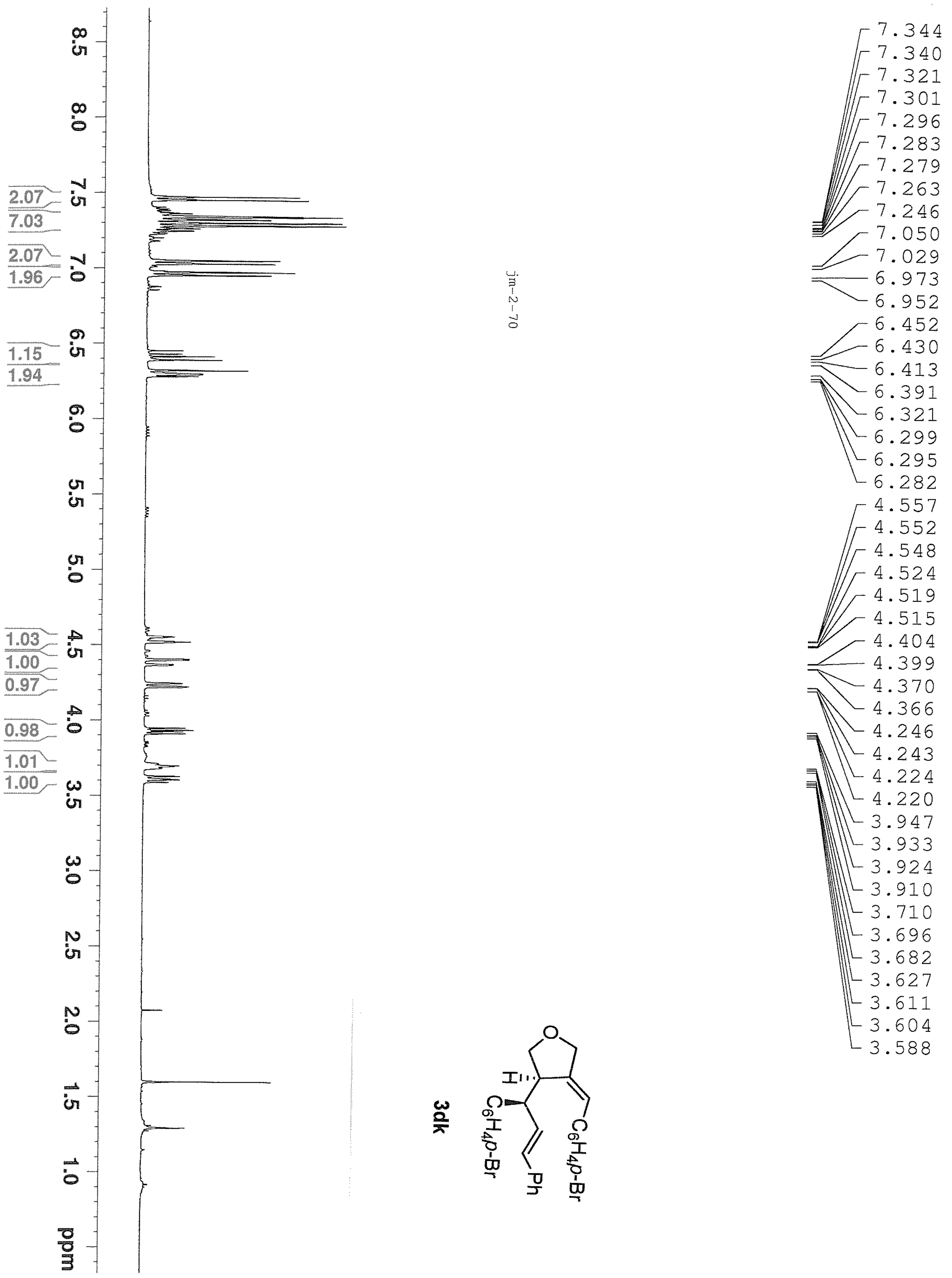
7.344
7.340
7.321
7.301
7.296
7.283
7.279
7.263
7.246
7.050
7.029
6.973
6.952
6.452
6.430
6.413
6.391
6.321
6.299
6.295
6.282
4.557
4.552
4.548
4.524
4.519
4.515
4.404
4.399
4.370
4.366
4.246
4.243
4.224
4.220
3.947
3.933
3.924
3.910
3.710
3.696
3.682
3.627
3.611
3.604
3.588
jm-2-70
2.07
7.03
2.07
1.96
1.15
1.94
1.03
1.00
0.97
0.98
1.01
1.00
8.5
8.0
7.5
7.0
6.5
6.0
5.5
5.0
4.5
4.0
3.5
3.0
2.5
2.0
1.5
1.0
ppm
O
H
C_6H_4p-Br
Ph
C_6H_4p-Br
3dk

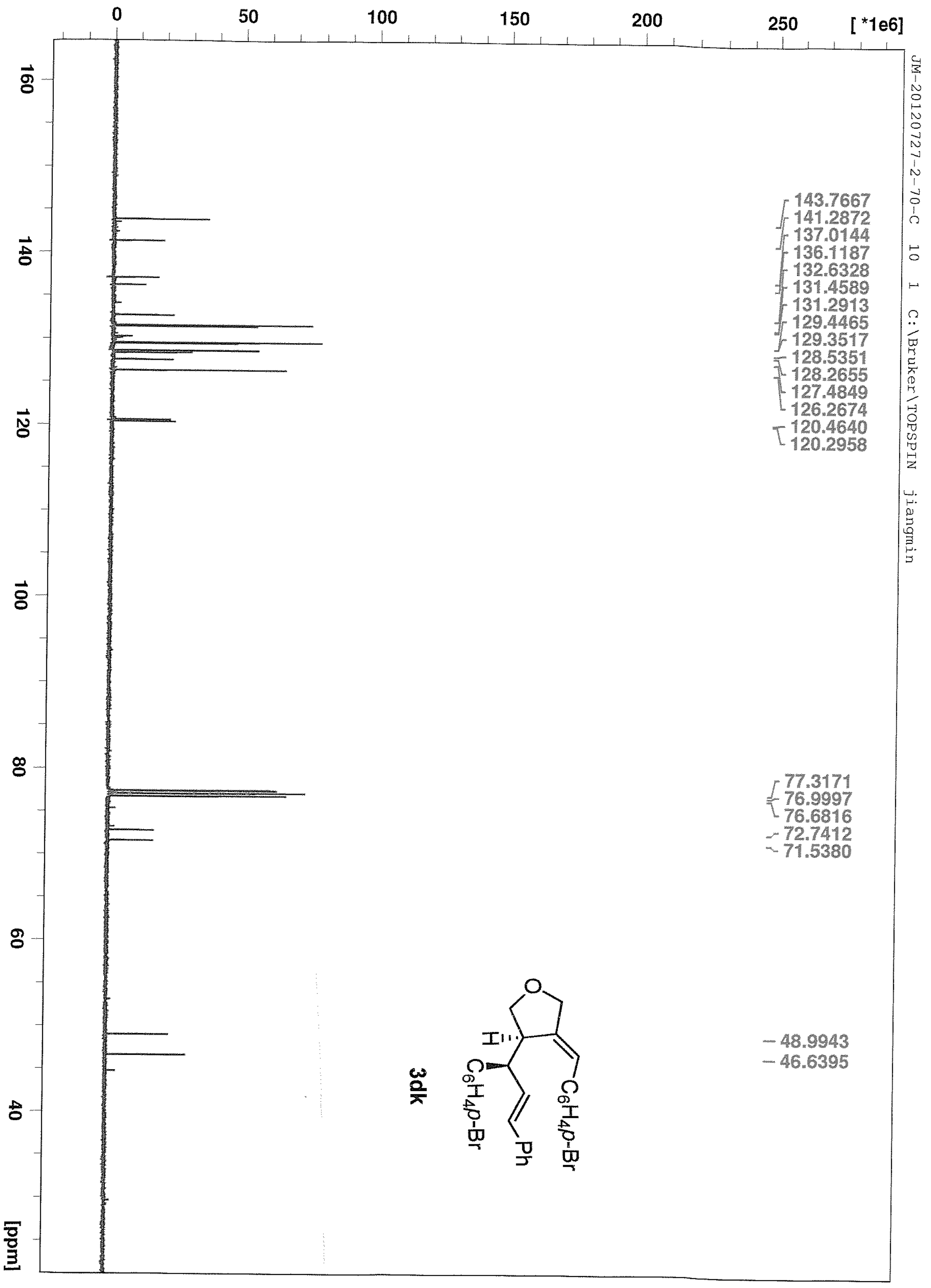
JM-20120727-2-70-C 10 1 C:\Bruker\TOPSPIN jiangmin
143.7667
141.2872
137.0144
136.1187
132.6328
131.4589
131.2913
129.4465
129.3517
128.5351
128.2655
127.4849
126.2674
120.4640
120.2958
77.3171
76.9997
76.6816
72.7412
71.5380
48.9943
46.6395
3dk
[ppm]
[*1e6]

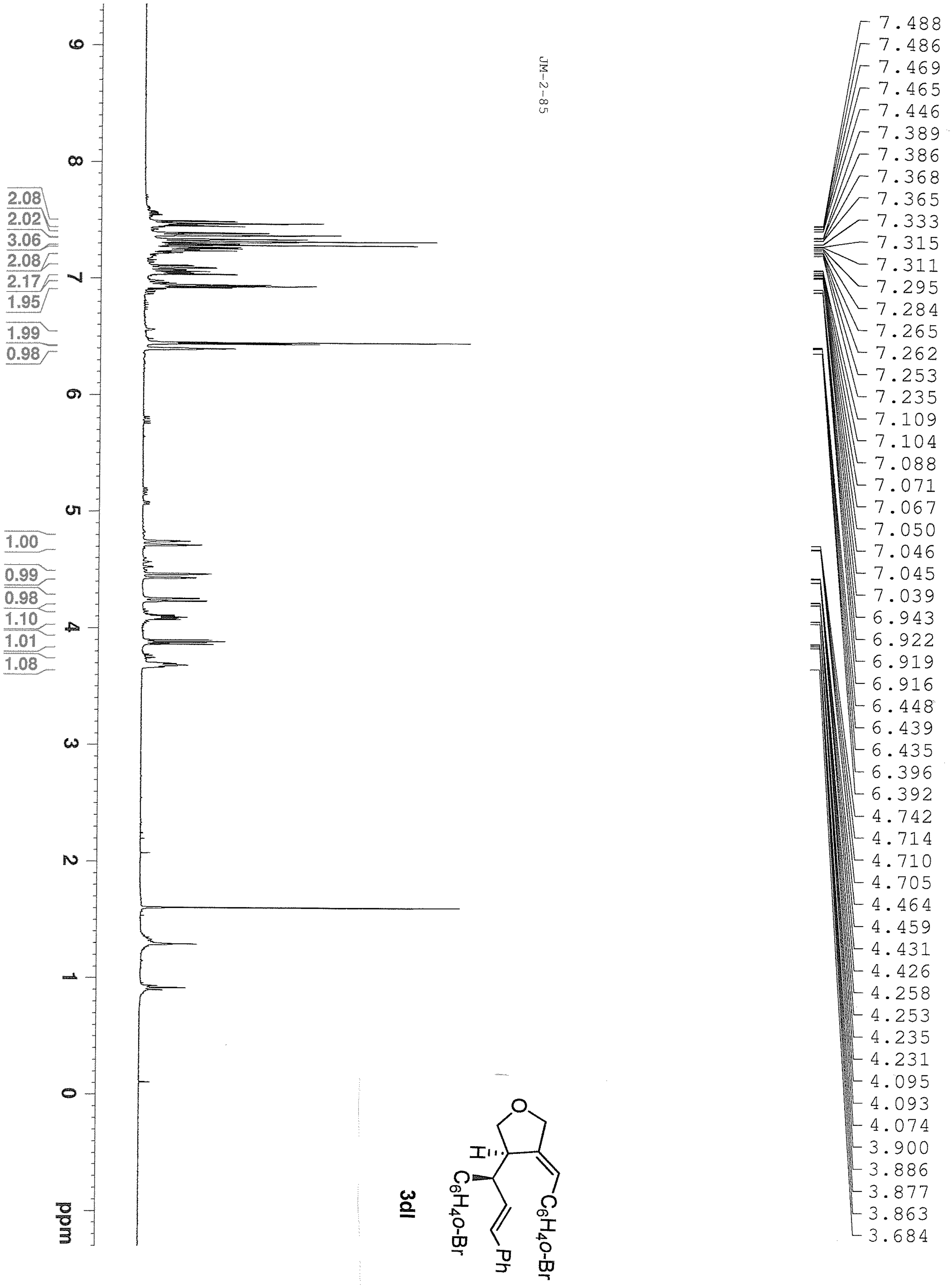
JM-2-85
7.488
7.486
7.469
7.465
7.446
7.389
7.386
7.368
7.365
7.333
7.315
7.311
7.295
7.284
7.265
7.262
7.253
7.235
7.109
7.104
7.088
7.071
7.067
7.050
7.046
7.045
7.039
6.943
6.922
6.919
6.916
6.448
6.439
6.435
6.396
6.392
4.742
4.714
4.710
4.705
4.464
4.459
4.431
4.426
4.258
4.253
4.235
4.231
4.095
4.093
4.074
3.900
3.886
3.877
3.863
3.684
2.08
2.02
3.06
2.08
2.17
1.95
1.99
0.98
1.00
0.99
0.98
1.10
1.01
1.08
9
8
7
6
5
4
3
2
1
0
ppm
C6H4o-Br
Ph
H
C6H4o-Br
3dl

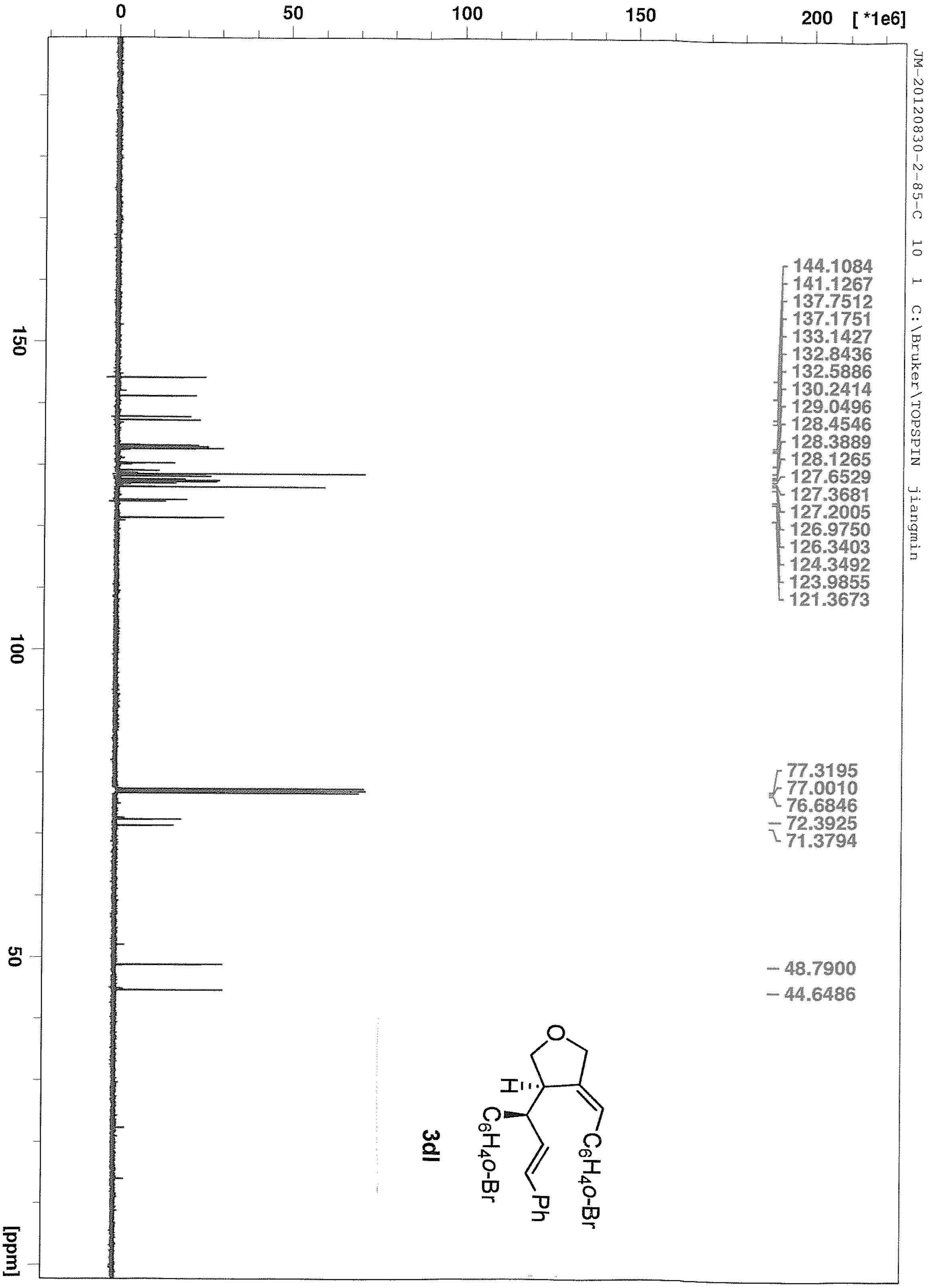
JM-20120830-2-85-C 10 1 C:\Bruker\TOPSPIN jiangmin
144.1084
141.1267
137.7512
137.1751
133.1427
132.8436
132.5886
130.2414
129.0496
128.4546
128.3889
128.1265
127.6529
127.3681
127.2005
126.9750
126.3403
124.3492
123.9855
121.3673
77.3195
77.0010
76.6846
72.3925
71.3794
48.7900
44.6486
3dl
[ppm]
[*1e6]

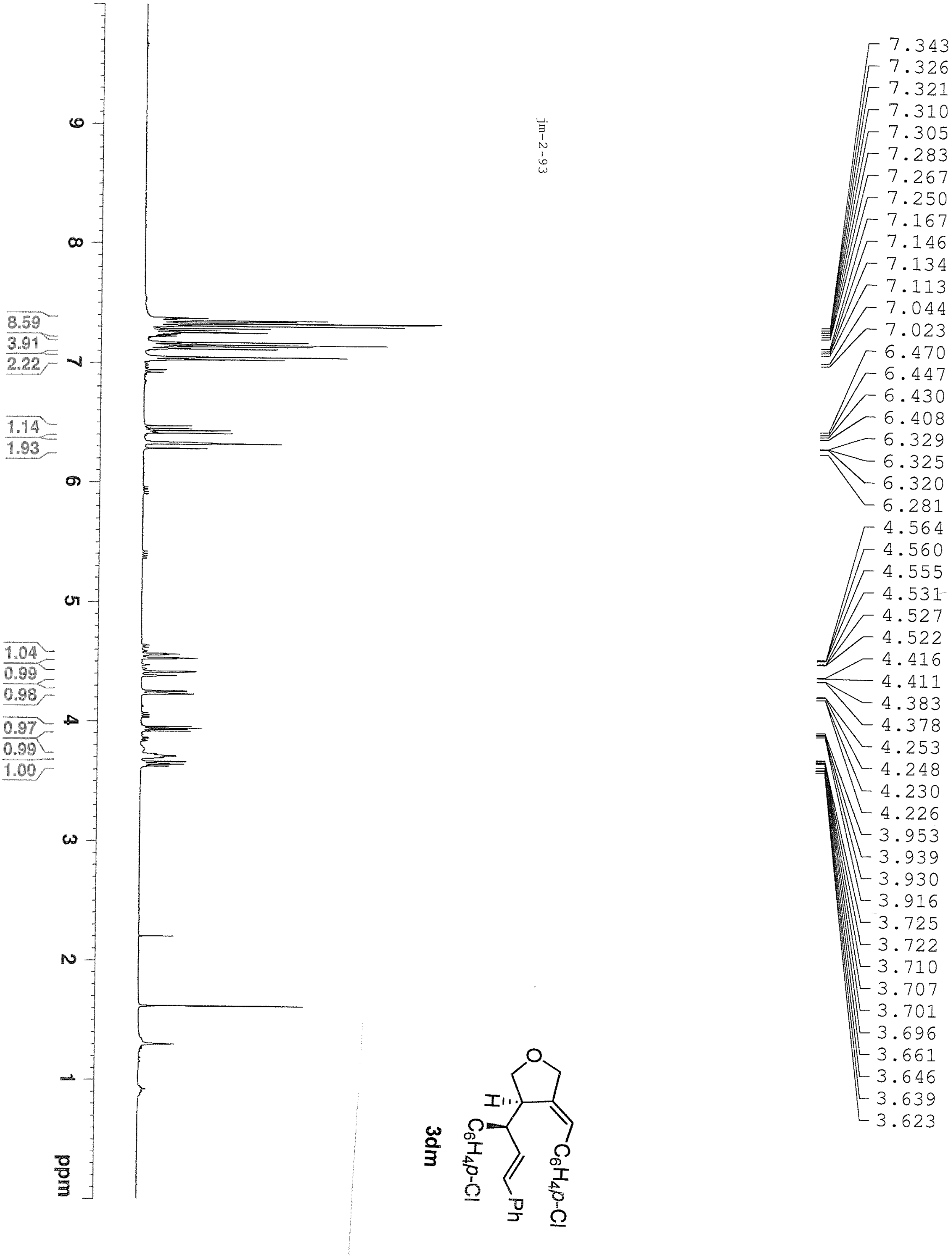
jm-2-93
7.343
7.326
7.321
7.310
7.305
7.283
7.267
7.250
7.167
7.146
7.134
7.113
7.044
7.023
6.470
6.447
6.430
6.408
6.329
6.325
6.320
6.281
4.564
4.560
4.555
4.531
4.527
4.522
4.416
4.411
4.383
4.378
4.253
4.248
4.230
4.226
3.953
3.939
3.930
3.916
3.725
3.722
3.710
3.707
3.701
3.696
3.661
3.646
3.639
3.623
8.59
3.91
2.22
1.14
1.93
1.04
0.99
0.98
0.97
0.99
1.00
9
8
7
6
5
4
3
2
1
ppm
O
H
C_6H_4p-Cl
Ph
C_6H_4p-Cl
3dm

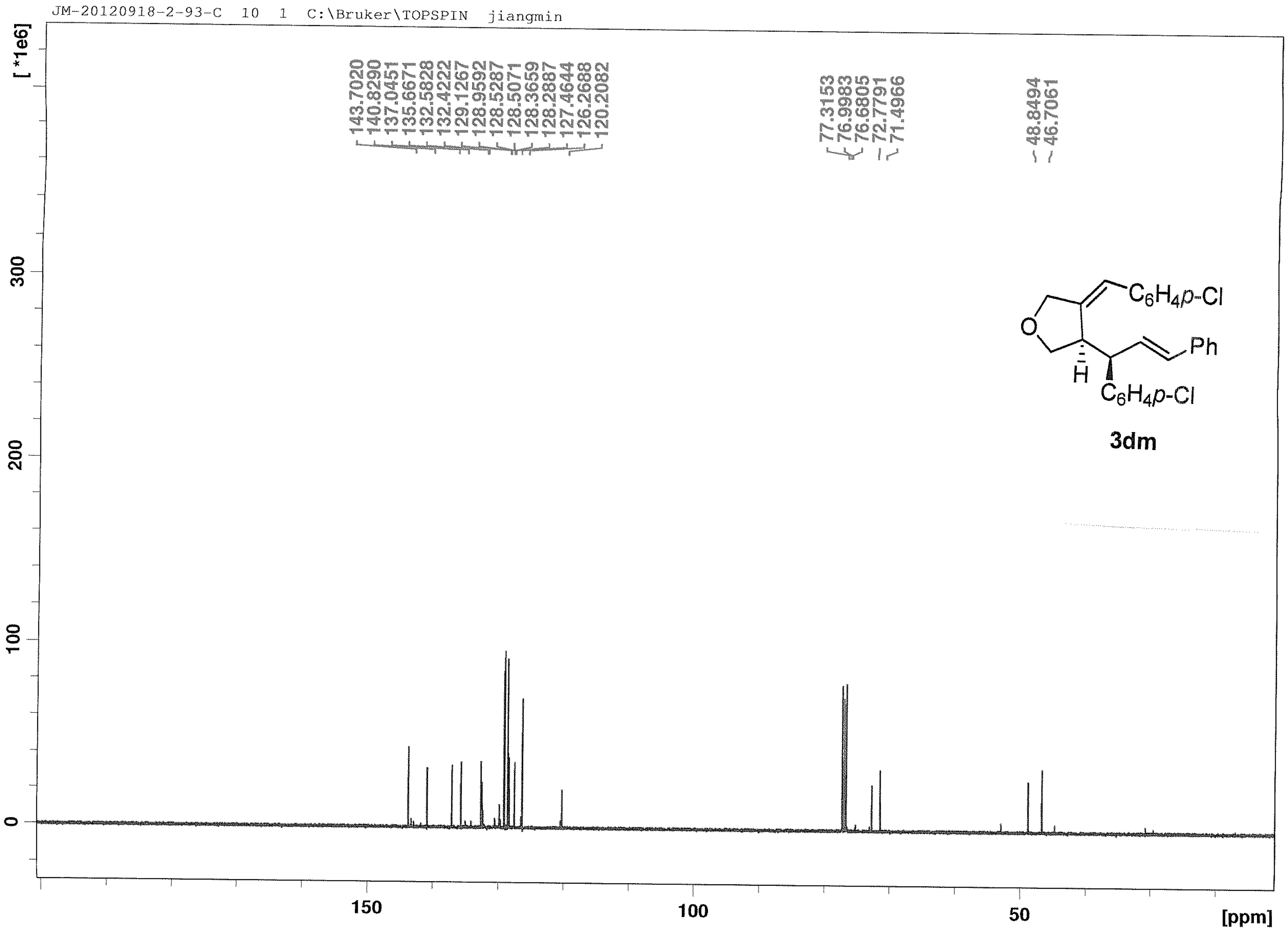
JM-20120918-2-93-C 10 1 C:\Bruker\TOPSPIN jiangmin
[*1e6]
300
200
100
0
143.7020
140.8290
137.0451
135.6671
132.5828
132.4222
129.1267
128.9592
128.5287
128.5071
128.3659
128.2887
127.4644
126.2688
120.2082
77.3153
76.9983
76.6805
72.7791
71.4966
48.8494
46.7061
O
C_6H_4p-Cl
Ph
H
C_6H_4p-Cl
3dm
150
100
50
[ppm]

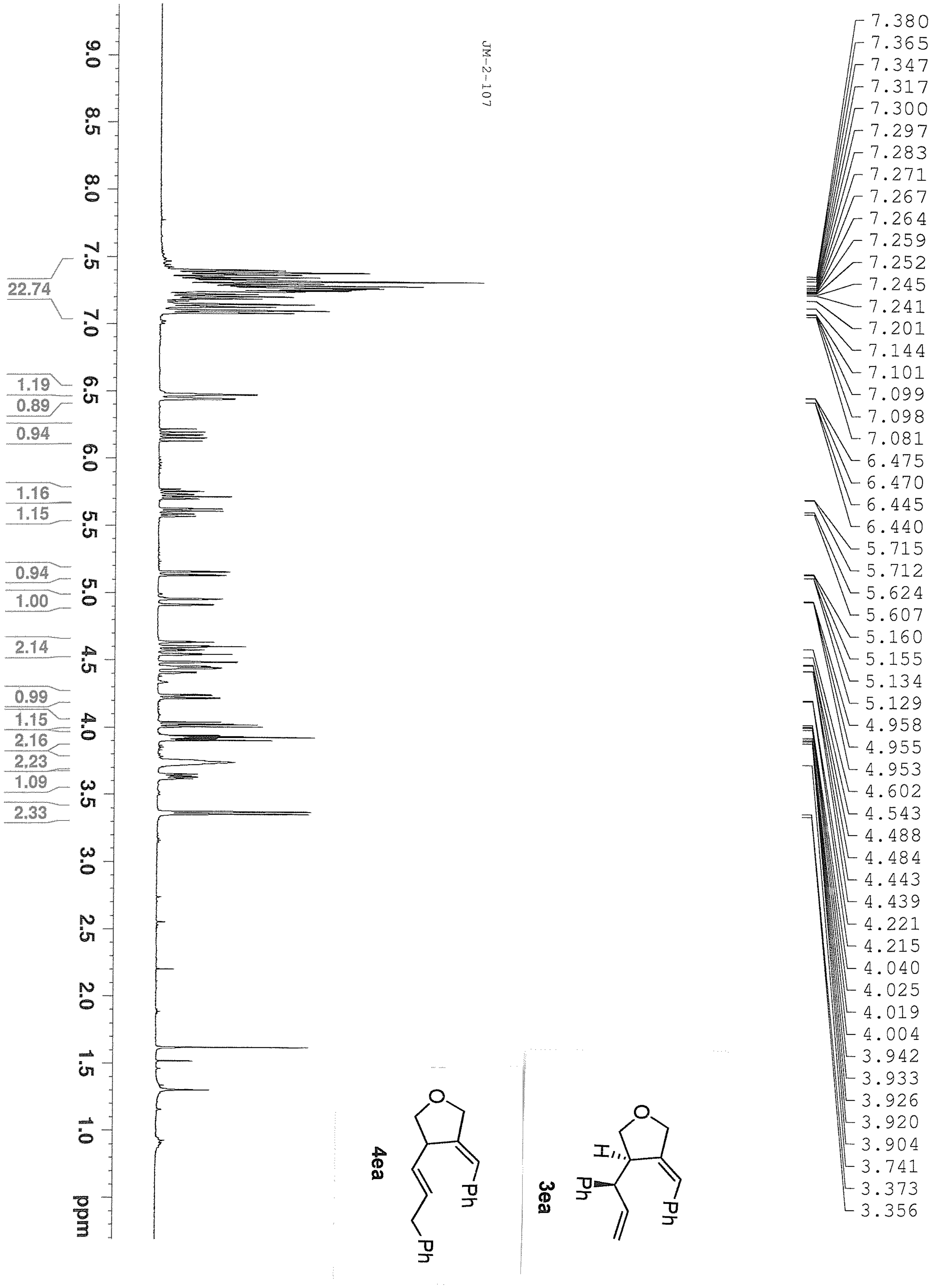
JM-2-107
7.380
7.365
7.347
7.317
7.300
7.297
7.283
7.271
7.267
7.264
7.259
7.252
7.245
7.241
7.201
7.144
7.101
7.099
7.098
7.081
6.475
6.470
6.445
6.440
5.715
5.712
5.624
5.607
5.160
5.155
5.134
5.129
4.958
4.955
4.953
4.602
4.543
4.488
4.484
4.443
4.439
4.221
4.215
4.040
4.025
4.019
4.004
3.942
3.933
3.926
3.920
3.904
3.741
3.373
3.356
22.74
1.19
0.89
0.94
1.16
1.15
0.94
1.00
2.14
0.99
1.15
2.16
2.23
1.09
2.33
9.0
8.5
8.0
7.5
7.0
6.5
6.0
5.5
5.0
4.5
4.0
3.5
3.0
2.5
2.0
1.5
1.0
ppm
4ea
3ea
Ph
H

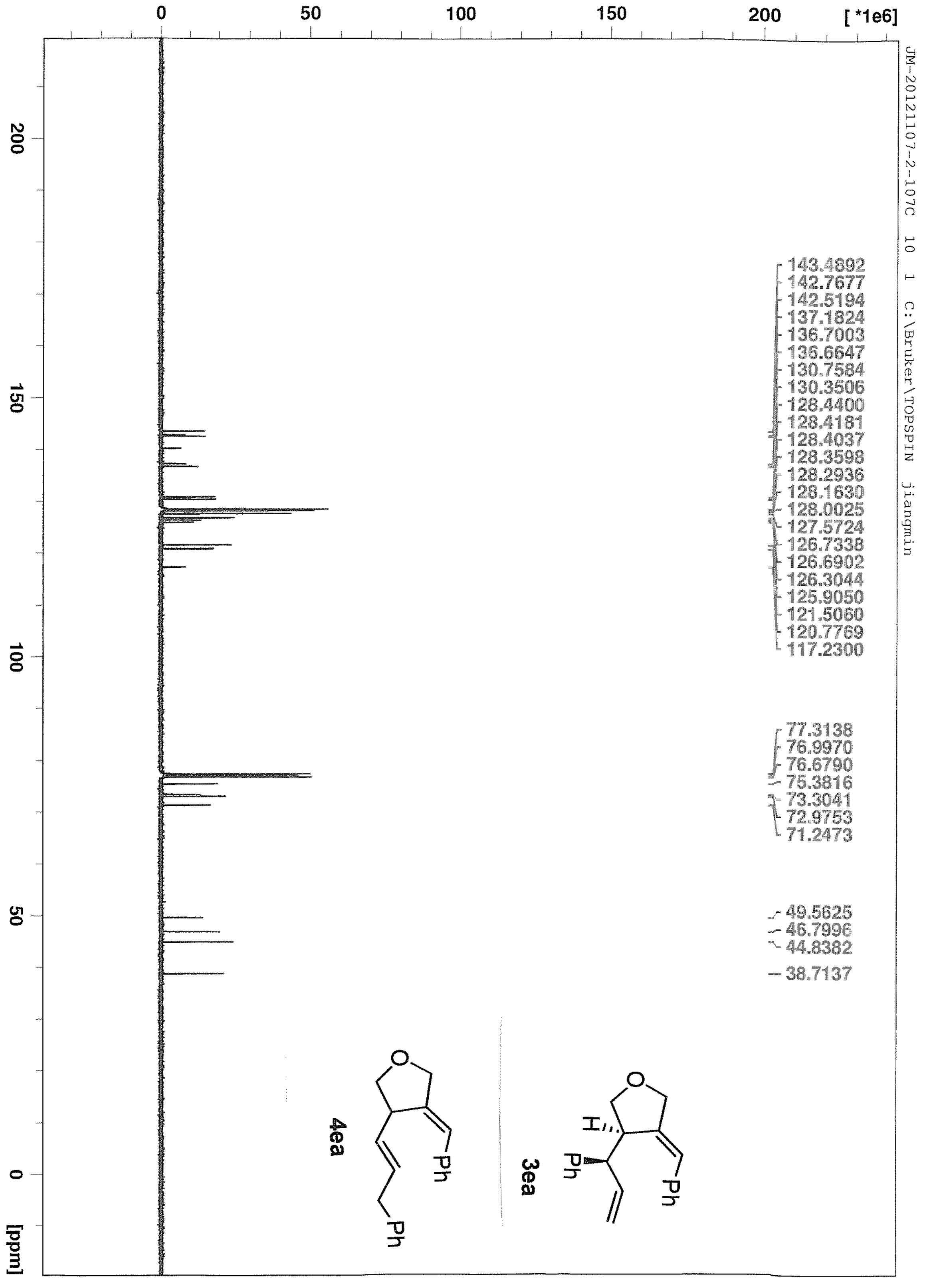
JM-20121107-2-107C 10 1 C:\Bruker\TOPSPIN jiangmin
143.4892
142.7677
142.5194
137.1824
136.7003
136.6647
130.7584
130.3506
128.4400
128.4181
128.4037
128.3598
128.2936
128.1630
128.0025
127.5724
126.7338
126.6902
126.3044
125.9050
121.5060
120.7769
117.2300
77.3138
76.9970
76.6790
75.3816
73.3041
72.9753
71.2473
49.5625
46.7996
44.8382
38.7137
Ph
O
H
Ph
3ea
Ph
O
Ph
4ea
[*1e6]
[ppm]

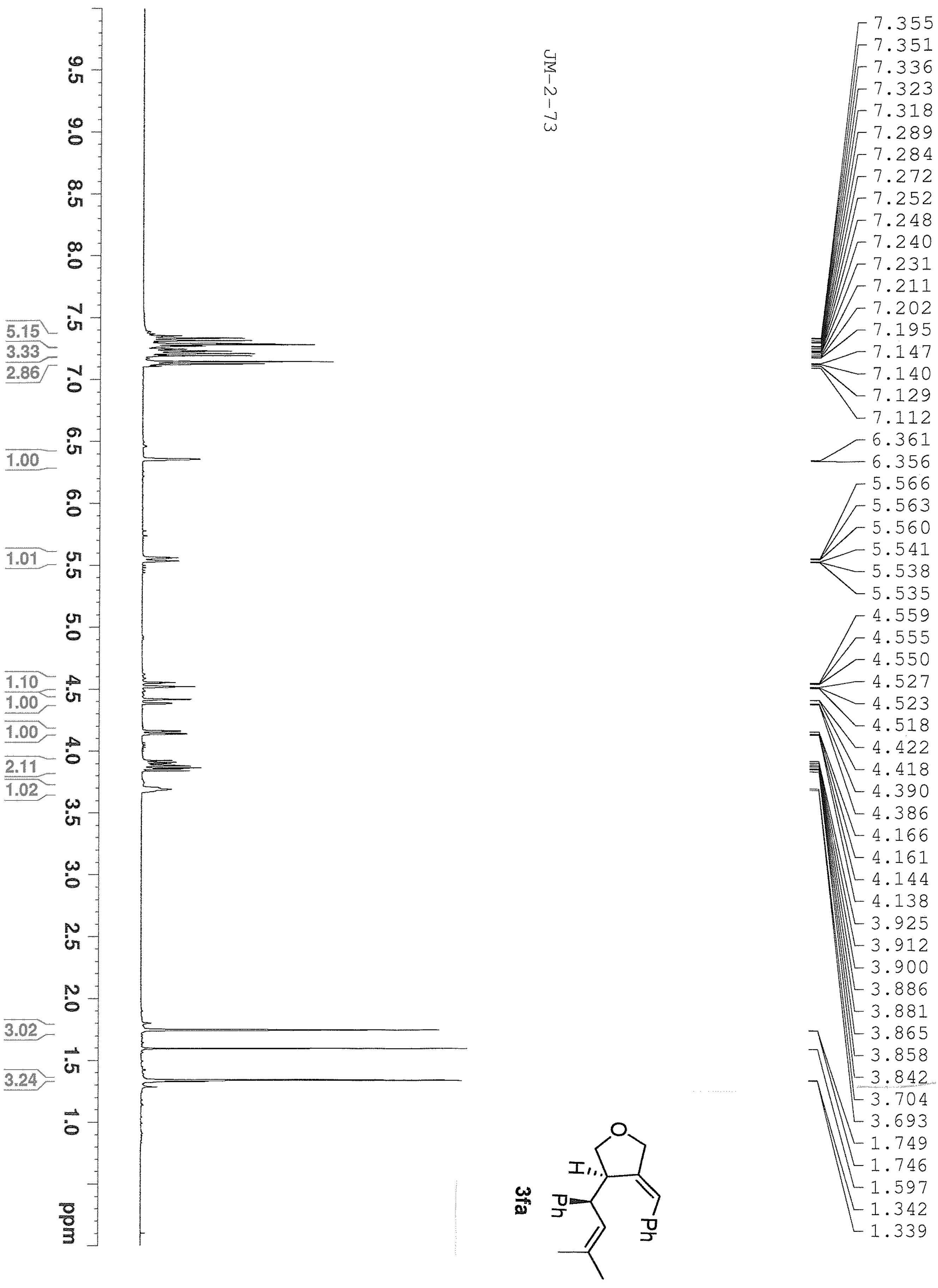
JM-2-73
5.15
3.33
2.86
1.00
1.01
1.10
1.00
1.00
2.11
1.02
3.02
3.24
9.5 9.0 8.5 8.0 7.5 7.0 6.5 6.0 5.5 5.0 4.5 4.0 3.5 3.0 2.5 2.0 1.5 1.0 ppm
7.355
7.351
7.336
7.323
7.318
7.289
7.284
7.272
7.252
7.248
7.240
7.231
7.211
7.202
7.195
7.147
7.140
7.129
7.112
6.361
6.356
5.566
5.563
5.560
5.541
5.538
5.535
4.559
4.555
4.550
4.527
4.523
4.518
4.422
4.418
4.390
4.386
4.166
4.161
4.144
4.138
3.925
3.912
3.900
3.886
3.881
3.865
3.858
3.842
3.704
3.693
1.749
1.746
1.597
1.342
1.339
O
H
Ph
Ph
3fa

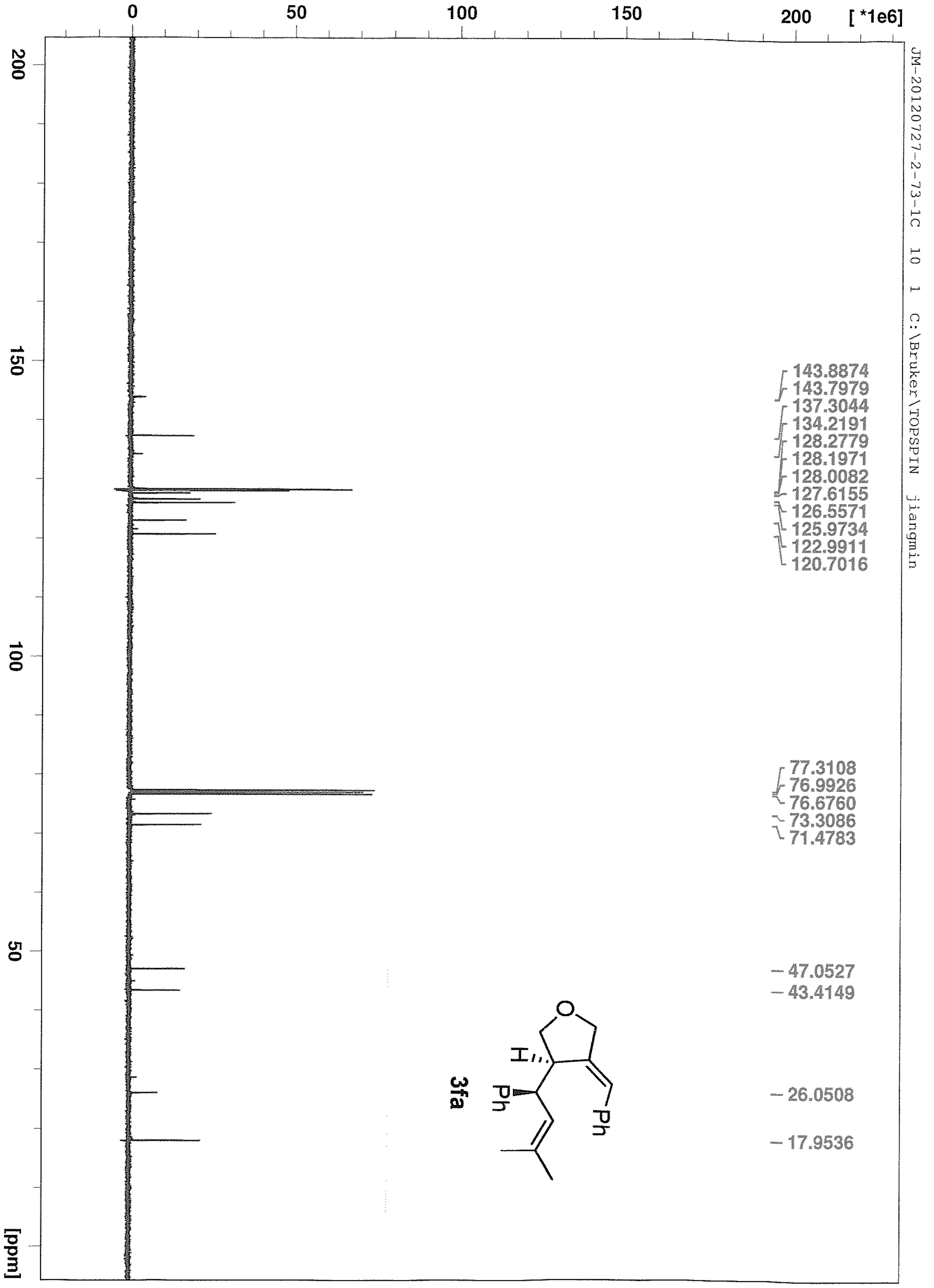
JM-20120727-2-73-1C 10 1 C:\Bruker\TOPSPIN jiangmin
[*1e6]
143.8874
143.7979
137.3044
134.2191
128.2779
128.1971
128.0082
127.6155
126.5571
125.9734
122.9911
120.7016
77.3108
76.9926
76.6760
73.3086
71.4783
47.0527
43.4149
26.0508
17.9536
[ppm]
3fa

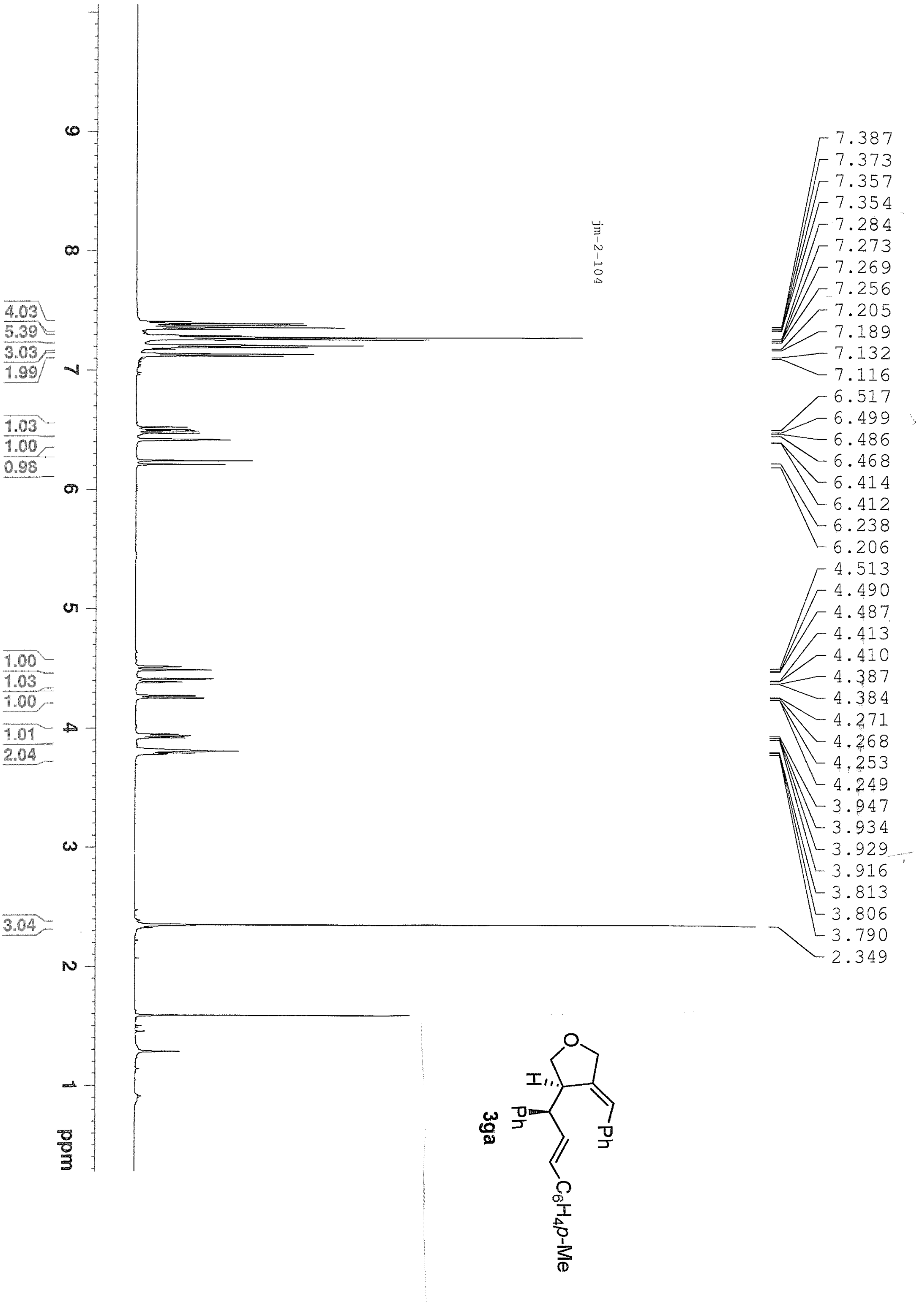
jm-2-104
7.387
7.373
7.357
7.354
7.284
7.273
7.269
7.256
7.205
7.189
7.132
7.116
6.517
6.499
6.486
6.468
6.414
6.412
6.238
6.206
4.513
4.490
4.487
4.413
4.410
4.387
4.384
4.271
4.268
4.253
4.249
3.947
3.934
3.929
3.916
3.813
3.806
3.790
2.349
4.03
5.39
3.03
1.99
1.03
1.00
0.98
1.00
1.03
1.00
1.01
2.04
3.04
9
8
7
6
5
4
3
2
1
ppm
O
H
Ph
Ph
C6H4p-Me
3ga

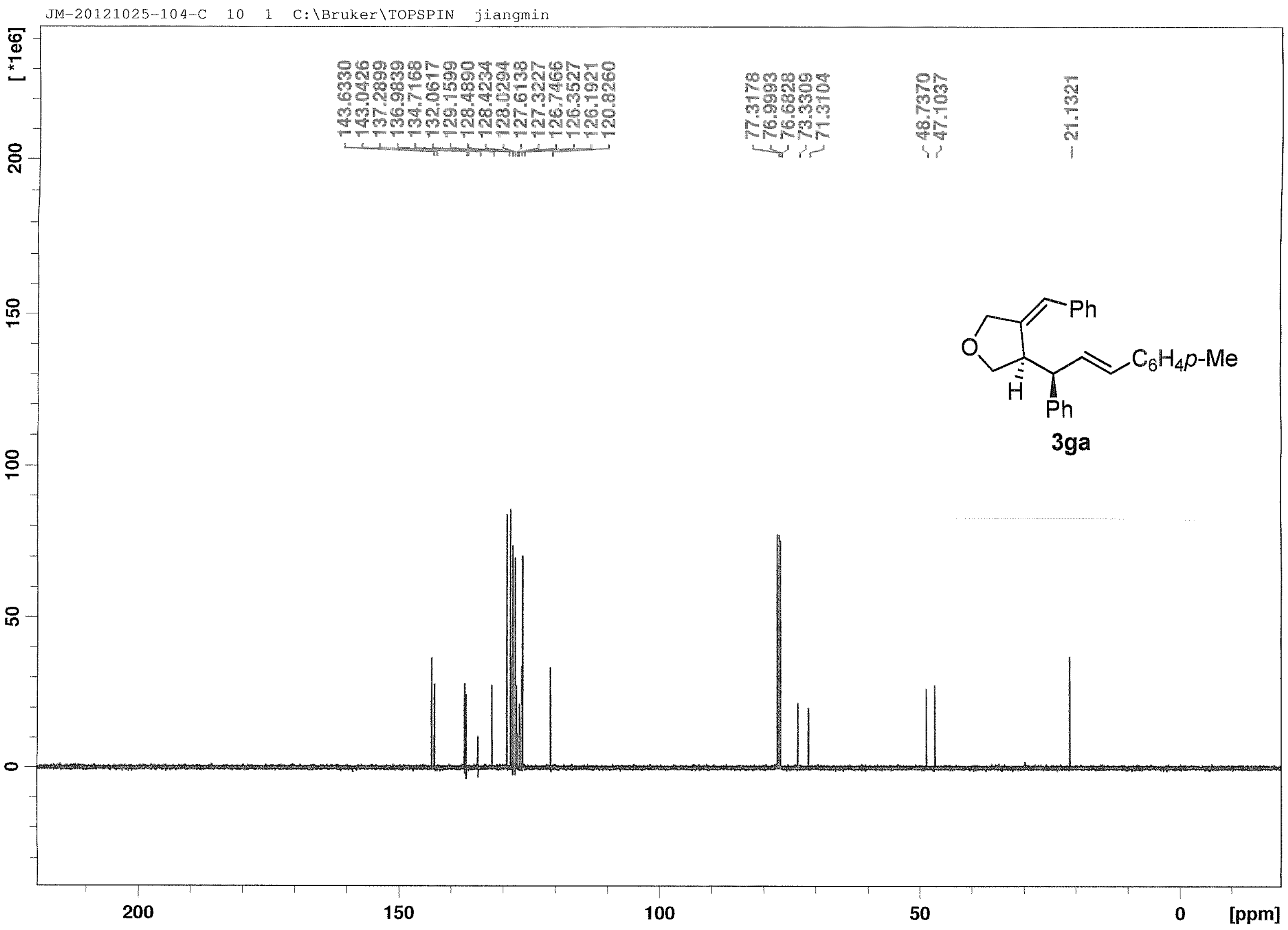
JM-20121025-104-C 10 1 C:\Bruker\TOPSPIN jiangmin
[*1e6]
143.6330
143.0426
137.2899
136.9839
134.7168
132.0617
129.1599
128.4890
128.4234
128.0294
127.6138
127.3227
126.7466
126.3527
126.1921
120.8260
77.3178
76.9993
76.6828
73.3309
71.3104
48.7370
47.1037
21.1321
Ph
O
H
Ph
C6H4p-Me
3ga
200
150
100
50
0
[ppm]

JM-2-80

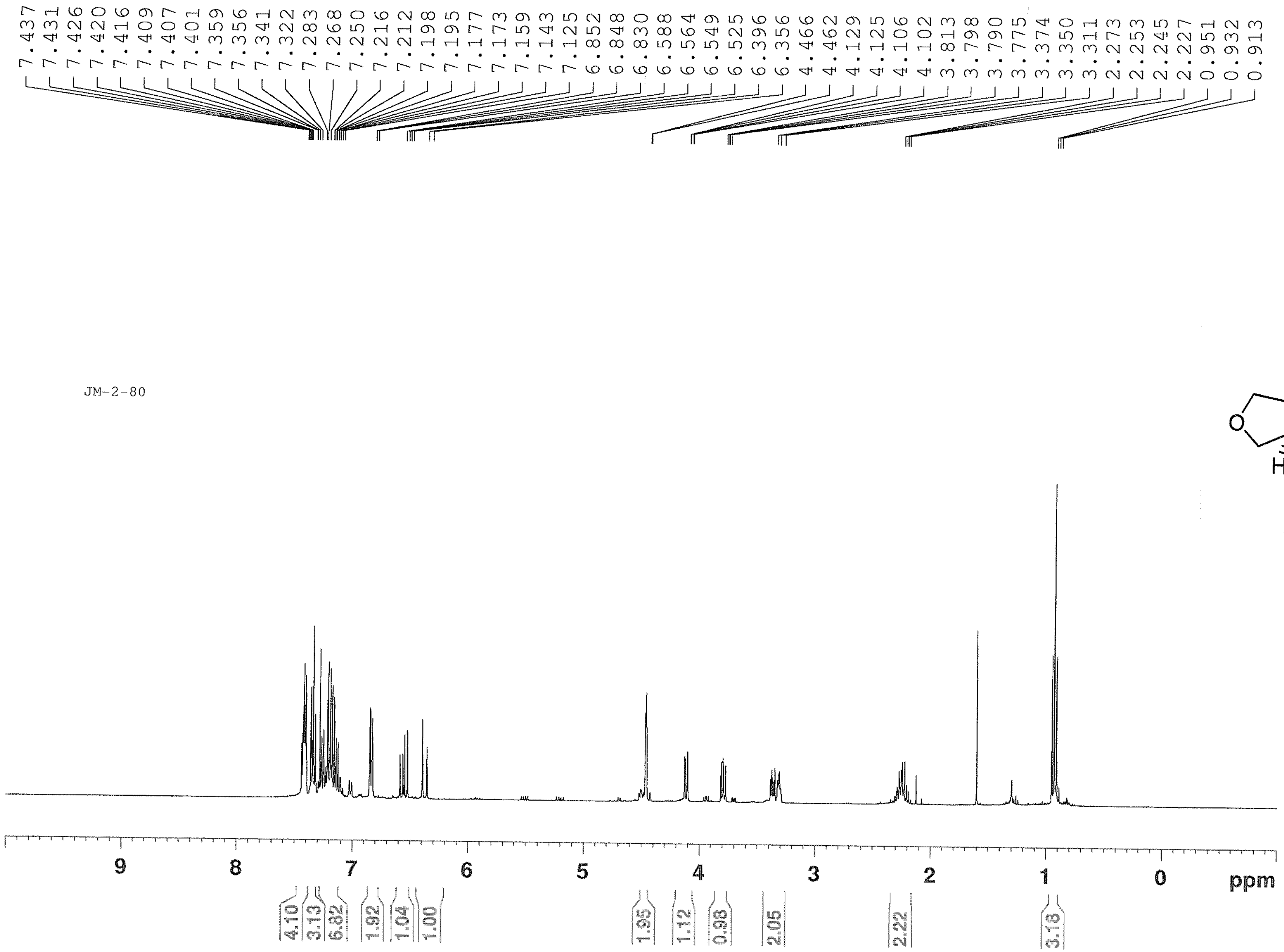
7.437
7.431
7.426
7.420
7.416
7.409
7.407
7.401
7.359
7.356
7.341
7.322
7.283
7.268
7.250
7.216
7.212
7.198
7.195
7.177
7.173
7.159
7.143
7.125
6.852
6.848
6.830
6.588
6.564
6.549
6.525
6.396
6.356
4.466
4.462
4.129
4.125
4.106
4.102
3.813
3.798
3.790
3.775
3.374
3.350
3.311
2.273
2.253
2.245
2.227
0.951
0.932
0.913
9
8
7
6
5
4
3
2
1
0
ppm
4.10
3.13
6.82
1.92
1.04
1.00
1.95
1.12
0.98
2.05
2.22
3.18

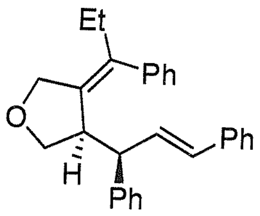
Et
Ph
O
Ph
H
Ph

3ha

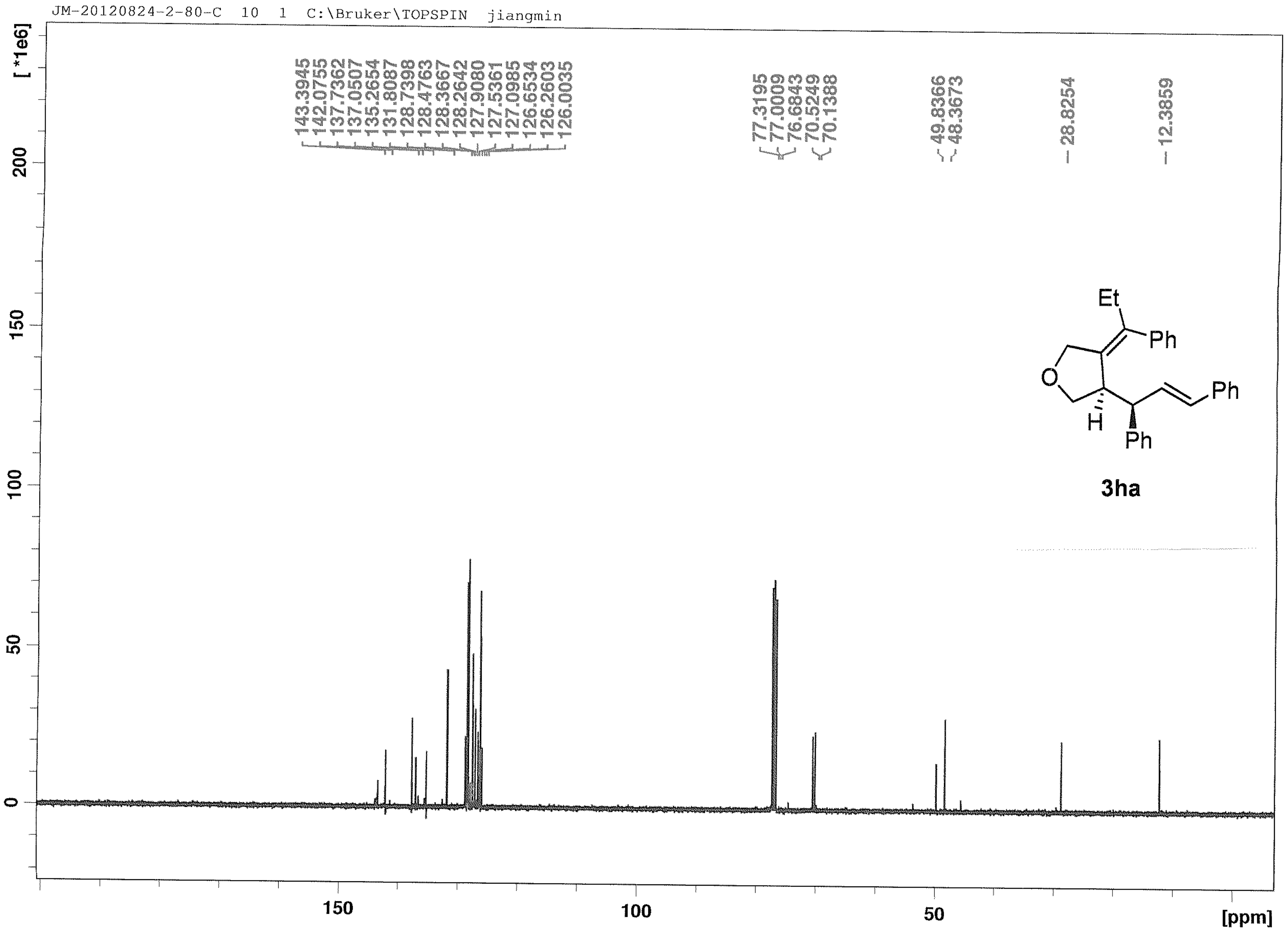
JM-20120824-2-80-C 10 1 C:\Bruker\TOPSPIN jiangmin
143.3945
142.0755
137.7362
137.0507
135.2654
131.8087
128.7398
128.4763
128.3667
128.2642
127.9080
127.5361
127.0985
126.6534
126.2603
126.0035
77.3195
77.0009
76.6843
70.5249
70.1388
49.8366
48.3673
28.8254
12.3859
[*1e6]
[ppm]
Et
Ph
O
Ph
H
Ph
3ha

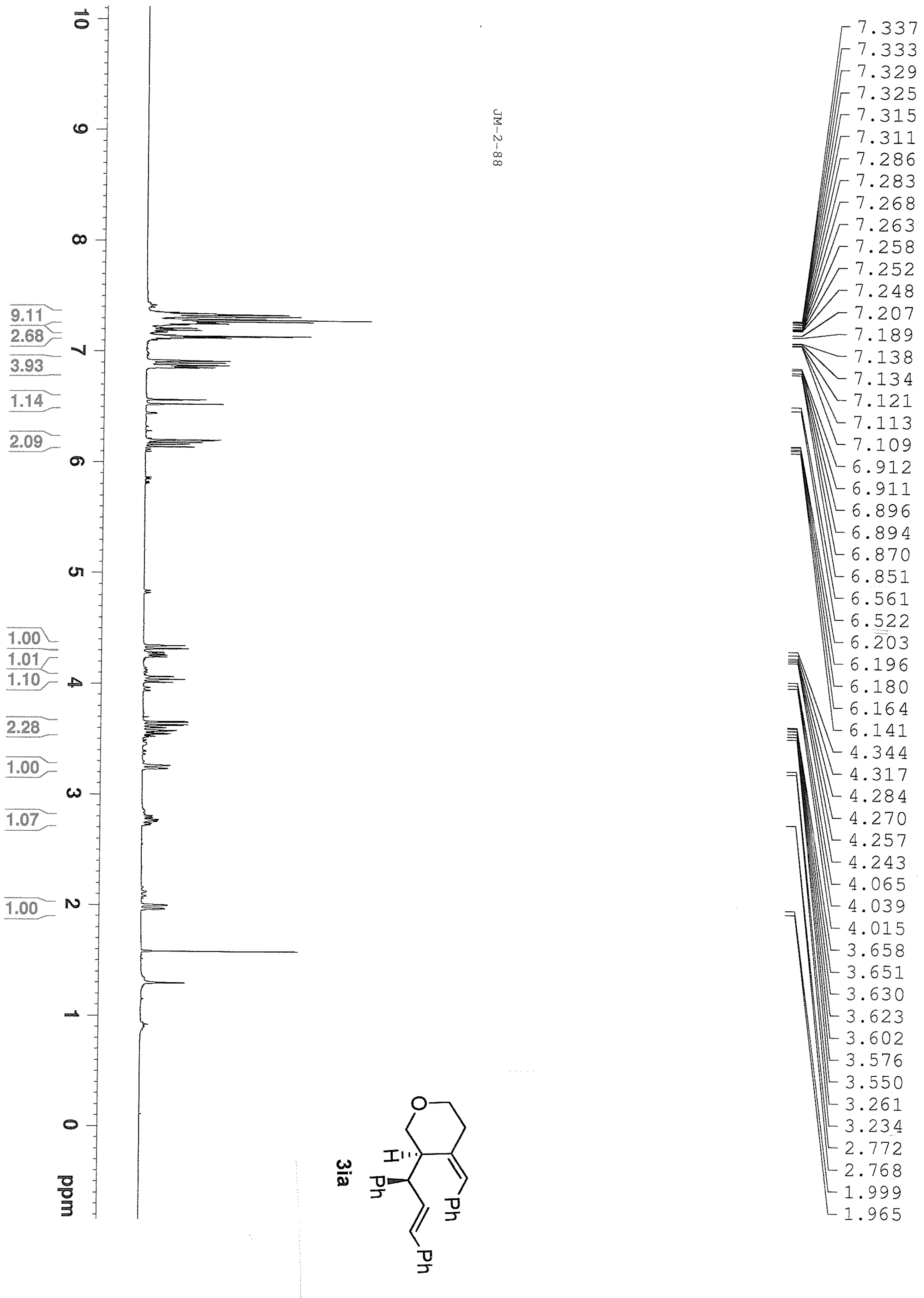
JM-2-88
7.337
7.333
7.329
7.325
7.315
7.311
7.286
7.283
7.268
7.263
7.258
7.252
7.248
7.207
7.189
7.138
7.134
7.121
7.113
7.109
6.912
6.911
6.896
6.894
6.870
6.851
6.561
6.522
6.203
6.196
6.180
6.164
6.141
4.344
4.317
4.284
4.270
4.257
4.243
4.065
4.039
4.015
3.658
3.651
3.630
3.623
3.602
3.576
3.550
3.261
3.234
2.772
2.768
1.999
1.965
9.11
2.68
3.93
1.14
2.09
1.00
1.01
1.10
2.28
1.00
1.07
1.00
ppm
3ia
Ph
Ph
Ph
H
O

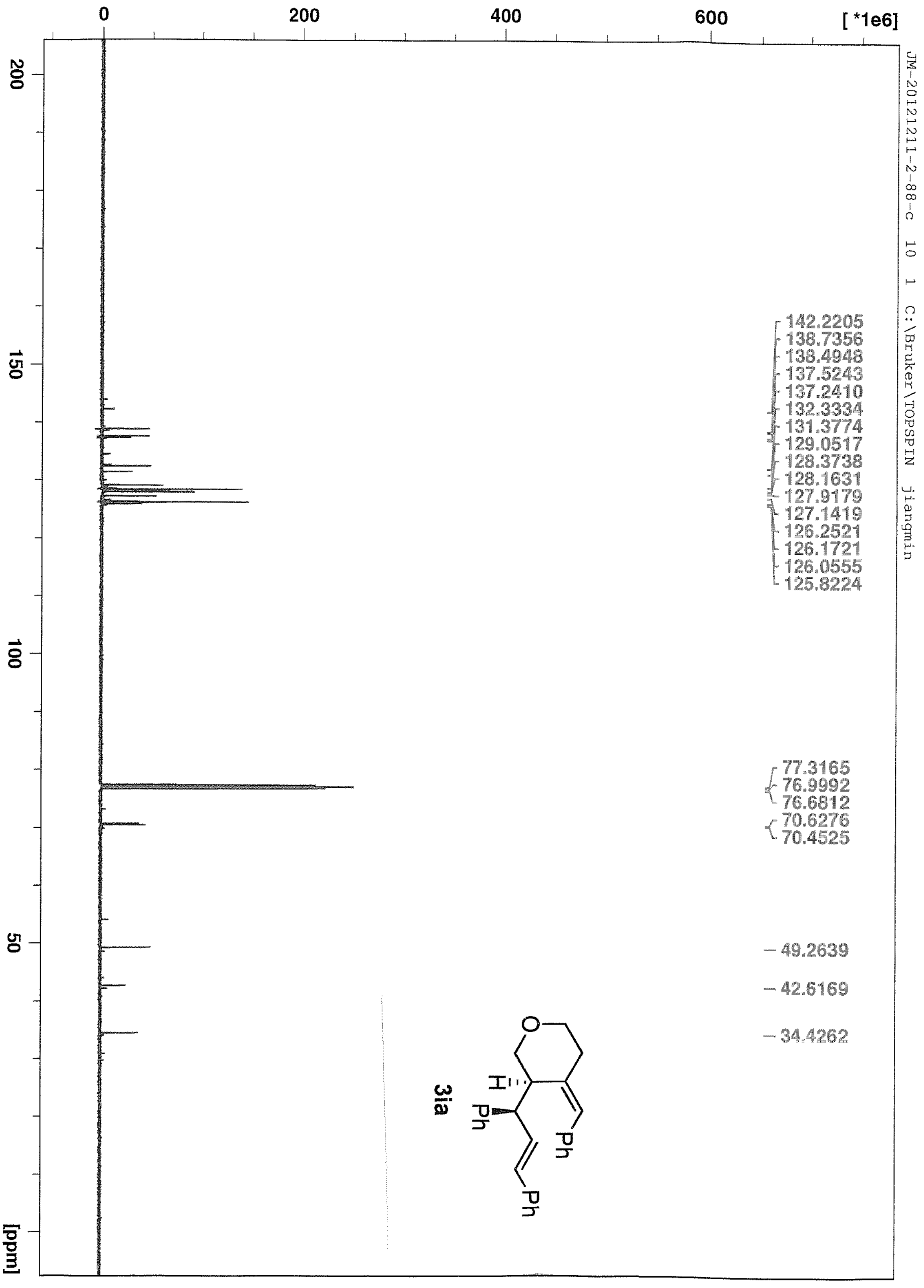

JM-20121211-2-88-c 10 1 C:\Bruker\TOPSPIN jiangmin
142.2205
138.7356
138.4948
137.5243
137.2410
132.3334
131.3774
129.0517
128.3738
128.1631
127.9179
127.1419
126.2521
126.1721
126.0555
125.8224
77.3165
76.9992
76.6812
70.6276
70.4525
49.2639
42.6169
34.4262
3ia
Ph
Ph
Ph
H
O
[*1e6]
[ppm]

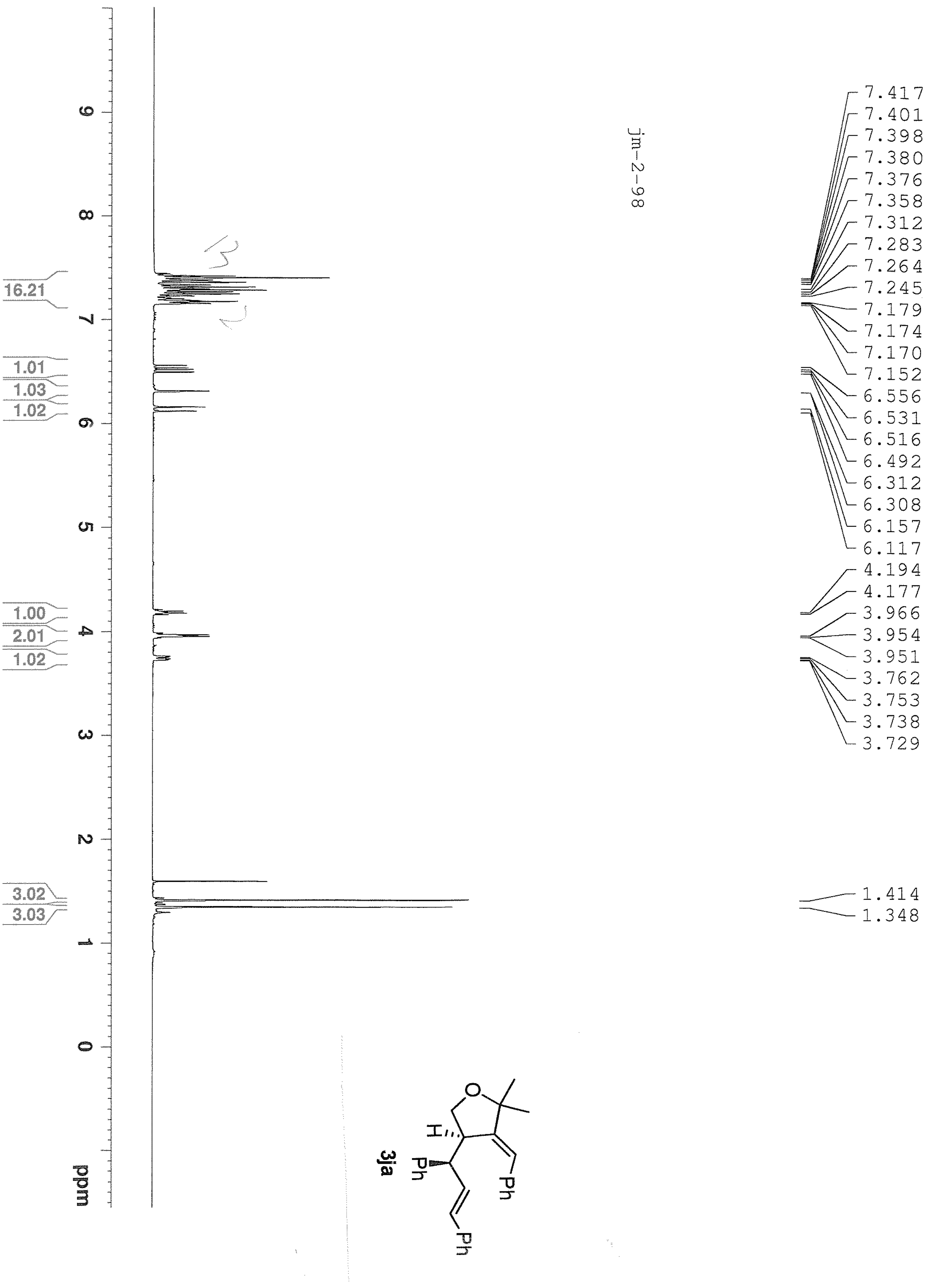
jm-2-98
7.417
7.401
7.398
7.380
7.376
7.358
7.312
7.283
7.264
7.245
7.179
7.174
7.170
7.152
6.556
6.531
6.516
6.492
6.312
6.308
6.157
6.117
4.194
4.177
3.966
3.954
3.951
3.762
3.753
3.738
3.729
1.414
1.348
16.21
1.01
1.03
1.02
1.00
2.01
1.02
3.02
3.03
9
8
7
6
5
4
3
2
1
0
ppm
3ja
Ph
Ph
Ph
H
O

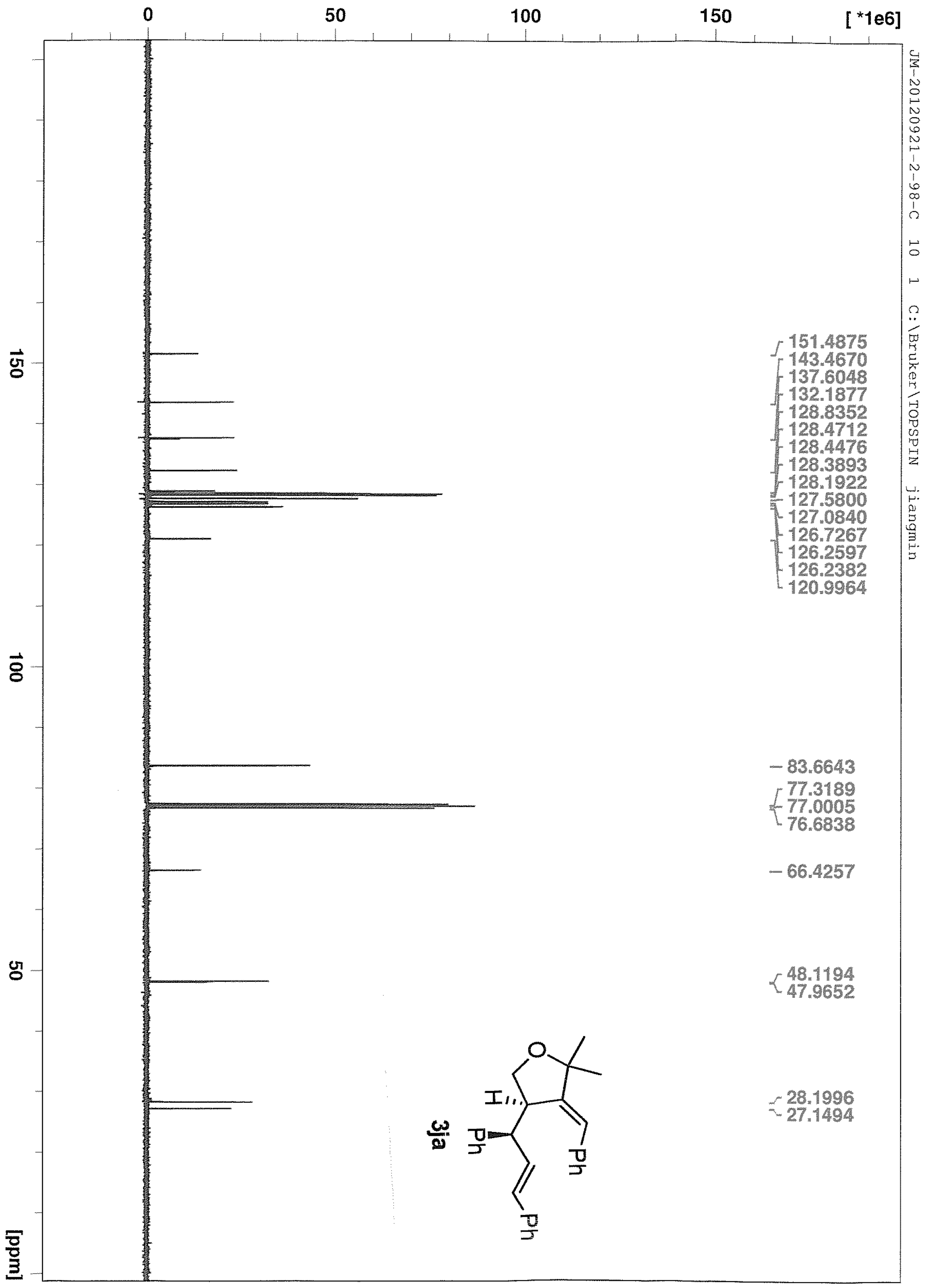
JM-20120921-2-98-C 10 1 C:\Bruker\TOPSPIN jiangmin
151.4875
143.4670
137.6048
132.1877
128.8352
128.4712
128.4476
128.3893
128.1922
127.5800
127.0840
126.7267
126.2597
126.2382
120.9964
83.6643
77.3189
77.0005
76.6838
66.4257
48.1194
47.9652
28.1996
27.1494
3ja
Ph
Ph
Ph
H
O
[ppm]
[*1e6]

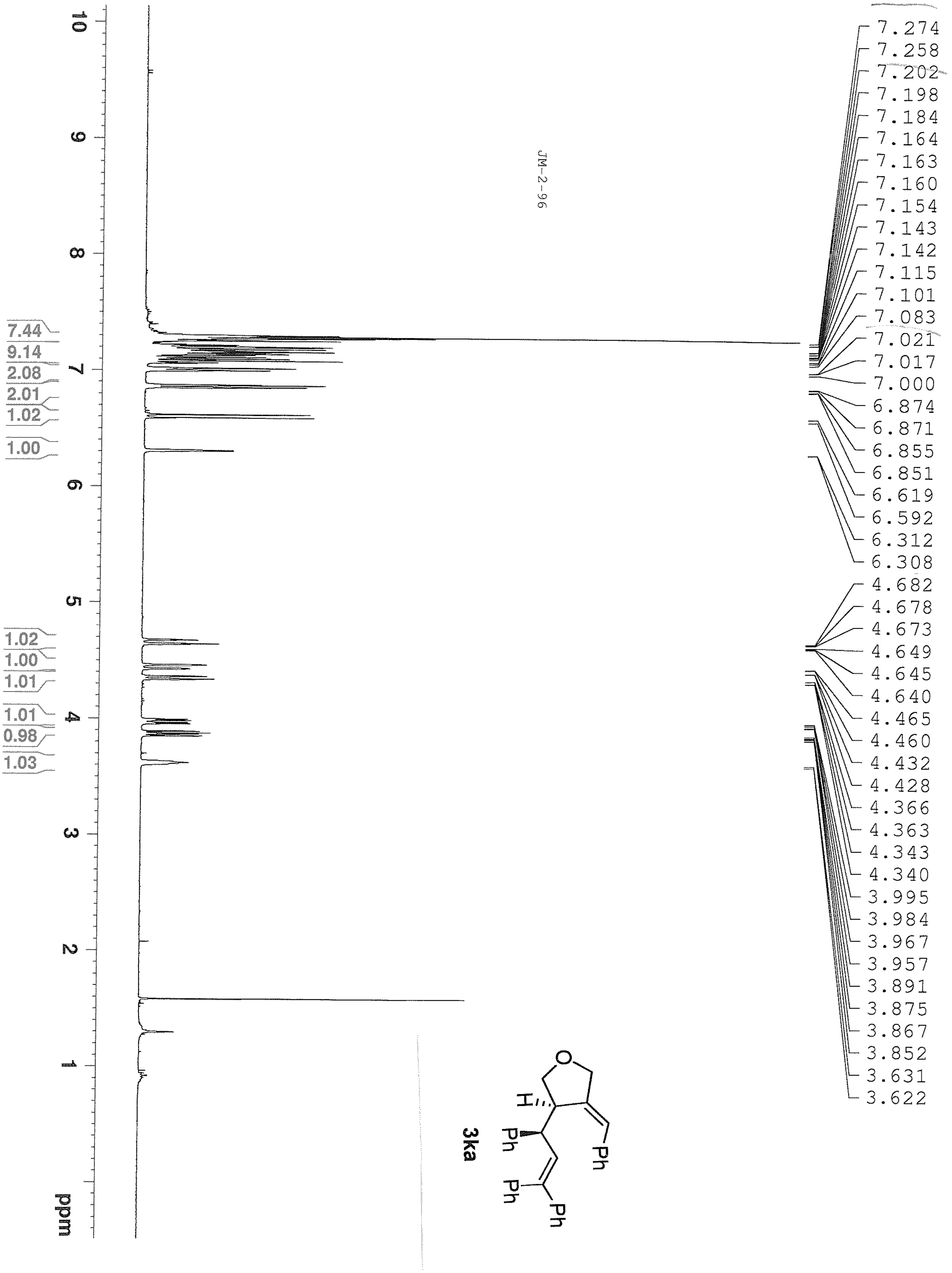

JM-2-96
7.274
7.258
7.202
7.198
7.184
7.164
7.163
7.160
7.154
7.143
7.142
7.115
7.101
7.083
7.021
7.017
7.000
6.874
6.871
6.855
6.851
6.619
6.592
6.312
6.308
4.682
4.678
4.673
4.649
4.645
4.640
4.465
4.460
4.432
4.428
4.366
4.363
4.343
4.340
3.995
3.984
3.967
3.957
3.891
3.875
3.867
3.852
3.631
3.622
7.44
9.14
2.08
2.01
1.02
1.00
1.02
1.00
1.01
1.01
0.98
1.03
10
9
8
7
6
5
4
3
2
1
ppm
Ph
Ph
Ph
Ph
H
O
3ka

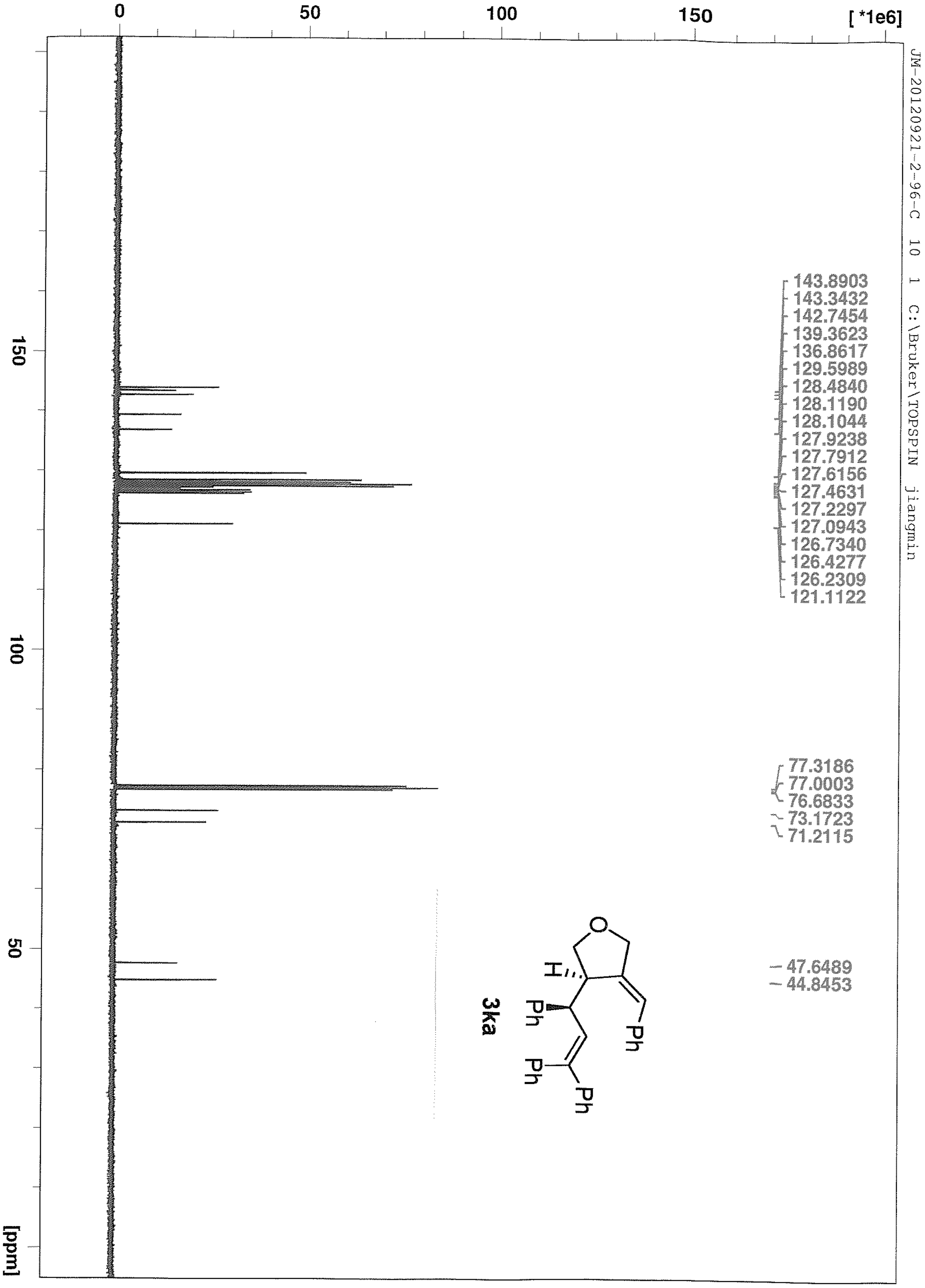

0
50
100
150
[*1e6]
JM-20120921-2-96-C 10 1 C:\Bruker\TOPSPIN jiangmin
143.8903
143.3432
142.7454
139.3623
136.8617
129.5989
128.4840
128.1190
128.1044
127.9238
127.7912
127.6156
127.4631
127.2297
127.0943
126.7340
126.4277
126.2309
121.1122
77.3186
77.0003
76.6833
73.1723
71.2115
47.6489
44.8453
3ka
[ppm]

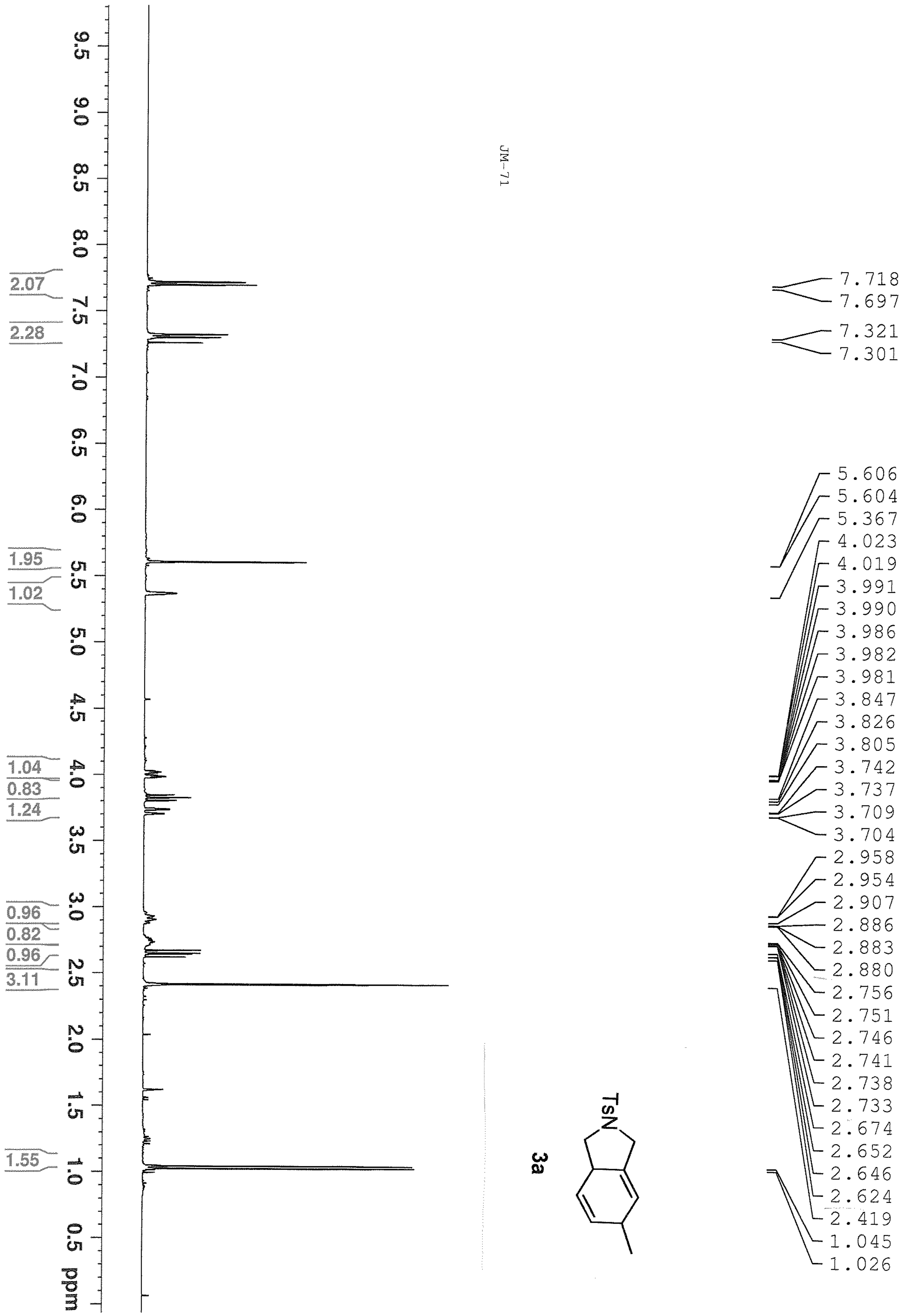
JM-71
7.718
7.697
7.321
7.301
5.606
5.604
5.367
4.023
4.019
3.991
3.990
3.986
3.982
3.981
3.847
3.826
3.805
3.742
3.737
3.709
3.704
2.958
2.954
2.907
2.886
2.883
2.880
2.756
2.751
2.746
2.741
2.738
2.733
2.674
2.652
2.646
2.624
2.419
1.045
1.026
2.07
2.28
1.95
1.02
1.04
0.83
1.24
0.96
0.82
0.96
3.11
1.55
9.5 9.0 8.5 8.0 7.5 7.0 6.5 6.0 5.5 5.0 4.5 4.0 3.5 3.0 2.5 2.0 1.5 1.0 0.5 ppm
TsN
3a

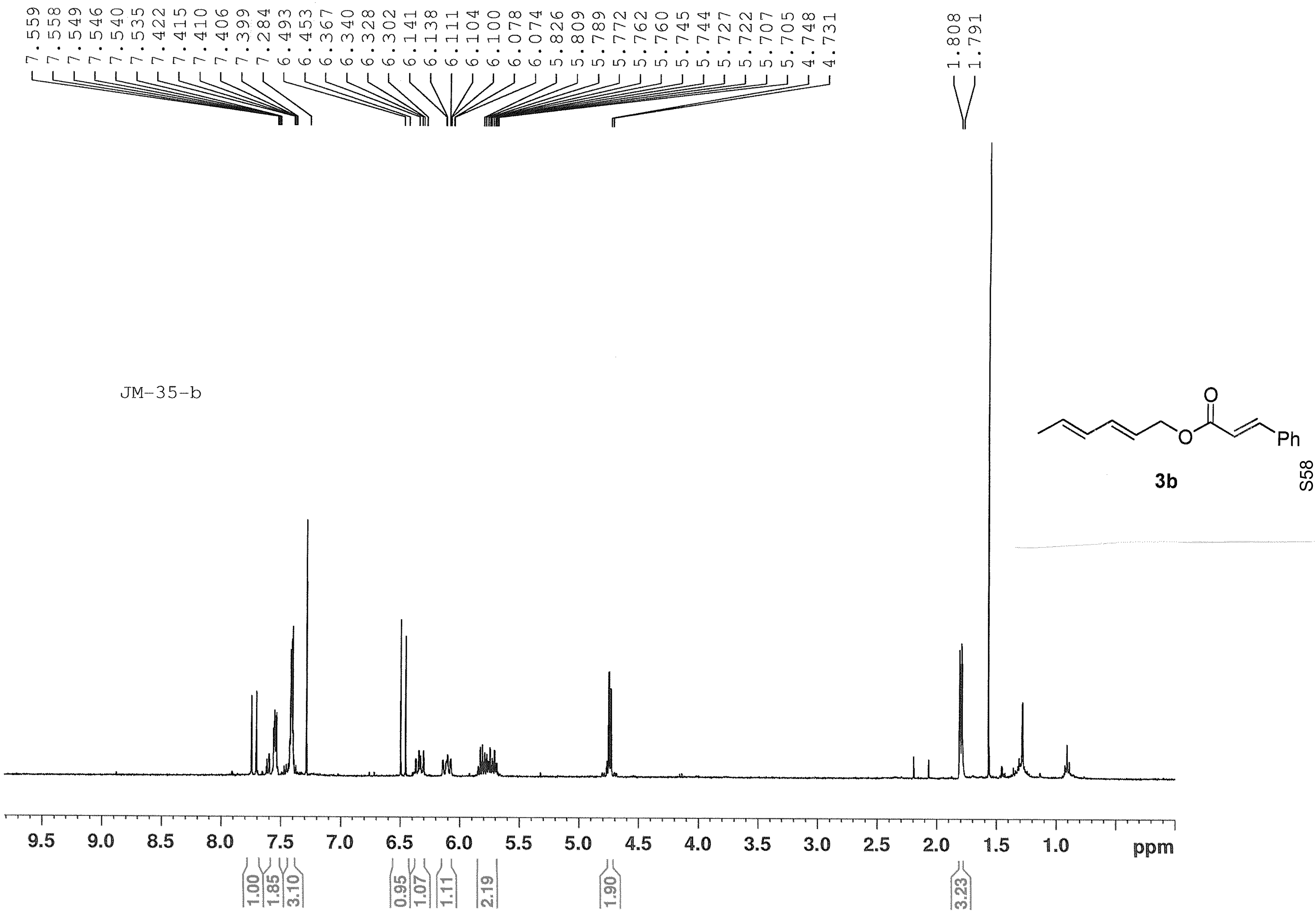
7.559
7.558
7.549
7.546
7.540
7.535
7.422
7.415
7.410
7.406
7.399
7.284
6.493
6.453
6.367
6.340
6.328
6.302
6.141
6.138
6.111
6.104
6.100
6.078
6.074
5.826
5.809
5.789
5.772
5.762
5.760
5.745
5.744
5.727
5.722
5.707
5.705
4.748
4.731
1.808
1.791
JM-35-b
O
O
Ph
9.5
9.0
8.5
8.0
7.5
7.0
6.5
6.0
5.5
5.0
4.5
4.0
3.5
3.0
2.5
2.0
1.5
1.0
ppm
1.00
1.85
3.10
0.95
1.07
1.11
2.19
1.90
3.23

3b

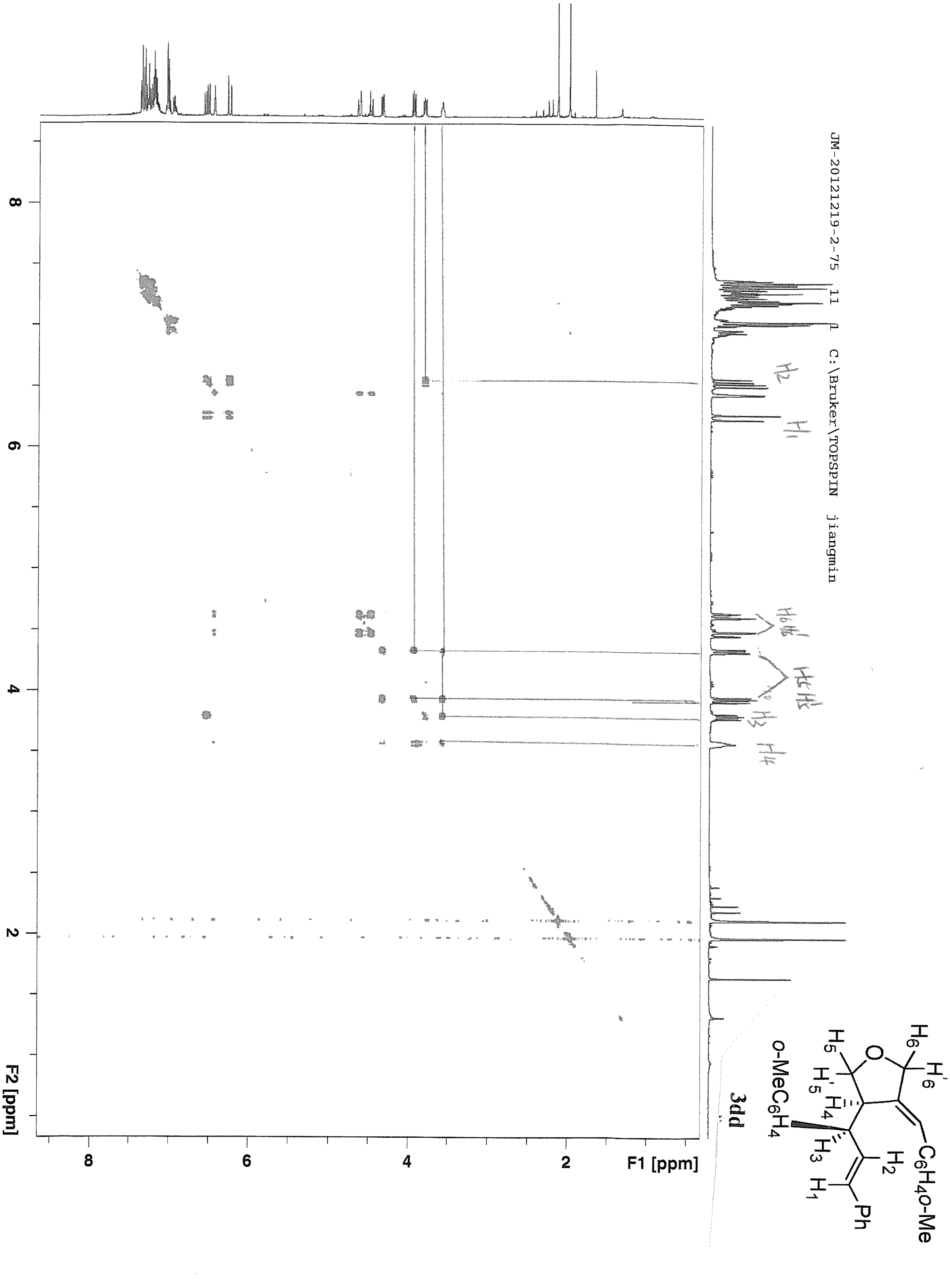
JM-20121219-2-75 11 1 C:\Bruker\TOPSPIN jiangmin
F1 [ppm]
F2 [ppm]
3dd
o-MeC6H4
C6H4o-Me
Ph
